# Associations of Meat and Fish Consumption With Conventional and Radiomics Cardiovascular Magnetic Resonance Phenotypes in the UK Biobank

**DOI:** 10.3389/fcvm.2021.667849

**Published:** 2021-05-05

**Authors:** Zahra Raisi-Estabragh, Celeste McCracken, Polyxeni Gkontra, Akshay Jaggi, Maddalena Ardissino, Jackie Cooper, Luca Biasiolli, Nay Aung, Stefan K. Piechnik, Stefan Neubauer, Patricia B. Munroe, Karim Lekadir, Nicholas C. Harvey, Steffen E. Petersen

**Affiliations:** ^1^National Institute for Health Research (NIHR) Barts Biomedical Research Centre, William Harvey Research Institute, Queen Mary University of London, Charterhouse Square, London, United Kingdom; ^2^Barts Heart Centre, St Bartholomew's Hospital, Barts Health National Health Service (NHS) Trust, London, United Kingdom; ^3^Departament de Matemàtiques and Informàtica, Universitat de Barcelona, Barcelona, Spain; ^4^Imperial College London, Sir Alexander Fleming Building, London, United Kingdom; ^5^Division of Cardiovascular Medicine, Radcliffe Department of Medicine, National Institute for Health Research Oxford Biomedical Research Centre, Oxford University Hospitals National Health Service Foundation Trust, University of Oxford, Oxford, United Kingdom; ^6^Medical Research Council (MRC) Lifecourse Epidemiology Unit, University of Southampton, Southampton, United Kingdom; ^7^National Institute for Health Research (NIHR) Southampton Biomedical Research Centre, University of Southampton and University Hospital Southampton National Health Service (NHS) Foundation Trust, Southampton, United Kingdom

**Keywords:** diet, meat, cardiovascular magnetic resonance, radiomics, cardiovascular disease, disease prevention, population health

## Abstract

**Background:** Greater red and processed meat consumption has been linked to adverse cardiovascular outcomes. However, the impact of these exposures on cardiovascular magnetic resonance (CMR) phenotypes has not been adequately studied.

**Objective:** We describe novel associations of meat intake with cardiovascular phenotypes and investigate underlying mechanisms through consideration of a range of covariates.

**Design:** We studied 19,408 UK Biobank participants with CMR data available. Average daily red and processed meat consumption was determined through food frequency questionnaires and expressed as a continuous variable. Oily fish was studied as a comparator, previously associated with favourable cardiac outcomes. We considered associations with conventional CMR indices (ventricular volumes, ejection fraction, stroke volume, left ventricular mass), novel CMR radiomics features (shape, first-order, texture), and arterial compliance measures (arterial stiffness index, aortic distensibility). We used multivariable linear regression to investigate relationships between meat intake and cardiovascular phenotypes, adjusting for confounders (age, sex, deprivation, educational level, smoking, alcohol intake, exercise) and potential covariates on the causal pathway (hypertension, hypercholesterolaemia, diabetes, body mass index).

**Results:** Greater red and processed meat consumption was associated with an unhealthy pattern of biventricular remodelling, worse cardiac function, and poorer arterial compliance. In contrast, greater oily fish consumption was associated with a healthier cardiovascular phenotype and better arterial compliance. There was partial attenuation of associations between red meat and conventional CMR indices with addition of covariates potentially on the causal pathway, indicating a possible mechanistic role for these cardiometabolic morbidities. However, other associations were not altered with inclusion of these covariates, suggesting importance of alternative biological mechanisms underlying these relationships. Radiomics analysis provided deeper phenotyping, demonstrating association of the different dietary habits with distinct ventricular geometry and left ventricular myocardial texture patterns.

**Conclusions:** Greater red and processed meat consumption is associated with impaired cardiovascular health, both in terms of markers of arterial disease and of cardiac structure and function. Cardiometabolic morbidities appeared to have a mechanistic role in the associations of red meat with ventricular phenotypes, but less so for other associations suggesting importance of alternative mechanism for these relationships.

## Introduction

Multiple epidemiological studies have demonstrated associations between greater meat consumption and worse cardiovascular outcomes (1–4). In particular, higher red and processed meat intake has been associated with greater burden of atherosclerosis (5), increased risk of incident ischaemic cardiovascular events (6), and heart failure (7). Furthermore, murine studies link greater red meat consumption with pathological ventricular remodelling and heart failure phenotypes (8).

It has been proposed, that these relationships may be mediated through adverse cardiometabolic alterations (9, 10). More recently, evidence has emerged for novel causal pathways relating to cross-system interactions with the gut microbiome (11).

We studied novel associations between red and processed meat consumption and measures of cardiovascular structure and function in the UK Biobank, including conventional cardiovascular magnetic resonance (CMR) metrics, novel CMR radiomics features, and measures of arterial compliance. We considered associations between oily fish intake as a comparator previously linked with favourable cardiovascular endpoints (6). We considered a wide range of confounders and covariates that may lie on the causal pathway.

## Methods

### Setting and Study Population

The UK Biobank is a cohort study of over 500,000 participants. Recruitment was between 2006 and 2010 through postal invite of UK residents aged 40–69 years identified through National Health Service (NHS) registers. Individuals who were unable to consent or complete baseline assessment due to illness or discomfort were not recruited. Baseline assessment of participants comprised characterisation of socio-demographics, lifestyle, environmental factors, medical history, and a range of physical measures (12). The protocol is publicly available (13). The UK Biobank imaging study, which includes detailed CMR imaging, was launched in 2015 and aims to scan a random subset of 100,000 participants (~50,000 completed, March 2021) (14).

### Ethics

This study complies with the Declaration of Helsinki; the work was covered by the ethical approval for UK Biobank studies from the NHS National Research Ethics Service on 17th June 2011 (Ref 11/NW/0382) and extended on 10th May 2016 (Ref 16/NW/0274) with written informed consent obtained from all participants.

### Definition of Meat Consumption Variables

Dietary intake was assessed using a self-report food frequency questionnaire at the baseline UK Biobank visit. Participants were asked to estimate their weekly intake of a range of food items over the preceding year. We considered beef, pork, lamb/mutton, processed meat, and oily fish consumption (Supplementary Table 1). We considered each type of red meat separately (beef, lamb/mutton, pork) and also as a composite category of “unprocessed red meat.” Processed meat included products such as bacon, ham, sausages, meat pies, kebabs, and burgers. Reported portion frequencies were converted into probabilities of daily consumption and multiplied by standard portion sizes (15) to derive average daily consumption in grams. Thus, we were able to consider the meat exposures as continuous variables, as has been published previously using this dataset (16).

### Measures of Cardiac Structure and Function

#### Conventional CMR Indices

CMR scans were performed in dedicated UK Biobank imaging centres using 1.5 Tesla scanners (MAGNETOM Aera, Syngo Platform VD13A, Siemens Healthcare, Erlangen, Germany) according to a pre-defined acquisition protocol, which is detailed in a separate publication (17). Assessment of the left and right ventricles (LV, RV) included a complete short axis stack acquired using balanced steady-state free precession sequences. The first 5,000 CMR scans were manually analysed according to a pre-defined segmentation protocol (18) using CVI^42^ post-processing software (Version 5.1.1, Circle Cardiovascular Imaging Inc., Calgary, Canada). Briefly, LV endocardial and epicardial borders were manually contoured in end-diastole and end-systole in the short axis view. The first phase of acquisition was selected as end-diastole. End-systole was defined as the phase with the smallest mid-ventricular LV intra-cavity blood pool as determined by visual inspection. The most basal slice for the LV was selected when at least half of the LV blood pool was surrounded by myocardium. LV papillary muscles were excluded from LV mass. RV endocardial borders were traced in end-diastole and end-systole with volumes below the pulmonary valve plane considered as part of the RV. This ground truth manual analysis dataset was used to develop a fully automated image analysis pipeline with inbuilt quality control, which has been applied to the first 20,000 UK Biobank CMR studies (19). Details of reproducibility performance of the automated algorithm are available in a dedicated publication (18, 19). For the present analysis, data was available from 19,408 CMR studies, including the following metrics: LV and RV volumes in end-diastole and end-systole, LV and RV ejection fraction, LV and RV stroke volume, and LV mass.

#### Novel CMR Radiomics Features

CMR radiomics is a novel image analysis technique permitting computation of multiple indices of shape and texture (20). Radiomics features provide information that is complementary and potentially incremental to conventional CMR indices (20). We used contours from conventional CMR analysis, with image segmentation as described above, on short axis cine images (19) to define three regions of interest (ROIs) for radiomics analysis in end-diastole and end-systole: RV cavity, LV cavity, and LV myocardium. We extracted shape features from the RV and LV cavity ROIs. From the LV myocardium, we extracted first order and texture radiomics features. Radiomics features were extracted using the PyRadiomics open source platform (21). The full list of radiomics features extracted is presented in Supplementary Table 2. To reduce variation of image signal intensities relating to the acquisition process, we performed intensity normalisation of CMR images through histogram matching, using as reference one of the studies from the dataset (22). For grey level discretisation, we used a fixed bin width of 25 intensity values.

### Measures of Arterial Compliance

#### Aortic Distensibility

Aortic distensibility is a measure of local aortic compliance. Lower aortic distensibility indicates poorer vascular health and is a marker of arterial disease (23). Aortic distensibility may be estimated by considering the relative cross-sectional area change of the thoracic aorta from diastole to systole on transverse cine CMR images (24). In previous work, we derived aortic distensibility using data generated from a fully automated image analysis pipeline applied to the first 20,000 UK Biobank CMR studies, details of the automated pipeline are presented in a separate publication (25).

#### Arterial Stiffness Index

Arterial stiffness index (ASI) provides an estimate of large artery stiffness derived from a pulse waveform contour (23). Higher ASI indicates lower arterial compliance and is associated with poorer cardiovascular, in particular ischaemic, outcomes (23, 26). ASI was measured at both baseline and imaging visits using finger photoplethysmography with the PulseTrace PCA2 (CareFusion, USA) device, according to a standardised protocol (27).

### Statistical Analysis

Statistical analysis was performed using R Version 3.6.2 (28) and RStudio Version 1.2.5019 (29). We estimated the association of the dietary intake exposures with cardiovascular metrics in individual multivariable linear regression models. To allow derivation of easily assimilated effect sizes, we report change in cardiovascular metric per 100 g increase in daily meat consumption alongside corresponding 95% confidence intervals (CIs) and *p*-values. For ASI, we assessed associations with measures taken at both baseline and imaging visits. We identified significant interval change in ASI from baseline to imaging. Therefore, we also considered “change in ASI” as a separate outcome, expressed using standardised residuals derived from regression of ASI at imaging on ASI at baseline. The average time interval between baseline and imaging assessment was 7.5 years in the CMR set and 8.2 years in the ASI set.

**Figure 1:**
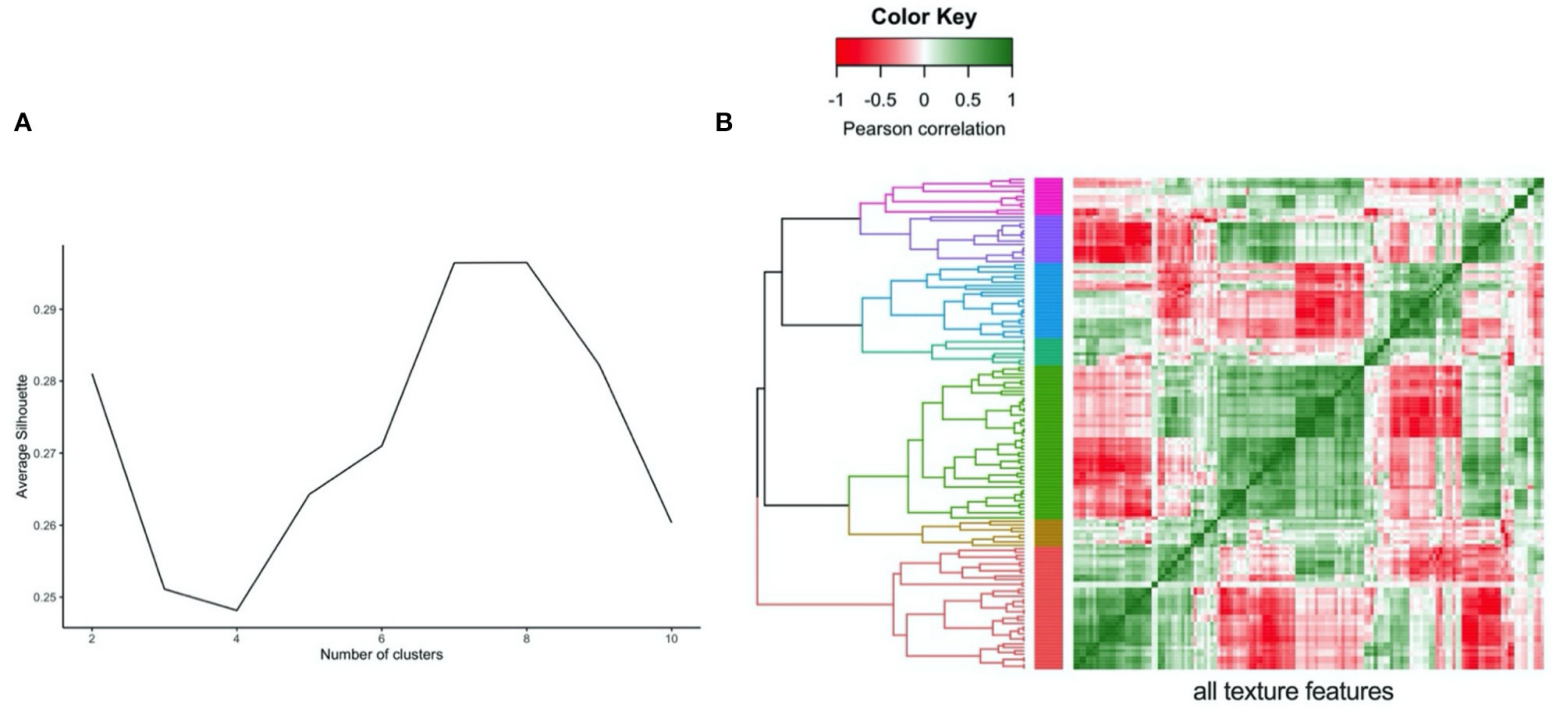
Illustration of clustering method (hierarchical) and approach to defining the number of clusters (average silhouette approach) for the LV myocardium radiomics texture features. **(A)** Average silhouette statistic for complete-linkage hierarchical clustering of texture feature correlations. The silhouette statistic reflects the average distance between data points in the same cluster compared against average data points in other clusters and allows judgement of the optimal number of clusters within a sample, such that distance between datapoints within clusters are minimised whilst maximising distance with datapoints from other clusters. We computed average silhouette statistic for 2 to 10 clusters. Maximum silhouette statistic was observed with 7 and 8 clusters. Hence, we take 7 clusters as representing the optimal number of clusters within our samples. **(B)** Correlation heatmap, rows and columns correspond to all texture features creating grid with all possible pairs of texture features, grid colour corresponds to Pearson Correlation between pair of features at that point. Grid rows re-ordered by hierarchical clustering of correlations with tree coloured for optimal seven cluster cut of the tree.

In order to compare the magnitude of change across different radiomics features, prior to regression analysis, we performed z-score normalisation of the features. This resulted in calculation of standardised beta coefficients per 100 g daily increase in meat/fish intake. As the number of texture features extracted from the LV myocardium was large (*n* = 144), we performed cluster analysis (Figure 1) to group inter-related features (30). We hierarchically clustered features using complete linkage on Pearson correlation distance between features. We determined the optimal number of clusters by computing the average silhouette, a measure of cluster consistency using the cluster package in R (30). The silhouette statistic reflects the average distance between data points in the same cluster compared against average data points in other clusters and allows judgement of the optimal number of clusters within a sample, such that distance between datapoints within clusters are minimised whilst maximising distance with datapoints from other clusters. We computed average silhouette statistic for 2 to 10 clusters. Maximum silhouette statistic was observed with 7 and 8 clusters. Hence, we take 7 clusters as representing the optimal number of clusters within our samples (Figure 1). We assigned descriptive names to each cluster based on properties represented by its constituent features. Thus, for the texture features, we present the mean beta-coefficient and 95% CIs for each cluster for the different meat/fish exposures. We compare effects between exposure categories through testing for the difference in mean beta coefficients using Kruskal–Wallis statistical testing followed by Dunn's correction for multiple comparisons. As radiomics is a relatively new approach, we provide a brief guide to radiomics and specific guidance on interpretation of results from this analysis in Appendix.

**Figure 2:**
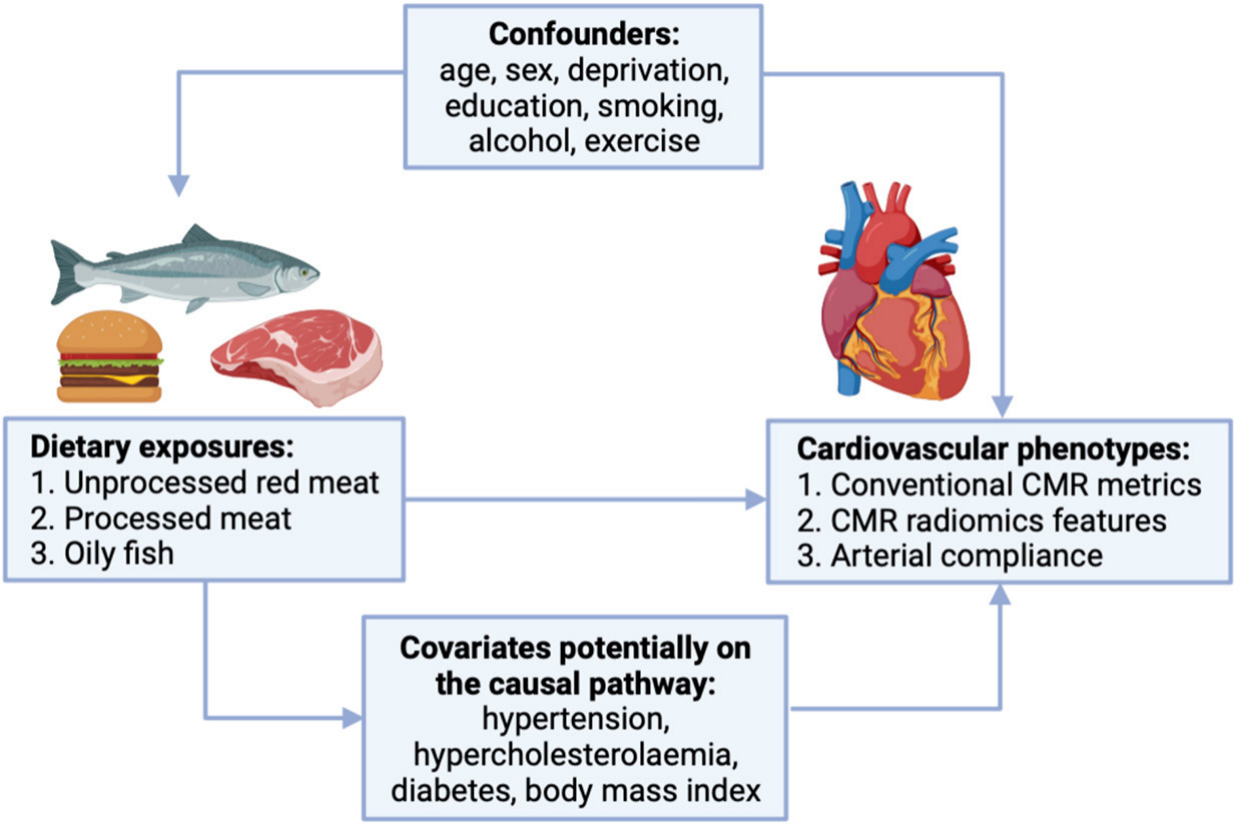
Covariates considered in the relationship between red and processed meat consumption and cardiovascular phenotypes. CMR, cardiovascular magnetic resonance.

We selected covariates based on association with both exposure and outcome in preliminary analyses and existing literature (Figure 2). We adjust for potential confounders (age, sex, material deprivation, education, smoking, alcohol intake, exercise) in our main models to estimate the magnitude of the exposure-outcome associations. We identified hypertension, hypercholesterolaemia, diabetes, and body mass index (BMI) as covariates potentially on the causal pathway (Figure 2). To test the impact of these variables, we estimated associations with additional inclusion of these factors in the models, with the expectation that covariates on the causal pathway would attenuate exposure-outcome associations.

Educational level, smoking status, and alcohol intake frequency were based on self-report. Material deprivation is reported as the Townsend index, a measure of deprivation relative to national averages (31). A continuous value for the amount of physical activity measured in metabolic equivalent (MET) minutes/week was calculated by weighting different types of activity (walking, moderate, or vigorous) by its energy requirements using values derived from the International Physical Activity Questionnaire (IPAQ) study (32). BMI was calculated from height and weight. Diabetes was ascertained from self-report of the diagnosis, self-reported use of “medication for diabetes,” or serum glycosylated haemoglobin >48 mmol/mol. Hypertension was coded based on self-report of the diagnosis or self-reported use of “medication for high blood pressure.” Hypercholesterolaemia was coded based on self-report of the diagnosis, self-reported use of “medication for high cholesterol,” or serum total cholesterol >7 mmol/L.

## Results

### Baseline Population Characteristics

CMR data was available for 19,408 participants. Mean age was 55.0 (±7.5) years, 52.1% were women (Table 1). The majority 97% (*n* = 18,810) were of White ethnic background; Black, Asian, and Other ethnicities made up 0.5%, 1.0%, and 1.5% of the analysis sample, respectively. The cohort was predominantly healthy, with only 5.5% (*n* = 1,062) having a history of pre-existing cardiovascular disease (ischaemic and non-ischaemic heart diseases, valvular heart disease, significant arrhythmias). The rates of cardiometabolic morbidities were also lower than the general population, in line with previous analyses of the UK Biobank (33, 34). The prevalence of hypertension, hypercholesterolaemia, diabetes, and smoking were 13.9%, 23.0%, 3.1%, and 6.4%, respectively. Average red meat intake (lamb/mutton, beef, and pork combined) was 22.3 (±15.2) g/day; beef was the most frequently eaten of the red meat types. Average intake of processed meat and oily fish were 15.7 (±15.0) g/day and 11.7 (±10.8) g/day, respectively.

**Table 1:** Baseline population characteristics.

**Male**	**9,303 (47.9%)**
**Female**	**10,105 (52.1%)**
**Age (years)**	**55.0 (±7.5)**
**Townsend deprivation index**	**−2.0 (±2.6)**
**Body mass index (kg/m^2^)**	**26.6 (±4.2)**
**Smoking (current smoker)**	**1,238 (6.4%)**
**Diabetes**	**606 (3.1%)**
**Hypertension**	**2,690 (13.9%)**
**Hypercholesterolaemia**	**4,464 (23.0%)**
**IPAQ score (METS/week)**	**1,525.00 [2,396.25]**
**Educational level**	
**Left school age 14 or younger without qualifications**	**53 (0.3%)**
**Left school age 15 or older without qualifications**	**1,394 (7.2%)**
**High school diploma**	**2,679 (13.8%)**
**Sixth form qualification**	**1,114 (5.7%)**
**Professional qualification**	**5,506 (28.4%)**
**Higher education University degree**	**8,456 (43.6%)**
**Alcohol intake frequency**	
**Never**	**954 (4.9%)**
**Special occasions only**	**1,587 (8.2%)**
**1–3 times a month**	**2,103 (10.8%)**
**1–2 times a week**	**4,997 (25.7%)**
**3–4 times a week**	**5,496 (28.3%)**
**Daily or almost daily**	**4,260 (21.9%)**
**Unprocessed red meat intake (grams/day)**	**22.3 (± 15.2)**
**Beef**	**9.5 (± 9.0)**
**Lamb**	**6.3 (± 5.4)**
**Pork**	**6.5 (± 5.9)**
**Processed meat intake (grams/day)**	**15.7 (± 15.0)**
**Oily fish intake (grams/day)**	**11.7 (± 10.8)**

*Results are frequencies and percentages for categorical variables; mean (standard deviation) or median [interquartile range] for continuous variable. IPAQ, international physical activity questionnaire; METS, metabolic equivalents*.

### Association of Meat and Fish Intake With Conventional CMR Indices

Greater consumption of red and processed meat was associated with smaller LV volumes in end-diastole and end-systole, higher LV mass, and lower LV stroke volume (Table 2). Greater oily fish consumption was associated with larger LV volumes in end-diastole and end-systole, greater LV mass, and higher LV stroke volume (Table 2). The same pattern of remodelling was observed in the RV, with greater red and processed meat intake linked to smaller ventricular volumes and lower stroke volume, whilst greater oily fish consumption was associated with larger cavity volumes and higher stroke volume (Supplementary Table 3). These relationships were consistent across the different red meat types for both the LV and RV indices (Supplementary Table 3). There was attenuation of associations between unprocessed red meat with all CMR indices other than LV mass with addition of cardiometabolic covariates, whilst associations with processed meat and oily fish remained largely unchanged (Supplementary Table 4).

**Table 2:** Multivariable linear regression models showing change in LV conventional CMR indices per 100 g increase in daily meat/fish consumption.

	**LVEDVi** ** (ml/m^**2**^)**	**LVESVi** ** (ml/m^**2**^)**	**LVEF** ** (%)**	**LVSVi (ml/m^**2**^)**	**LVMi** ** (g/m^**2**^)**
Unprocessed red meat	−2.18^*^ [−3.36, −1.00] 2.91 × 10^−4^	−0.96^*^ [−1.70, −0.23] 0.01	0.041 [−0.50, 0.59] 0.88	−1.22^*^ [−1.97, −0.47] 1.50 × 10^−3^	1.57^*^ [0.91, 2.23] 3.19 × 10^−6^
Processed meat	−2.88^*^ [−4.12, −1.65] 4.70 × 10^−6^	−1.12^*^ [−1.89, −0.35] 4.2 × 10^−3^	−0.12 [−0.69, 0.45] 0.68	−1.77^*^ [−2.55, −0.98] 1.06 × 10^−5^	0.57 [−0.12, 1.26] 0.11
Oily fish	4.13^*^ [2.46, 5.80] 1.28 × 10^−6^	1.75^*^ [0.71, 2.79] 9.68 × 10^−4^	0.10 [−0.67, 0.88] 0.79	2.38^*^ [1.32, 3.45] 1.13 × 10^−5^	2.38^*^ [1.44, 3.31] 6.40 × 10^−7^

*Each cell represents a separate model, adjusted for: age, sex, social deprivation, educational level, smoking, alcohol intake, and exercise level. Results are presented as beta coefficient [95% confidence interval] p-value. CMR, cardiovascular magnetic resonance; LV, left ventricle; LVEDV, LV end-diastolic volume; LVESV, LV end-systolic volume; LVEF, LV ejection fraction; LVM, LV mass; LVSV, LV stroke volume; i denotes indexation to body surface area*.

* *p < 0.05*.

### Association of Meat and Fish Intake With LV and RV Radiomics Shape Features

**Figure 3:**
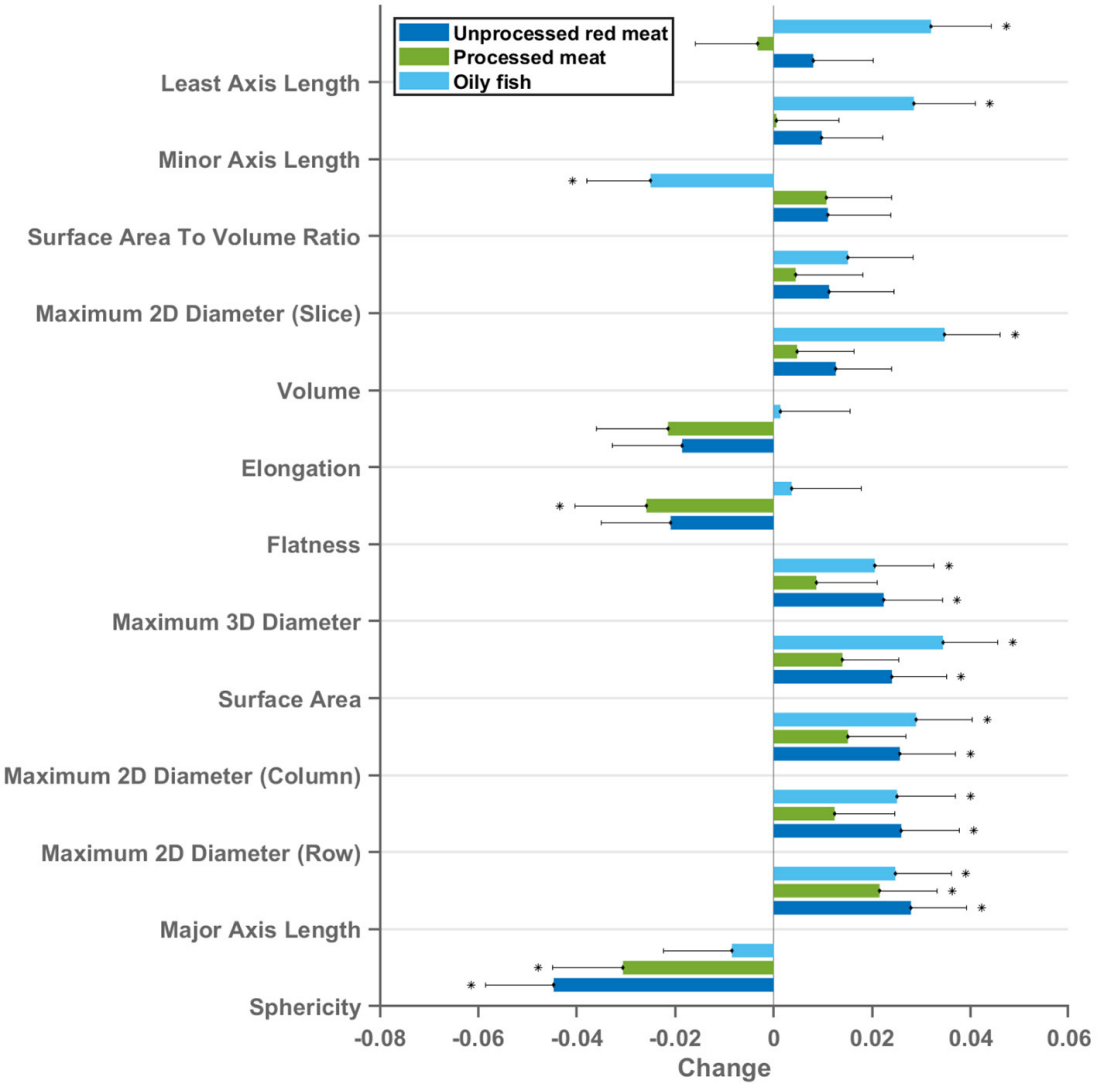
Multivariable linear regression models showing change in LV cavity CMR shape radiomics (end-diastole) per 100 g increase in daily meat consumption. Each bar represents standardised beta coefficients corresponding to the indicated radiomics shape feature. Each bar is from a separate model adjusted for age, sex, social deprivation, educational level, smoking, alcohol intake, and exercise level. Black lines represent half-length of confidence interval for the corresponding bar. Asterix denotes significant association. Bonferroni adjusted significance threshold *p* = 0.001 (corrected for 39 comparisons). CMR, cardiovascular magnetic resonance; LV, left ventricle.

13 radiomics shape features were extracted from each ventricle (LV and RV) in end-diastole and end-systole. Greater oily fish consumption was associated with significantly larger LV volumes, larger cavity dimensions in both the short and long axis, and greater surface area of the LV cavity (Figures 3, 6). Interpreted in conjunction with the previously observed association with higher LV stroke volume (indicating better myocardial function), these findings are in keeping with healthy cardiac structure and function. Greater red and processed meat intake were associated with lower “flatness” [values range between 1 (sphere-like) and 0 (a flat object)], lower “elongation” [values range between 1 (non-elongated) and 0 (a maximally elongated object: i.e., a 1 dimensional line)], and lower “sphericity” (a dimensionless measure of the roundness of the ROI relative to a sphere. The value range is 0 < sphericity ≤ 1, where a value of 1 indicates a perfect sphere). Thus, greater red and processed meat intake is associated with a more elongated LV shape (Figures 3, 6). In contrast, greater oily fish consumption showed trends towards greater “elongation” and “flatness” (not statistically significant) indicating a more spherical chamber.

Considering these relationships as well as association with lower LV stroke volume, the overall picture suggests that greater red and processed meat intake is associated with of an unhealthy LV phenotype with impaired myocardial contractility. The pattern of associations of cardiac structure and function metrics with greater oily fish intake is distinctly different to that of the meat exposures and, overall, suggestive of a healthy phenotype.

The same pattern of associations was observed across the different red meat types in end-diastole and end-systole (Supplementary Figure 1) and consistent associations were observed with RV shape radiomics (Supplementary Figure 2). Results from individual associations between meat and fish exposures and LV and RV radiomics features in end-diastole and end-systole are presented in Supplementary Tables 5–8.

### Association of Meat and Fish Intake With LV Myocardium Radiomics First-Order Features

**Figure 4:**
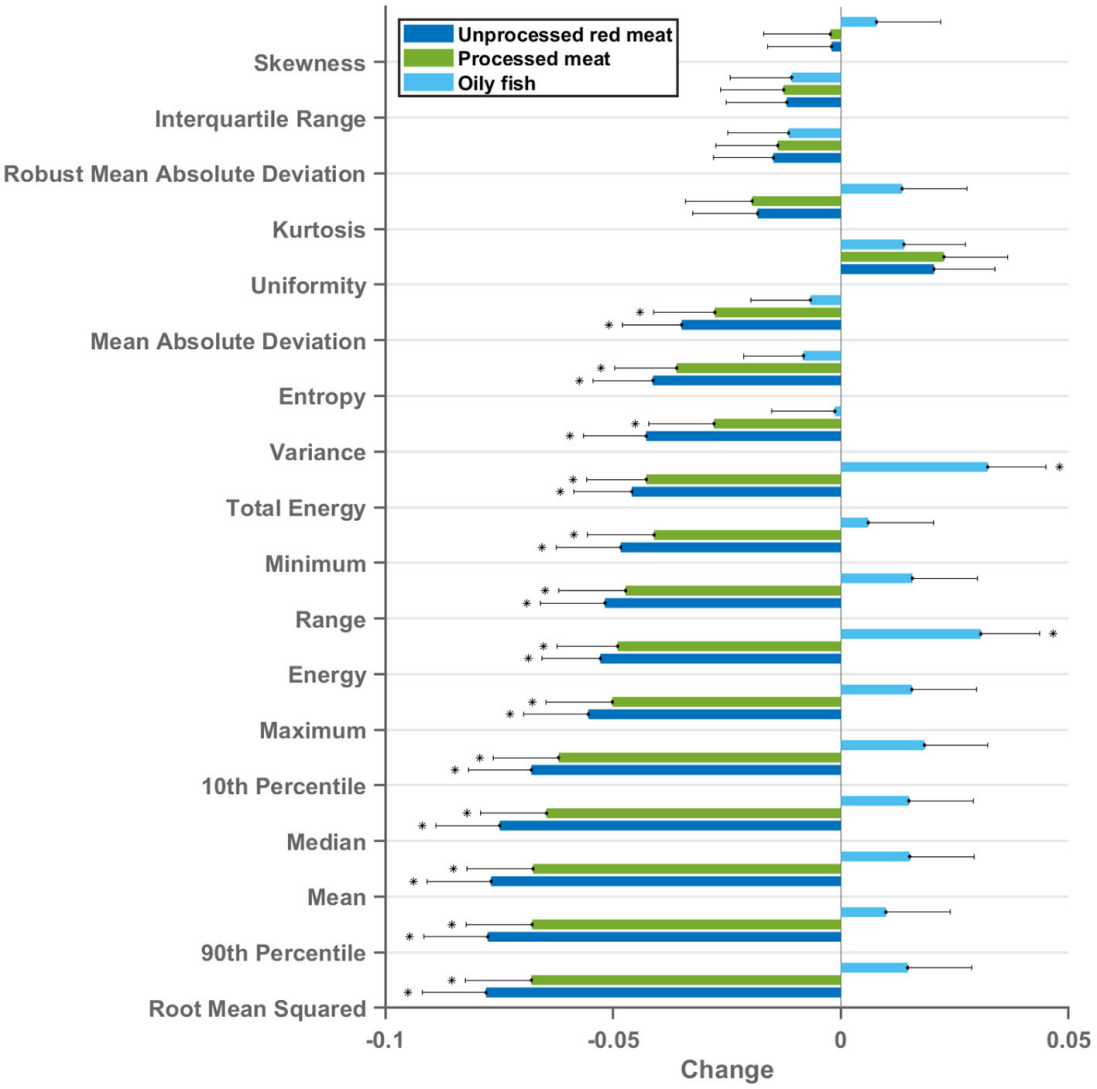
Multivariable linear regression models showing change in LV myocardium CMR first-order radiomics (end-diastole) per 100 g increase in daily meat consumption. Each bar represents standardised beta coefficients corresponding to the indicated radiomics first-order feature. Each bar is from a separate model adjusted for age, sex, social deprivation, educational level, smoking, alcohol intake, and exercise level. Black lines represent half-length of confidence interval for the corresponding bar. Bonferroni adjusted significance threshold *p* = 0.0009 (corrected for 54 comparisons). CMR, cardiovascular magnetic resonance; LV, left ventricle.

**Figure 5:**
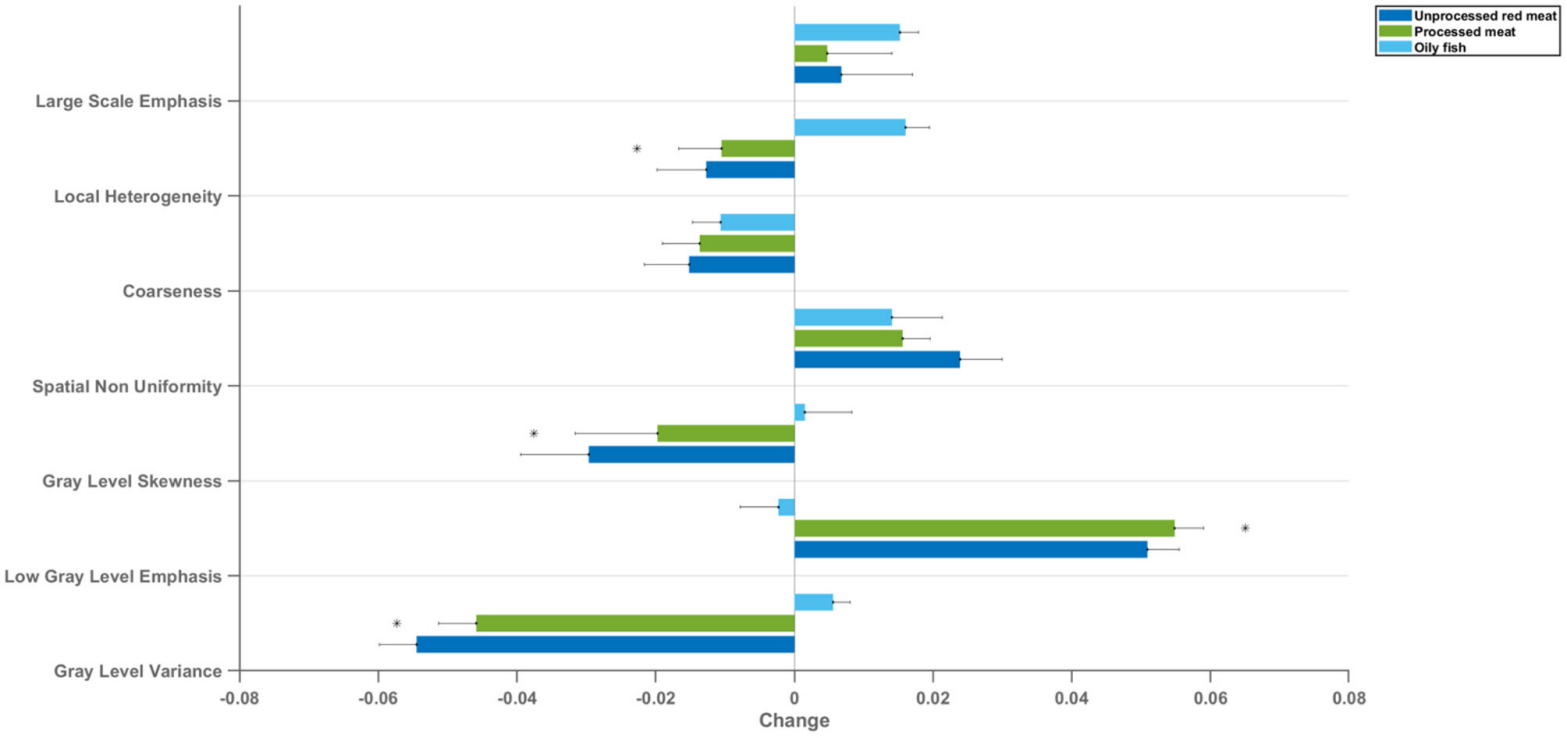
Mean change in LV myocardium CMR radiomics texture feature clusters per 100 g increase in daily meat consumption. Each bar represents mean standardised beta coefficients corresponding to the indicated texture feature cluster. Models are adjusted for age, sex, social deprivation, educational level, smoking, alcohol intake, and exercise level (confounder adjusted model). Black lines represent half-length of confidence interval for the corresponding bar. CMR: cardiovascular magnetic resonance; LV: left ventricle *denotes *p* < 0.05 in using Kruskal–Wallis statistical testing followed by Dunn's correction test for multiple comparisons between oily fish and unprocessed red meat and between oily fish and processed red meat.

First-order features are histogram-based statistics describing the global distribution of signal intensity values in the defined region and may signify global tissue-level myocardial changes (20). Eighteen radiomics first-order features were extracted from the LV myocardium in end-diastole and end-systole. The red/processed meat and fish exposures showed markedly different, often reverse, associations with radiomics first-order features (Figures 4, 5). Greater red and processed meat consumption was associated with lower average intensity levels and less variation in signal intensity values (consistent across all relevant metrics, such as, lower mean, median, range, variance, entropy). The reverse of these trends was observed with greater oily fish consumption: higher average signal intensity level, greater range of intensity levels, higher number of extreme intensities (kurtosis), and greater randomness of intensity values (entropy). These associations appeared consistent across different meat types and in end-diastole and end-systole (Supplementary Figure 3). Thus, associations with the global pattern of signal intensities in the LV myocardium are very different between the meat and fish exposures. These findings suggest that these exposures may be associated with different (reverse) global pattern of alterations at the myocardial level. Results from individual associations between meat and fish exposures and LV myocardium first-order features in end-diastole and end-systole are presented in Supplementary Tables 9, 10.

### Association of Meat and Fish Intake With LV Myocardium Radiomics Texture Features

**Figure 6:**
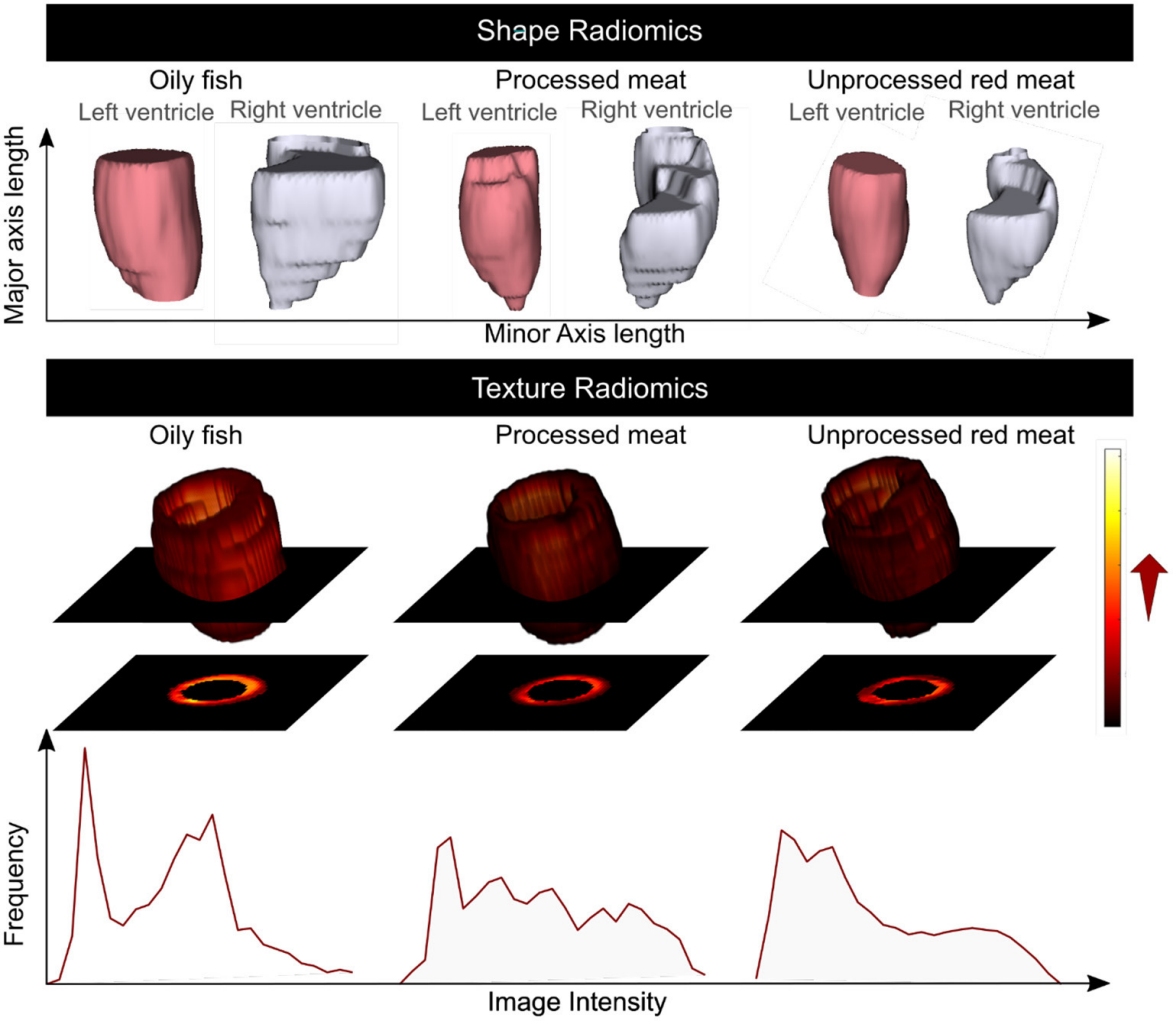
Summary of the association of the oily fish, processed meat, and unprocessed red meat intake with the CMR radiomics shape and signal intensity-based features. Greater red and processed meat intake was associated with smaller ventricular volumes, reduced short axis dimension, and a more elongated shape; lower global signal intensity levels, and less variation in SI levels within the LV myocardium. Greater oily fish consumption was associated with larger ventricles with overall less elongated (more spherical) shape, higher global myocardial intensity levels and more variation of myocardial intensities. CMR: cardiovascular magnetic resonance. Histograms are from a selection of most illustrative cases and do not represent findings from the whole dataset.

Radiomics texture features allow quantification of the pattern of inter-voxel signal intensities. Applied to the LV myocardium, radiomics texture features may provide biologically informative quantifiers about underlying tissue properties. We extracted 72 texture features from the LV myocardium in end-diastole and end-systole (total 144 features per CMR study). Cluster analysis identified seven inter-correlated groups of features (Figure 1), to which we assigned descriptive terms based on the features within the cluster (Table 3). Comparison of mean effects in these clusters showed different effect sizes and directions of effect across the various meat exposures (Figure 5). Greater red meat consumption was associated with lower intensity levels, lower variation in intensity levels, less local heterogeneity, and less skewness in the distribution of signal intensity values (Figures 5, 6). Greater oily fish consumption associated with greater local heterogeneity and greater skewness in the intensity level distribution. The pattern of associations of the meat and fish exposures with inter-voxel relationships was also different, suggesting distinct alterations at the myocardium. Results from individual associations between meat and fish exposures and individual LV myocardium texture features in end-diastole and end-systole are presented in Supplementary Tables 11, 12.

**Table 3:** Description of clusters identified from the radiomics texture features.

**Assigned cluster name**	**Exemplar feature from the cluster**	**Properties represented by cluster**
Low grey level emphasis	Low grey level emphasis	Local distribution and clustering of low SI values
Spatial non-uniformity	Size zone non-uniformity	Non-uniformity in the size of pixel groupings
Grey level variance	Grey level variance	Distribution of SI values
Coarseness	Run percentage	Tendency to small groupings of pixels with similar SI values
Local heterogeneity	Dependence entropy	Randomness of neighbouring pixel SI values
Large scale emphasis	Large area emphasis	Larger areas of similar pixel SI values
Grey level skewness	Cluster prominence	Skewness of the SI distribution

*The table summarises the seven distinct groups of radiomics texture features identified through cluster analysis of these features (n = 144, Figure 1). Each cluster incorporates a number of inter-correlated features. For each cluster, we provide an assigned name, an exemplar feature, and a general description of the properties represented. SI, signal intensity*.

### Association of Meat and Fish Intake With Arterial Compliance Measures

**Figure 7:**
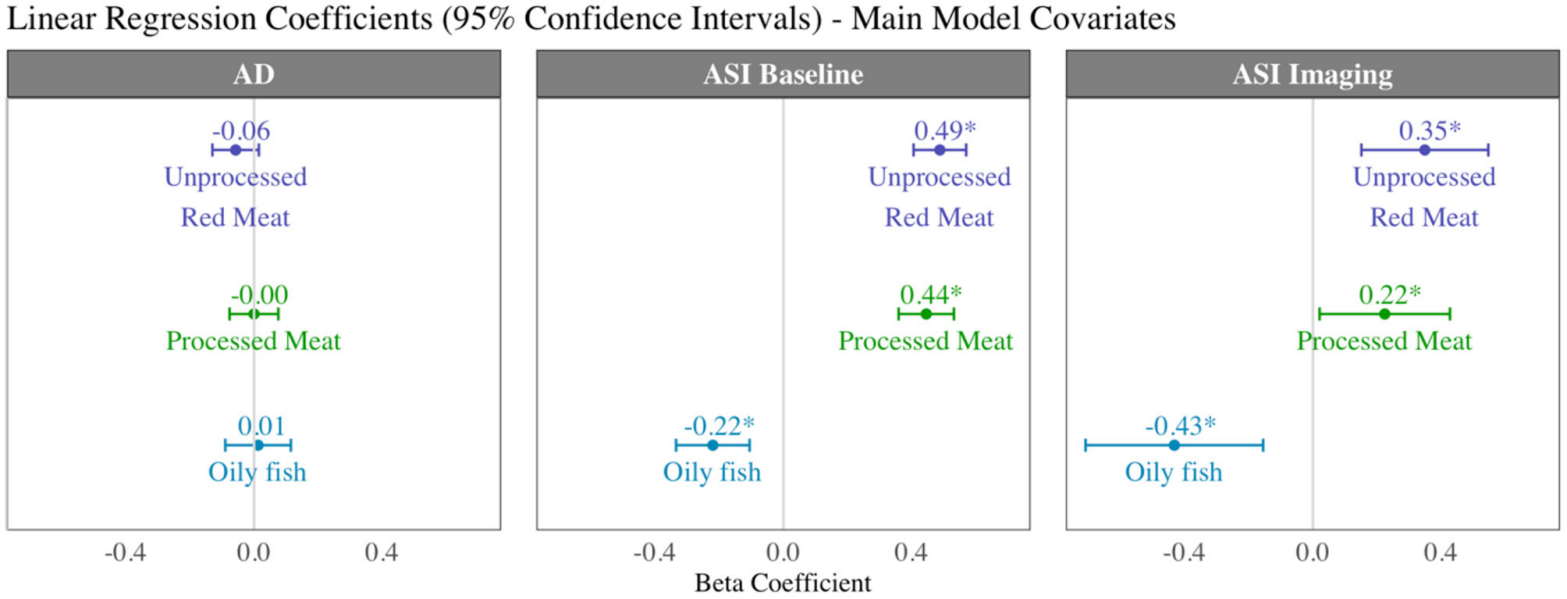
Summary of multivariable linear regression results for arterial compliance measures displaying beta coefficients and 95% confidence intervals per 100 g increase in daily intake of meat/fish. Each bar is from a separate model adjusted for age, sex, social deprivation, educational level, smoking, alcohol intake, and exercise level (confounder adjusted model). AD, aortic distensibility; ASI, arterial stiffness index.

There was record of ASI at the baseline, imaging, and at both time points for 167,432 (baseline characteristics: Supplementary Table 13), 30,474, and 10,436 participants, respectively. For the latter group, we considered interval “change in ASI”. Higher intake of red and processed meat was associated with higher ASI, indicating greater vascular resistance, at both the baseline and imaging visits (Table 4, Figure 7). In addition, higher unprocessed red meat intake was associated with significantly greater interval increase in ASI from baseline to imaging (Table 4). In contrast, greater oily fish consumption was associated with lower ASI at both time points and with a smaller baseline to imaging interval increase in ASI (not statistically significant).

**Table 4:** Multivariate linear regression models showing change in arterial compliance measures per 100 g increase in daily meat/fish consumption.

	**Aortic distensibility** ** (× 10^**−3**^ mmHg^**−1**^)**	**ASI** ** (baseline, m/s)**	**ASI** ** (imaging, m/s)**	**Interval change in ASI** ** (baseline-imaging, m/s)**
Unprocessed red meat	−0.06 [−0.13, 0.02] 0.12	0.49^*^ [0.41, 0.57] 2.26 × 10^−31^	0.349^*^ [0.15, 0.55] 5.46 × 10^−4^	0.150^*^ [0.03, 0.27] 0.02
Processed meat	−0.00 [−0.08, 0.08] 1.00	0.45^*^ [0.36, 0.53] 4.47 × 10^−24^	0.22^*^ [0.02, 0.43] 0.03	0.05 [−0.07, 0.17] 0.43
Oily fish	0.01 [−0.09, 0.12] 0.81	−0.22^*^ [−0.34, −0.11] 1.70 × 10^−4^	−0.43^*^ [−0.71, −0.16] 2.20 × 10^−3^	−0.17 [−0.34, 0.01] 0.06

*Each cell represents a separate model, adjusted for: age, sex, social deprivation, educational level, smoking, alcohol intake, and exercise level. For “interval change in ASI,” results are average standard deviation change from that expected from baseline. Results are presented as beta coefficient [95% confidence interval]; p-value. ASI, arterial stiffness index*.

* *p < 0.05*.

Greater red and processed meat consumption was associated with lower aortic distensibility and greater oily fish consumption with higher aortic distensibility (not statistically significant, Table 4). Relationships with all arterial compliance outcomes were consistent across the three red meat groups (Supplementary Table 14) and broadly unchanged with adjustment for potential covariates on the causal pathway (Supplementary Table 15).

## Discussion

### Summary of Findings

In this study of 9,303 men and 10,105 women, greater red and processed meat consumption was associated with impaired cardiovascular health, both in terms of markers of arterial disease and of cardiac structure and function. In contrast, greater oily fish intake was linked with a healthy cardiovascular phenotype.

Specifically, greater red and processed meat intake was associated with smaller ventricular volumes, poorer myocardial function (lower LV/RV stroke volume), and poorer arterial compliance (higher ASI, greater interval increases in ASI, lower aortic distensibility). By comparison, greater oily fish consumption was associated with larger LV and RV volumes, better myocardial function (higher LV/RV stroke volume), and better arterial health (lower ASI, smaller interval increases in ASI, higher aortic distensibility). There was evidence that cardiometabolic morbidities may have a mechanistic role in the associations of unprocessed red meat with ventricular phenotypes, but less so for other associations suggesting importance of alternative mechanisms for these relationships. Radiomics analysis provided complementary and incremental information demonstrating association of the different dietary habits with distinct overall shape of the ventricles and LV myocardial texture. Greater oily fish consumption was associated with a less elongated LV (more spherical), whilst greater red and processed meat intake was associated with a more elongated LV. The different dietary habits were also associated with different patterns of associations with signal intensity based radiomics features (first-order, texture). Overall, greater red and processed meat intake was associated with lower average global signal intensity and a more homogenous signal intensity pattern both globally and when considering inter-pixel relationships. In contrast, greater oily fish consumption was associated with, on average, a brighter myocardium (global higher signal intensity), with greater range and variation in signal intensities, and greater randomness in the pattern of intensity levels. These findings indicate that meat and fish consumption are associated with different signal intensity patterns at the LV myocardium, suggesting possible differences at the tissue level associated with the different exposures.

### Comparison With Existing Literature

To the best of our knowledge, the specific impact of red or processed meat intake on CMR imaging phenotypes has not been previously studied. The association between a number of dietary patterns and CMR indices of cardiac structure and function have previously been addressed in two studies of the Multi-Ethnic Study of Atherosclerosis (MESA) cohort. These evaluated long-term effect on CMR measures of LV structure and function of two specific dietary patterns: Mediterranean (35) and the Dietary Approaches to Stop Hypertension (DASH) (36) diets, both of which associated with healthy cardiovascular phenotypes (larger cavity volumes, higher LV mass, higher stroke volume, higher ejection fraction). Limited further studies have focused on diet and cardiovascular structure assessed by echocardiography. Maugeri et al. (37) reported higher rates of concentric left ventricular hypertrophy in individuals following a “western” dietary pattern. Similarly, Wagner et al. (38) documented associations between unhealthy dietary behaviours and higher LV mass. Haring et al. (5) report association of higher red and processed meat intake with poorer imaging indicators of arterial health (greater intima medial thickness and atherosclerotic burden on carotid ultrasound). Our findings corroborate existing evidence and contribute incremental knowledge by demonstrating detailed cardiac phenotypic indices associated with red/processed meat and oily fish consumption in the largest population to date, using both conventional and novel radiomics CMR measures and measures of vascular compliance.

Existing literature suggests a number of possible explanations for the association between higher red meat consumption and cardiovascular disease. Firstly, these observed effects may be mediated through alterations of the cardiometabolic profile. Greater red and processed meat intake is linked to adverse lipid profiles (10), higher blood pressure (9), adverse body composition (39), and reduced insulin sensitivity (40). Interestingly, in our study, the observed effects on cardiovascular structure and function were not fully explained with adjustment for potential cardiometabolic mediators, suggesting a role for mechanistic pathways independent of these morbidities. Alternative disease pathways such as the gut microbiome dependent trimethylamine N-oxide (TMAO) pathway may play a role in this association: red meat intake, rich in carnitine, is known to increase both plasma and urine TMAO levels, by increased provision of the precursor, L-carnitine, and reduced fractional renal TMOA excretion (41). TMAO has, in turn, been mechanistically associated with atherosclerotic disease (11). In our study, associations with arterial health were largely unchanged with additional adjustment for cardiometabolic mediators, suggesting that alternative pathways, such as the TMAO pathway, may be more important in mediating associations with arterial disease, whereas cardiometabolic factors are more important in driving relationships with cardiac health. The TMAO pathway may thus present potential novel therapeutic targets for targeting arterial disease.

### Strengths and Limitations

The large sample and detailed characterisation of participants including CMR scanning and objective measures of arterial health permitted a uniquely comprehensive assessment of the relationship between the various meat exposures and cardiovascular phenotypes with consideration of a range of confounders and mediators. The uniform scanning and analysis procedures presented a high-quality standardised dataset. Common to all nutritional epidemiology research, the measurement and tracking of dietary behaviours is extremely difficult. Our exposures are defined on the basis of a self-report food frequency questionnaire from a single time point, and thus do not account for potential changes in dietary behaviour over time. However, a formal evaluation of the performance of the UKB dietary questionnaire demonstrated good repeatability for the main food groups (42). Furthermore, as potential measurement error is likely to be non-differential across the spectrum of meat consumption and meat types, the risk of bias implied by this is low. The UK Biobank dietary questionnaire gathers information on dietary habits over the preceding 12 months. It is possible that duration of exposure to various healthy or unhealthy dietary habits may modify the observed relationships. However, the available data does not permit such evaluations in the current analysis. We were unable to consider more granular details regarding covariates (e.g., hypertension) which may have important disease modifying effects, for example, we are unable to distinguish individuals with poorly controlled disease or those with evidence of end-organ damage. It is also possible that certain medications have a modifying effect on associations with cardiovascular phenotypes. Information on medication in the UK Biobank is recorded at baseline based on self-report, the completeness and accuracy of this data cannot be verified against clinical records, nor can links be definitively made to specific conditions. As such, we have not taken into account potential effect of medications on the observed relationships. These would be important considerations in future work. Furthermore, as the UK Biobank participants in this anlaysis are predominantly of White ethnic background (97%), we cannot be certain that observed associations are generalisable across different ethnicities. With regards the radiomics analysis, the reproducibility of these features is highly susceptible to variations in image segmentation. This is a major challenge with radiomics analysis, particularly when the goal is to develop generalisable clinical models. In the present study, we use radiomics for characterising associations with deeper cardiac phenotypes, as the goal is not to produce a clinical model for application to external datasets, the reproducibility issues are less relevant here. The potential effect of poor reproducibility in the present study would be to introduce noise into radiomics features with possible attenuation of some association. Another limitation is that the relatively novel approach of radiomics, although providing unique information, is difficult to interpret and so any conclusions will be descriptive and rather speculative at this stage. Finally, due to the observational nature of the study, we are unable to exclude residual confounding or infer causality.

## Conclusion

Greater consumption of red and processed meat is associated with poorer cardiovascular health characterised in terms of CMR cardiac structure and function, novel radiomics features, and measures of arterial compliance from CMR and plethysmography. Our findings support previous clinical associations and provide greater insight into potential mechanisms of dietary impact on cardiovascular health.

## Data Availability Statement

The data analysed in this study is subject to the following licences/restrictions: This research was conducted using the UKB resource under access application 2964. UK Biobank will make the data available to all bona fide researchers for all types of health-related research that is in the public interest, without preferential or exclusive access for any persons. All researchers will be subject to the same application process and approval criteria as specified by UK Biobank. For more details on the access procedure, see the UK Biobank website. Requests to access these datasets should be directed to http://www.ukbiobank.ac.uk/register-apply/.

## Ethics Statement

The studies involving human participants were reviewed and approved by NHS National Research Ethics Service (Ref 11/NW/0382). The patients/participants provided their written informed consent to participate in this study.

## Author Contributions

ZR-E, SEP, and NCH conceived the idea and designed the study. CM performed statistical analysis. JC cross-checked and advised on statistical analysis. PG led the radiomics analysis. AJ contributed to the radiomics analysis. KL supervised radiomics analysis. LB provided aortic distensibility measures from automated analysis pipeline. NA provided cardiac measures from automated analysis pipeline. ZR-E wrote the manuscript. All authors read and provided critical appraisal of the manuscript.

## Conflict of Interest

The authors declare that the research was conducted in the absence of any commercial or financial relationships that could be construed as a potential conflict of interest.

## Abbreviations

ASI: arterial stiffness index
AD: aortic distensibility
BMI: body mass index
CI: confidence interval
CMR: cardiovascular magnetic resonance
LV: left ventricle
RV: right ventricle
NHS: national health service
ROI: region of interest
SI: signal intensity
TMAO: trimethylamine N-oxide.

## References

[1] Bovalino S Charleson G Szoeke C. The impact of red and processed meat consumption on cardiovascular disease risk in women. Nutrition. (2016) 32:349–54. 10.1016/j.nut.2015.09.015 26732834

[2] Sinha R Cross AJ Graubard BI Leitzmann MF Schatzkin A. Meat intake and mortality a prospective study of over half a million people. Arch Intern Med. (2009) 169:562–71. 10.1001/archinternmed.2009.6 19307518 PMC2803089

[3] Kontogianni MD Panagiotakos DB Pitsavos C Chrysohoou C Stefanadis C. Relationship between meat intake and the development of acute coronary syndromes: the CARDIO2000 case - control study. Eur J Clin Nutr. (2008) 62:171–7. 10.1038/sj.ejcn.1602713 17356558

[4] Bernstein AM Sun Q Hu FB Stampfer MJ Manson JE Willett WC. Major dietary protein sources and risk of coronary heart disease in women. Circulation. (2010) 122:876–83. 10.1161/CIRCULATIONAHA.109.915165 20713902 PMC2946797

[5] Haring B Wang W Fretts A Shimbo D Lee ET Howard BV. Red meat consumption and cardiovascular target organ damage (from the strong heart study). J Hypertens. (2017) 35:1794–800. 10.1097/HJH.0000000000001385 28399044 PMC5728368

[6] Zhong VW Van Horn L Greenland P Carnethon MR Ning H Wilkins JT. Associations of Processed Meat, Unprocessed Red Meat, Poultry, or Fish Intake with Incident Cardiovascular Disease and All-Cause Mortality. JAMA Intern Med. (2020) 180:503–512. 10.1001/jamainternmed.2019.6969 32011623 PMC7042891

[7] Wolk A. Potential health hazards of eating red meat. J Intern Med. (2017) 281:106–122. 10.1111/joim.12543 27597529

[8] Organ CL Otsuka H Bhushan S Wang Z Bradley J Trivedi R. Choline diet and its gut microbe-derived metabolite, trimethylamine n-oxide, exacerbate pressure overload-induced heart failure. Circ Hear Fail. (2016) 9:e002314. 10.1161/CIRCHEARTFAILURE.115.002314 26699388 PMC4943035

[9] Steffen LM Kroenke CH Yu X Pereira MA Slattery ML Van Horn L. Associations of plant food, dairy product, and meat intakes with 15-y incidence of elevated blood pressure in young black and white adults: the coronary artery risk development in young adults (CARDIA) study. Am J Clin Nutr. (2005) 82:1169–77. 10.1093/ajcn/82.6.1169 16332648

[10] Wolmarans P Benadé AJ Kotze TJ Daubitzer AK Marais MP Laubscher R. Plasma lipoprotein response to substituting fish for red meat in the diet. Am J Clin Nutr. (1991) 53:1171–6. 10.1093/ajcn/53.5.1171 2021128

[11] Koeth RA Wang Z Levison BS Buffa JA Org E Sheehy BT. Intestinal microbiota metabolism of l-carnitine, a nutrient in red meat, promotes atherosclerosis. Nat Med. (2013) 19:576–85. 10.1038/nm.3145 23563705 PMC3650111

[12] Raisi-Estabragh Z Petersen SE. Cardiovascular research highlights from the UK Biobank: opportunities and challenges. Cardiovasc Res. (2020) 116:e12–5. 10.1093/cvr/cvz294 31778147

[13] UK Biobank. Protocol for a Large-Scale Prospective Epidemiological Resource. (2007). Available online at: https://www.ukbiobank.ac.uk/wp-content/uploads/2011/11/UK-Biobank-Protocol.pdf (accessed December 13, 2019).

[14] Raisi-Estabragh Z Harvey NC Neubauer S Petersen SE. Cardiovascular magnetic resonance imaging in the UK Biobank: a major international health research resource. Eur Hear J Cardiovasc Imaging. (2020) 22:jea297. 10.1093/ehjci/jeaa297 33164079 PMC7899275

[15] Schenker S. Portion sizes Food Fact Sheet. (2016). Available online at: www.bda.uk.com/foodfacts (accessed April 25, 2020).

[16] Anderson JJ Darwis NDM Mackay DF Celis-Morales CA Lyall DM Sattar N. Red and processed meat consumption and breast cancer: UK Biobank cohort study and meta-analysis. Eur J Cancer. (2018) 90:73–82. 10.1016/j.ejca.2017.11.022 29274927

[17] Petersen SE Matthews PM Francis JM Robson MD Zemrak F Boubertakh R. UK Biobank's cardiovascular magnetic resonance protocol. J Cardiovasc Magn Reson. (2015) 18:8. 10.1186/s12968-016-0227-4 26830817 PMC4736703

[18] Petersen SE Aung N Sanghvi MM Zemrak F Fung K Paiva JM. Reference ranges for cardiac structure and function using cardiovascular magnetic resonance (CMR) in caucasians from the UK Biobank population cohort. J Cardiovasc Magn Reson. (2017) 19:18. 10.1186/s12968-017-0327-9 28178995 PMC5304550

[19] Attar R Pereañez M Gooya A Albà X Zhang L de Vila MH. Quantitative CMR population imaging on 20,000 subjects of the UK Biobank imaging study: LV/RV quantification pipeline and its evaluation. Med Image Anal. (2019) 56:26–42. 10.1016/j.media.2019.05.006 31154149

[20] Raisi-Estabragh Z Izquierdo C Campello VM Martin-isla C Jaggi A Harvey NC. Cardiac magnetic resonance radiomics: basic principles and clinical perspectives. Eur Heart J Cardiovasc Imaging. (2020) 21:349–356. 10.1093/ehjci/jeaa028 32142107 PMC7082724

[21] Van Griethuysen JJM Fedorov A Parmar C Hosny A Aucoin N Narayan V. Computational radiomics system to decode the radiographic phenotype. Cancer Res. (2017) 77:e104–7. 10.1158/0008-5472.CAN-17-0339 29092951 PMC5672828

[22] Gonzalez R Fittes B. 2nd conference on remotely manned systems: technology and applications. In: Gray-Level Transformations for Interactive Image Enhancement. Los Angeles, CA (2020). p. 17–19. Available online at: https://ntrs.nasa.gov/archive/nasa/casi.ntrs.nasa.gov/19770022806.pdf

[23] Laurent S Cockcroft J Van Bortel L Boutouyrie P Giannattasio C Hayoz D. Expert consensus document on arterial stiffness: methodological issues and clinical applications. Eur Heart J. (2006) 27:2588–605. 10.1093/eurheartj/ehl254 17000623

[24] Resnick LM Militianu D Cunnings AJ Pipe JG Evelhoch JL Soulen RL. Direct magnetic resonance determination of aortic distensibility in essential hypertension. Hypertension. (1997) 30:654–9. 10.1161/01.HYP.30.3.654 9322999

[25] Biasiolli L Hann E Lukaschuk E Carapella V Paiva JM Aung N. Automated localization and quality control of the aorta in cine CMR can significantly accelerate processing of the UK Biobank population data. PLoS ONE. (2019) 14:e0212272. 10.1371/journal.pone.0212272 30763349 PMC6375606

[26] Abdullah Said M Eppinga RN Lipsic E Verweij N van der Harst P. Relationship of arterial stiffness index and pulse pressure with cardiovascular disease and mortality. J Am Heart Assoc. (2018) 7:e007621. 10.1161/JAHA.117.007621 29358193 PMC5850166

[27] UK Biobank Arterial Pulse-Wave Velocity. (2011). Available online at: https://biobank.ndph.ox.ac.uk/showcase/showcase/docs/Pulsewave.pdf (accessed December 4, 2019).

[28] R Core Team. R: A Language and Environment for Statistical Computing. Vienna: R Foundation for Statistical Computing (2019). Available online at: https://www.r-project.org/ (accessed October 18, 2020).

[29] RStudio. Integrated Development for R. Boston, MA: RStudio, Inc. Available online at: https://rstudio.com/ (accessed October 18, 2020).

[30] Maechler M. “Finding Groups in Data”: Cluster Analysis Extended Rousseeuw et al. R Packag. version 2.0. (2019). Available online at: https://www.rdocumentation.org/packages/cluster/versions/2.1.0 (accessed May 3, 2020).

[31] Townsend P Phillimore P Beattie A. Health and deprivation: inequality and the North. Nurs Stand. (1988) 2:34. 10.7748/ns.2.17.34.s66 27415096

[32] Craig CL Marshall AL Sjöström M Bauman AE Booth ML Ainsworth BE. International physical activity questionnaire: 12-country reliability and validity. Med Sci Sports Exerc. (2003) 35:1381–95. 10.1249/01.MSS.0000078924.61453.FB 12900694

[33] Batty GD Gale CR Kivimäki M Deary IJ Bell S. Comparison of risk factor associations in UK Biobank against representative, general population based studies with conventional response rates: prospective cohort study and individual participant meta-analysis. BMJ. (2020) 368:m131. 10.1136/bmj.m131 32051121 PMC7190071

[34] Fry A Littlejohns TJ Sudlow C Doherty N Adamska L Sprosen T. Comparison of sociodemographic and health-related characteristics of UK biobank participants with those of the general population. Am J Epidemiol. (2017) 186:1026–34. 10.1093/aje/kwx246 28641372 PMC5860371

[35] Levitan EB Ahmed A Arnett DK Polak JF Hundley WG Bluemke DA. Mediterranean diet score and left ventricular structure and function: the multi-ethnic study of atherosclerosis. Am J Clin Nutr. (2016) 104:595–602. 10.3945/ajcn.115.128579 27488238 PMC4997295

[36] Nguyen HT Bertoni AG Nettleton JA Bluemke DA Levitan EB Burke GL. Dash eating pattern is associated with favorable left ventricular function in the multi-ethnic study of atherosclerosis. J Am Coll Nutr. (2012) 31:401–7. 10.1080/07315724.2012.10720466 23756584 PMC4119794

[37] Maugeri A Hruskova J Jakubik J Hlinomaz O Medina-Inojosa JR Barchitta M. How dietary patterns affect left ventricular structure, function and remodelling: evidence from the kardiovize brno 2030 study. Sci Rep. (2019) 9:19154. 10.1038/s41598-019-55529-5 31844105 PMC6915714

[38] Wagner S Lioret S Girerd N Duarte K Lamiral Z Bozec E. Association of dietary patterns derived using reduced-rank regression with subclinical cardiovascular damage according to generation and sex in the STANISLAS cohort. J Am Heart Assoc. (2020) 9:e013836. 10.1161/JAHA.119.013836 32200718 PMC7428593

[39] van Baak MA Larsen TM Jebb SA Martinez A Saris WHM Handjieva-Darlenska T. Dietary intake of protein from different sources and weight regain, changes in body composition and cardiometabolic risk factors afterweight loss: The DIOgenes study. Nutrients. (2017) 9:1326. 10.3390/nu9121326 29211027 PMC5748776

[40] Shang X Scott D Hodge AM English DR Giles GG Ebeling PR. Dietary protein intake and risk of type 2 diabetes: results from the Melbourne collaborative cohort study and a meta-analysis of prospective studies. Am J Clin Nutr. (2016) 104:1352–65. 10.3945/ajcn.116.140954 27629053

[41] Wang Z Bergeron N Levison BS Li XS Chiu S Jia X. Impact of chronic dietary red meat, white meat, or non-meat protein on trimethylamine N-oxide metabolism and renal excretion in healthy men and women. Eur Heart J. (2019) 40:583–94. 10.1093/eurheartj/ehy799 30535398 PMC6374688

[42] Bradbury KE Young HJ Guo W Key TJ. Dietary assessment in UK Biobank: an evaluation of the performance of the touchscreen dietary questionnaire. J Nutr Sci. (2018) 7:e6. 10.1017/jns.2017.66 29430297 PMC5799609

