# Supplementary material for: Associations of Meat and Fish Consumption With Conventional and Radiomics Cardiovascular Magnetic Resonance Phenotypes in the UK Biobank
Source: Front Cardiovasc Med. 2021 May 5;8:667849. doi: 10.3389/fcvm.2021.667849 (PMC8133433; doi:10.3389/fcvm.2021.667849)
Supplement: Supplementary file 1 [file Data_Sheet_1.docx]

**Supplementary table 1. Selected components of the touchscreen food intake questionnaire**

| Meat type | Touchscreen question | Help message* |
| --- | --- | --- |
| Lamb | How often do you eat lamb/mutton? (Do not count processed meats) | Please provide an average considering your intake over the last year.  If you are unsure, please provide an estimate or select Do not know. |
| Beef | How often do you eat beef? (Do not count processed meats) | Please provide an average considering your intake over the last year.  If you are unsure, please provide an estimate or select Do not know. |
| Pork | How often do you eat pork? (Do not count processed meats such as bacon or ham) | Please provide an average considering your intake over the last year.  If you are unsure, please provide an estimate or select Do not know. |
| Processed meat | How often do you eat processed meats (such as bacon, ham, sausages, meat pies, kebabs, burgers, chicken nuggets)? | Please provide an average considering your intake over the last year.  If you are unsure, please provide an estimate or select Do not know. |
| Oily fish | How often do you eat oily fish? (e.g., sardines, salmon, mackerel, herring) | Please provide an average considering your intake over the last year.  If you are unsure, please provide an estimate or select Do not know.  Oily fish include: Salmon Anchovies, Trout Swordfish, Mackerel Bloater, Herring Cacha, Sardines Carp, Pilchards Hilsa, Kipper Jack fish, Eel Katla, Whitebait Orange roughy, Tuna (fresh only) Pangas, Sprats |

**Supplementary table 1 footnote: ***The help message was displayed if the participant activated the help button. For all questions the following answers were possible: never, less than once a week, once a week, 2-4 times a day, 5-6 times a week, once or more daily, do not know, prefer not to answer

**Supplementary Table 2. List of CMR radiomics features extracted and included in the analysis grouped by feature category**

| **Shape** | **First-order** | **Texture** |
| --- | --- | --- |
| Volume | Energy | Autocorrelation (GLCM) |
| Surface Area | Total Energy | Joint Average (GLCM) |
| Surface Area To Volume Ratio | Entropy | Cluster Prominence (GLCM) |
| Sphericity | Minimum | Cluster Shade (GLCM) |
| Maximum 3D Diameter | 10th Percentile | Cluster Tendency (GLCM) |
| Maximum 2D Diameter (Slice) | 90th Percentile | Contrast (GLCM) |
| Maximum 2D Diameter (Column) | Maximum | Correlation (GLCM) |
| Maximum 2D Diameter (Row) | Mean | Difference Average (GLCM) |
| Major Axis Length | Median | Difference Entropy (GLCM) |
| Minor Axis Length | Interquartile Range | Difference Variance (GLCM) |
| Least Axis Length | Range | Joint Energy (GLCM) |
| Elongation | Mean Absolute Deviation | Joint Entropy (GLCM) |
| Flatness | Robust Mean Absolute Deviation | Informal Measure Of Correlation 1 (GLCM) |
|  | Root Mean Squared | Informal Measure Of Correlation 2 (GLCM) |
|  | Skewness | Inverse Difference Moment (GLCM) |
|  | Kurtosis | Inverse Difference Moment Normalized (GLCM) |
|  | Variance | Inverse Difference (GLCM) |
|  | Uniformity | Inverse Difference Normalized (GLCM) |
|  |  | Inverse Variance (GLCM) |
|  |  | Maximum Probability (GLCM) |
|  |  | Sum Average (GLCM) |
|  |  | Sum Entropy (GLCM) |
|  |  | Sum Of Squares (GLCM) |
|  |  | Small Area Emphasis (GLSZM) |
|  |  | Large Area Emphasis (GLSZM) |
|  |  | Grey Level Non Uniformity (GLSZM) |
|  |  | Size Zone Non Uniformity (GLSZM) |
|  |  | Size Zone Non Uniformity Normalized (GLSZM) |
|  |  | Zone Percentage (GLSZM) |
|  |  | Grey Level Variance (GLSZM) |
|  |  | Zone Variance (GLSZM) |
|  |  | Zone Entropy (GLSZM) |
|  |  | Low Grey Level Zone Emphasis (GLSZM) |
|  |  | High Grey Level Zone Emphasis (GLSZM) |
|  |  | Small Area Low Grey Level Emphasis (GLSZM) |
|  |  | Small Area High Grey Level Emphasis (GLSZM) |
|  |  | Large Area Low Grey Level Emphasis (GLSZM) |
|  |  | Large Area High Grey Level Emphasis (GLSZM) |
|  |  | Short Run Emphasis (GLRLM) |
|  |  | Long Run Emphasis (GLRLM) |
|  |  | Grey Level Non Uniformity (GLRLM) |
|  |  | Run Length Non Uniformity (GLRLM) |
|  |  | Run Length Non Uniformity Normalized (GLRLM) |
|  |  | Run Percentage (GLRLM) |
|  |  | Grey Level Variance (GLRLM) |
|  |  | Run Variance (GLRLM) |
|  |  | Run Entropy (GLRLM) |
|  |  | Low Grey Level Run Emphasis (GLRLM) |
|  |  | High Grey Level Run Emphasis (GLRLM) |
|  |  | Short Run Low Grey Level Emphasis (GLRLM) |
|  |  | Short Run High Grey Level Emphasis (GLRLM) |
|  |  | Long Run Low Grey Level Emphasis (GLRLM) |
|  |  | Long Run High Grey Level Emphasis (GLRLM) |
|  |  | Coarseness (NGTDM) |
|  |  | Contrast (NGTDM) |
|  |  | Busyness (NGTDM) |
|  |  | Complexity (NGTDM) |
|  |  | Strength (NGTDM) |
|  |  | Small Dependence Emphasis (GLDM) |
|  |  | Large Dependence Emphasis (GLDM) |
|  |  | Grey Level Non Uniformity (GLDM) |
|  |  | Dependence Non Uniformity (GLDM) |
|  |  | Dependence Non Uniformity Normalized (GLDM) |
|  |  | Grey Level Variance (GLDM) |
|  |  | Dependence Variance (GLDM) |
|  |  | Dependence Entropy (GLDM) |
|  |  | Low Grey Level Emphasis (GLDM) |
|  |  | High Grey Level Emphasis (GLDM) |
|  |  | Small Dependence Low Grey Level Emphasis (GLDM) |
|  |  | Small Dependence High Grey Level Emphasis (GLDM) |
|  |  | Large Dependence Low Grey Level Emphasis (GLDM) |
|  |  | Large Dependence High Grey Level Emphasis (GLDM) |

**Supplementary Table 2 footnote:** CMR: cardiovascular magnetic resonance; GLCM: grey level co-occurrence matrix; GLDM: grey level dependence matrix; GLRLM: grey level run length matrix; GLSZM: grey level size zone matrix; NGTDM: neighboring grey tone difference matrix; LV: left ventricle.

**Supplementary Table 3. Multivariable linear regression models showing change in CMR indices per 100g increase in daily meat/fish consumption**

|  | LVEDVi (ml/m^2^) | LVESVi (ml/m^2^) | LVEF (%) | LVSVi (ml/m^2^) | LVMi (g/m^2^) | RVEDVi (ml/m^2^) | RVESVi (ml/m^2^) | RVSVi (ml/m^2^) | RVEF (%) |
| --- | --- | --- | --- | --- | --- | --- | --- | --- | --- |
| Unprocessed red meat | -2.183* | -0.962* | 0.041 | -1.221* | 1.570* | -1.767* | -0.607 | -1.16* | -0.304 |
|  | [-3.364, -1.002] | [-1.695, -0.229] | [-0.504, 0.587] | [-1.972, -0.469] | [0.910, 2.230] | [-3.298, -0.236] | [-1.541, 0.327] | [-2.111, -0.208] | [-0.973, 0.365] |
|  | 2.91$\times$10^-4^ | 0.0101 | 0.8817 | 0.0015 | 3.19$\times$10^-6^ | 0.0237 | 0.2026 | 0.0169 | 0.3726 |
| Beef | -3.047* | -1.245* | -0.011 | -1.803* | 2.126* | -3.773* | -2.139* | -1.634* | 0.498 |
|  | [-5.040, -1.055] | [-2.482, -0.007] | [-0.933, 0.911] | [-3.070, -0.535] | [1.012, 3.240] | [-6.329, -1.217] | [-3.698, -0.580] | [-3.223, -0.045] | [-0.619, 1.615] |
|  | 0.0027 | 0.0486 | 0.9811 | 0.0053 | 1.85$\times$10^-4^ | 0.0038 | 0.0072 | 0.0439 | 0.382 |
| Lamb | -5.591* | -2.673* | 0.149 | -2.918* | 2.272* | -1.415 | 1.371 | -2.786* | -2.532* |
|  | [-8.957, -2.224] | [-4.763, -0.582] | [-1.403, 1.701] | [-5.060, -0.776] | [0.389, 4.154] | [-5.864, 3.035] | [-1.343, 4.085] | [-5.552, -0.020] | [-4.477, -0.587] |
|  | 1.10$\times$10^-3^ | 0.0122 | 0.8509 | 0.00760 | 0.0180 | 0.533 | 0.3221 | 0.0484 | 0.0107 |
| Pork | -2.812 | -1.286 | 0.129 | -1.526 | 3.523* | -2.07 | -0.185 | -1.885 | -1.249 |
|  | [-5.836, 0.212] | [-3.162, 0.590] | [-1.271, 1.529] | [-3.450, 0.398] | [1.833, 5.214] | [-6.124, 1.983] | [-2.658, 2.287] | [-4.405, 0.635] | [-3.022, 0.523] |
|  | 0.0684 | 0.1792 | 0.857 | 0.1201 | 4.43$\times$10^-5^ | 0.3167 | 0.8833 | 0.1425 | 0.1671 |
| Processed meat | -2.884* | -1.118* | -0.121 | -1.765* | 0.568 | -2.884* | -0.835 | -2.049* | -0.566 |
|  | [-4.118, -1.649] | [-1.885, -0.352] | [-0.693, 0.451] | [-2.551, -0.980] | [-0.123, 1.259] | [-4.464, -1.305] | [-1.799, 0.129] | [-3.031, -1.068] | [-1.256, 0.125] |
|  | 4.70$\times$10^-6^ | 0.0042 | 0.6785 | 1.06$\times$10^-5^ | 0.1072 | 3.46$\times$10^-4^ | 0.0895 | 4.30$\times$10^-5^ | 0.1085 |
| Oily fish | 4.132* | 1.748* | 0.103 | 2.384* | 2.376* | 3.667* | 1.972* | 1.695* | -0.388 |
|  | [2.460, 5.804] | [0.710, 2.786] | [-0.671, 0.878] | [1.320, 3.448] | [1.441, 3.312] | [1.514, 5.821] | [0.659, 3.286] | [0.356, 3.034] | [-1.329, 0.553] |
|  | 1.28$\times$10^-6^ | 9.68$\times$10^-4^ | 0.7938 | 1.13$\times$10^-5^ | 6.40$\times$10^-7^ | 8.47$\times$10^-4^ | 0.0033 | 0.0131 | 0.4186 |

**Supplemental Table 3 footnote:** Each cell represents a separate model, adjusted for: age, sex, social deprivation, educational level, smoking, alcohol intake, exercise level (confounder adjusted model). First, second, and third row for every CMR measures corresponds to beta coefficient, 95% confidence interval and p-value, respectively. CMR: cardiovascular magnetic resonance; LVEDV: left ventricular end-diastolic volume; LVESV: left ventricular end-systolic volume; LVEF: left ventricular ejection fraction; LVM: left ventricular mass; LVSV: left ventricular stroke volume; RVEDV: right ventricular end-diastolic volume; RVEF: right ventricular ejection fraction; RVESV: right ventricular end-systolic volume; RVSV: right ventricular stroke volume i denotes indexation to body surface area calculated according to the Du Bois formula. *denotes p-value <0.05.

**Supplementary Table 4. Multivariable linear regression models showing change in conventional CMR indices per 100g increase in daily meat/fish consumption (confounders + covariates potentially on causal pathway)**

|  | LVEDVi (ml/m^2^) | LVESVi (ml/m^2^) | LVEF (%) | LVSVi (ml/m^2^) | LVMi (g/m^2^) | RVEDVi (ml/m^2^) | RVESVi (ml/m^2^) | RVSVi (ml/m^2^) | RVEF (%) |
| --- | --- | --- | --- | --- | --- | --- | --- | --- | --- |
| Unprocessed red meat | -1.105 | -0.570 | 0.091 | -0.535 | 1.017* | -0.536 | -0.143 | -0.393 | -0.190 |
|  | [-2.284, 0.074] | [-1.306, 0.166] | [-0.460, 0.642] | [-1.285, 0.215] | [0.358, 1.676] | [-2.061, 0.990] | [-1.078, 0.793] | [-1.342, 0.556] | [-0.863, 0.484] |
|  | 0.0663 | 0.1293 | 0.7459 | 0.1619 | 0.0025 | 0.4913 | 0.7651 | 0.4169 | 0.5811 |
| Beef | -1.413 | -0.639 | 0.048 | -0.775 | 1.189* | -1.848 | -1.401 | -0.448 | 0.664 |
|  | [-3.401, 0.575] | [-1.880, 0.603] | [-0.881, 0.976] | [-2.039, 0.489] | [0.078, 2.299] | [-4.392, 0.696] | [-2.960, 0.159] | [-2.030, 1.135] | [-0.459, 1.787] |
|  | 0.1635 | 0.3132 | 0.9201 | 0.2297 | 0.0359 | 0.1544 | 0.0784 | 0.5793 | 0.2463 |
| Lamb | -3.634* | -1.970 | 0.270 | -1.664 | 1.513 | 0.735 | 2.110 | -1.375 | -2.236* |
|  | [-6.981, -0.287] | [-4.060, 0.120] | [-1.294, 1.834] | [-3.792, 0.464] | [-0.357, 3.383] | [-3.681, 5.150] | [-0.597, 4.817] | [-4.123, 1.372] | [-4.186, -0.287] |
|  | 0.0333 | 0.0647 | 0.7347 | 0.1254 | 0.1129 | 0.7443 | 0.1266 | 0.3264 | 0.0245 |
| Pork | -1.060 | -0.651 | 0.207 | -0.408 | 2.621* | -0.051 | 0.589 | -0.640 | -1.081 |
|  | [-4.066, 1.947] | [-2.528, 1.225] | [-1.197, 1.610] | [-2.320, 1.504] | [0.940, 4.301] | [-4.074, 3.972] | [-1.878, 3.056] | [-3.143, 1.863] | [-2.858, 0.696] |
|  | 0.4897 | 0.4963 | 0.7730 | 0.6755 | 0.0022 | 0.9802 | 0.6399 | 0.6163 | 0.2330 |
| Processed meat | -2.079* | -0.822* | -0.094 | -1.257* | 0.051 | -1.961* | -0.473 | -1.487* | -0.500 |
|  | [-3.309, -0.849] | [-1.590, -0.054] | [-0.669, 0.480] | [-2.039, -0.475] | [-0.637, 0.738] | [-3.531, -0.391] | [-1.436, 0.489] | [-2.464, -0.511] | [-1.193, 0.193] |
|  | 9.23$\times$10^-4^ | 0.0359 | 0.7474 | 0.0016 | 0.8849 | 0.0144 | 0.3351 | 0.0028 | 0.1571 |
| Oily fish | 4.340* | 1.828* | 0.102 | 2.512* | 2.317* | 4.062* | 2.168* | 1.894* | -0.402 |
|  | [2.681, 5.999] | [0.792, 2.864] | [-0.673, 0.878] | [1.457, 3.567] | [1.390, 3.244] | [1.930, 6.194] | [0.861, 3.476] | [0.567, 3.220] | [-1.343, 0.539] |
|  | 2.94$\times$10^-7^ | 5.42$\times$10^-4^ | 0.7956 | 3.07$\times$10^-6^ | 9.73$\times$10^-7^ | 1.89$\times$10^-4^ | 0.0012 | 0.0051 | 0.4028 |

**Supplemental Table 4 footnote:** Each cell represents a separate model, adjusted for: age, sex, social deprivation, educational level, smoking, alcohol intake, exercise level, body mass index, hypertension, hypercholesterolaemia, diabetes. First, second, and third row for every CMR measures corresponds to beta coefficient, 95% confidence interval and p-value, respectively. CMR: cardiovascular magnetic resonance; LVEDV: left ventricular end-diastolic volume; LVESV: left ventricular end-systolic volume; LVEF: left ventricular ejection fraction; LVM: left ventricular mass; LVSV: left ventricular stroke volume; RVEDV: right ventricular end-diastolic volume; RVEF: right ventricular ejection fraction; RVESV: right ventricular end-systolic volume; RVSV: right ventricular stroke volume. i denotes indexation to body surface area calculated according to the Du Bois formula. *denotes p-value <0.05

**Supplemental Table 5. Multivariate linear regression models showing change in LV CMR shape radiomics in end-diastole per 100g increase in daily meat/fish consumption**

|  | Unprocessed red meat | Beef | Lamb | Pork | Processed meat | Oily fish |
| --- | --- | --- | --- | --- | --- | --- |
| Volume | 0.0126 | 0.0109 | 0.0033 | 0.0121 | 0.0048 | 0.0347* |
|  | [0.0013, 0.0239] | [-0.0003, 0.0221] | [-0.008, 0.0145] | [0.001, 0.0234] | [-0.0069, 0.0164] | [0.0234, 0.046] |
|  | 0.0285 | 0.0575 | 0.5692 | 0.0335 | 0.4236 | 1.9x10^-9^ |
| Surface Area | 0.024* | 0.0212* | 0.0093 | 0.0206* | 0.014 | 0.0344* |
|  | [0.013, 0.0351] | [0.0102, 0.0322] | [-0.0017, 0.0204] | [0.0096, 0.0316] | [0.0025, 0.0254] | [0.0233, 0.0455] |
|  | 2.09×10^-5^ | 0.0002 | 0.0983 | 0.0002 | 0.0167 | 1.33x10^-9^ |
| Surface Area To Volume Ratio | 0.011 | 0.0096 | 0.0086 | 0.0068 | 0.0107 | -0.025* |
|  | [-0.0018, 0.0238] | [-0.0032, 0.0223] | [-0.0042, 0.0214] | [-0.0059, 0.0196] | [-0.0025, 0.024] | [-0.0379, -0.0122] |
|  | 0.0924 | 0.142 | 0.1855 | 0.2944 | 0.1125 | 0.0001 |
| Sphericity | -0.0447* | -0.0391* | -0.0227 | -0.0352* | -0.0306* | -0.0085 |
|  | [-0.0585, -0.0309] | [-0.0528, -0.0253] | [-0.0364, -0.0089] | [-0.049, -0.0215] | [-0.0449, -0.0164] | [-0.0224, 0.0054] |
|  | 2.21×10^-10^ | 2.53×10^-8^ | 0.0013 | 4.8×10^-7^ | 2.54×10^-5^ | 0.2305 |
| Maximum 3D Diameter | 0.0224* | 0.0211* | 0.0036 | 0.021* | 0.0087 | 0.0205 |
|  | [0.0104, 0.0343] | [0.0092, 0.033] | [-0.0083, 0.0155] | [0.0092, 0.0329] | [-0.0037, 0.021] | [0.0086, 0.0325] |
|  | 0.0002 | 0.0005 | 0.5544 | 0.0005 | 0.1678 | 0.0008 |
| Maximum 2D Diameter (Slice) | 0.0113 | 0.0115 | 0.0045 | 0.0071 | 0.0045 | 0.0151 |
|  | [-0.0019, 0.0245] | [-0.0016, 0.0247] | [-0.0086, 0.0177] | [-0.006, 0.0203] | [-0.0092, 0.0181] | [0.0018, 0.0283] |
|  | 0.0945 | 0.0853 | 0.4985 | 0.2855 | 0.5198 | 0.0259 |
| Maximum 2D Diameter (Column) | 0.0256* | 0.0241* | 0.0077 | 0.0209* | 0.0151 | 0.0289* |
|  | [0.0143, 0.037] | [0.0128, 0.0354] | [-0.0037, 0.019] | [0.0096, 0.0321] | [0.0034, 0.0268] | [0.0175, 0.0403] |
|  | 9.77×10^-6^ | 3.00×10^-5^ | 0.1852 | 0.0003 | 0.0117 | 6.76x10^-7^ |
| Maximum 2D Diameter (Row) | 0.0259* | 0.0237* | 0.0067 | 0.0233* | 0.0124 | 0.0251* |
|  | [0.0141, 0.0377] | [0.012, 0.0355] | [-0.0051, 0.0184] | [0.0116, 0.035] | [0.0002, 0.0246] | [0.0132, 0.0369] |
|  | 1.66×10^-5^ | 7.41×10^-5^ | 0.2667 | 9.87×10^-5^ | 0.0465 | 3.39x10^-5^ |
| Major Axis Length | 0.0279* | 0.0243* | 0.0114 | 0.0231* | 0.0215* | 0.0247* |
|  | [0.0166, 0.0392] | [0.013, 0.0356] | [0.0001, 0.0227] | [0.0118, 0.0343] | [0.0098, 0.0332] | [0.0133, 0.0361] |
|  | 1.39×10^-6^ | 2.37×10^-5^ | 0.048 | 5.77×10^-5^ | 0.0003 | 2.06x10^-5^ |
| Minor Axis Length | 0.0098 | 0.0099 | 0.0021 | 0.0079 | 0.0005 | 0.0285* |
|  | [-0.0026, 0.0221] | [-0.0024, 0.0222] | [-0.0103, 0.0144] | [-0.0044, 0.0202] | [-0.0122, 0.0133] | [0.0161, 0.0409] |
|  | 0.1216 | 0.1149 | 0.7411 | 0.2074 | 0.933 | 6.94x10^-6^ |
| Least Axis Length | 0.0081 | 0.0082 | 0.0028 | 0.0056 | -0.0033 | 0.032* |
|  | [-0.0042, 0.0203] | [-0.004, 0.0204] | [-0.0094, 0.015] | [-0.0065, 0.0178] | [-0.0159, 0.0094] | [0.0197, 0.0443] |
|  | 0.1967 | 0.188 | 0.6539 | 0.3655 | 0.612 | 3.46x10^-7^ |
| Elongation | -0.0186 | -0.0145 | -0.0103 | -0.0154 | -0.0215 | 0.0014 |
|  | [-0.0327, -0.0045] | [-0.0286, -0.0005] | [-0.0244, 0.0038] | [-0.0294, -0.0014] | [-0.036, -0.0069] | [-0.0128, 0.0155] |
|  | 0.0098 | 0.0429 | 0.1518 | 0.0312 | 0.0039 | 0.8509 |
| Flatness | -0.0209 | -0.0167 | -0.01 | -0.0183 | -0.0259* | 0.0036 |
|  | [-0.0351, -0.0068] | [-0.0307, -0.0026] | [-0.0241, 0.0041] | [-0.0323, -0.0043] | [-0.0404, -0.0113] | [-0.0105, 0.0178] |
|  | 0.0036 | 0.0202 | 0.1656 | 0.0105 | 0.0005 | 0.6143 |

**Supplemental Table 5 footnote:** Each cell represents a separate model, adjusted for: age, sex, social deprivation, educational level, smoking, alcohol intake, exercise level. Results are degree of change in radiomics shape feature per 100g increase in daily meat/fish consumption with corresponding 95% confidence intervals and p-values. First, second, and third row for every CMR measures corresponds to beta coefficient, 95% confidence interval and p-value, respectively. Bonferroni adjusted significance threshold p-value =0.0006 (corrected for 78 comparisons).CMR: cardiovascular magnetic resonance; LV: left ventricle.

**Supplemental Table 6. Multivariate linear regression models showing change in LV CMR shape radiomics in end-systole per 100g increase in daily meat/fish consumption.**

|  | Unprocessed red meat | Beef | Lamb | Pork | Processed meat | Oily fish |
| --- | --- | --- | --- | --- | --- | --- |
| Volume | 0.0076 | 0.0082 | -0.0008 | 0.0074 | 0.0011 | 0.0263* |
|  | [-0.0041, 0.0194] | [-0.0035, 0.0199] | [-0.0126, 0.0109] | [-0.0043, 0.0191] | [-0.011, 0.0132] | [0.0145, 0.0381] |
|  | 0.2015 | 0.1704 | 0.8879 | 0.2131 | 0.8611 | 1.2x10^-5^ |
| Surface Area | 0.0176* | 0.0177* | 0.005 | 0.0135* | 0.008 | 0.0267* |
|  | [0.0063, 0.029] | [0.0064, 0.029] | [-0.0063, 0.0163] | [0.0023, 0.0248] | [-0.0037, 0.0197] | [0.0153, 0.0381] |
|  | 0.0023 | 0.0021 | 0.3824 | 0.0182 | 0.1813 | 4.16x10^-6^ |
| Surface Area To Volume Ratio | 0.0124 | 0.0095 | 0.0116 | 0.0076 | 0.0078 | -0.015* |
|  | [-0.0003, 0.0252] | [-0.0032, 0.0222] | [-0.0011, 0.0244] | [-0.0051, 0.0203] | [-0.0054, 0.021] | [-0.0278, -0.0022] |
|  | 0.0562 | 0.144 | 0.0736 | 0.2405 | 0.2457 | 0.0218 |
| Sphericity | -0.0438* | -0.0405* | -0.0245* | -0.0292* | -0.0235* | -0.0097 |
|  | [-0.0574, -0.0301] | [-0.0542, -0.0269] | [-0.0382, -0.0108] | [-0.0428, -0.0156] | [-0.0376, -0.0094] | [-0.0235, 0.004] |
|  | 3.69×10^-10^ | 5.67×10^-9^ | 0.0004 | 2.59×10^-5^ | 0.0011 | 0.1654 |
| Maximum 3D Diameter | 0.0182* | 0.0182* | 0.0087 | 0.011 | 0.0048 | 0.0149* |
|  | [0.0062, 0.0302] | [0.0062, 0.0301] | [-0.0033, 0.0206] | [-0.0009, 0.023] | [-0.0076, 0.0172] | [0.0029, 0.027] |
|  | 0.003 | 0.0029 | 0.156 | 0.0697 | 0.4477 | 0.0153 |
| Maximum 2D Diameter (Slice) | 0.0141* | 0.0162* | 0.0058 | 0.0057 | 0.0039 | 0.0109 |
|  | [0.0014, 0.0268] | [0.0035, 0.0289] | [-0.0069, 0.0185] | [-0.0069, 0.0184] | [-0.0092, 0.0171] | [-0.0019, 0.0237] |
|  | 0.0301 | 0.0123 | 0.3676 | 0.3755 | 0.5598 | 0.0945 |
| Maximum 2D Diameter (Column) | 0.0173* | 0.0161* | 0.0076 | 0.0127* | 0.0066 | 0.0186* |
|  | [0.0055, 0.0291] | [0.0043, 0.0278] | [-0.0041, 0.0194] | [0.001, 0.0244] | [-0.0056, 0.0187] | [0.0067, 0.0304] |
|  | 0.0039 | 0.0072 | 0.2024 | 0.0335 | 0.2886 | 0.0021 |
| Maximum 2D Diameter (Row) | 0.0169* | 0.0163* | 0.0085 | 0.011 | 0.0086 | 0.0186* |
|  | [0.005, 0.0287] | [0.0045, 0.0281] | [-0.0034, 0.0203] | [-0.0008, 0.0228] | [-0.0037, 0.0208] | [0.0067, 0.0305] |
|  | 0.0052 | 0.0068 | 0.1608 | 0.067 | 0.1693 | 0.0022 |
| Major Axis Length | 0.0214* | 0.02* | 0.0107 | 0.0141* | 0.0131* | 0.0188* |
|  | [0.0099, 0.0329] | [0.0085, 0.0314] | [-0.0007, 0.0222] | [0.0027, 0.0255] | [0.0012, 0.025] | [0.0073, 0.0304] |
|  | 0.0003 | 0.0006 | 0.0665 | 0.0153 | 0.0307 | 0.0014 |
| Minor Axis Length | 0.0135* | 0.0161* | 0.0028 | 0.0074 | 0.0038 | 0.0197* |
|  | [0.0013, 0.0257] | [0.004, 0.0283] | [-0.0094, 0.0149] | [-0.0047, 0.0195] | [-0.0087, 0.0164] | [0.0075, 0.0319] |
|  | 0.0299 | 0.009 | 0.654 | 0.2298 | 0.5486 | 0.0016 |
| Least Axis Length | 0.0091 | 0.0117 | -0.0009 | 0.006 | -0.0026 | 0.0228* |
|  | [-0.003, 0.0212] | [-0.0004, 0.0238] | [-0.013, 0.0112] | [-0.0061, 0.018] | [-0.0152, 0.0099] | [0.0106, 0.035] |
|  | 0.1418 | 0.0579 | 0.8867 | 0.3332 | 0.6825 | 0.0003 |
| Elongation | -0.0086 | -0.0037 | -0.0089 | -0.0079 | -0.0113 | 0.0009 |
|  | [-0.0229, 0.0057] | [-0.0179, 0.0105] | [-0.0231, 0.0054] | [-0.022, 0.0063] | [-0.026, 0.0035] | [-0.0134, 0.0153] |
|  | 0.2373 | 0.6126 | 0.2218 | 0.2769 | 0.1338 | 0.8975 |
| Flatness | -0.0138 | -0.0087 | -0.0126 | -0.0105 | -0.0181* | 0.0029 |
|  | [-0.028, 0.0004] | [-0.0229, 0.0054] | [-0.0268, 0.0016] | [-0.0246, 0.0037] | [-0.0328, -0.0034] | [-0.0114, 0.0172] |
|  | 0.0569 | 0.2266 | 0.0823 | 0.1471 | 0.0157 | 0.6946 |

**Supplemental Table 6 footnote:** Each cell represents a separate model, adjusted for: age, sex, social deprivation, educational level, smoking, alcohol intake, exercise level. Results are degree of change in radiomics shape feature per 100g increase in daily meat/fish consumption with corresponding 95% confidence intervals and p-values. First, second, and third row for every CMR measures corresponds to beta coefficient, 95% confidence interval and p-value, respectively. Bonferroni adjusted significance threshold p-value =0.0006 (corrected for 78 comparisons).CMR: cardiovascular magnetic resonance; LV: left ventricle.

**Supplemental Table 7. Multivariate linear regression models showing change in RV CMR shape radiomics in end-diastole per 100g increase in daily meat/fish consumption**

|  | Unprocessed red meat | Beef | Lamb | Pork | Processed meat | Oily fish |
| --- | --- | --- | --- | --- | --- | --- |
| Volume | 0.0079 | 0.0054 | 0.0044 | 0.0069 | 0.008 | 0.0281* |
|  | [-0.0028, 0.0185] | [-0.0052, 0.016] | [-0.0062, 0.015] | [-0.0037, 0.0175] | [-0.003, 0.019] | [0.0174, 0.0388] |
|  | 0.1479 | 0.3177 | 0.4173 | 0.2006 | 0.152 | 2.61x10^-7^ |
| Surface Area | 0.0009 | -0.0017 | -0.0004 | 0.0043 | 0.0006 | 0.0336* |
|  | [-0.0099, 0.0117] | [-0.0125, 0.009] | [-0.0111, 0.0104] | [-0.0064, 0.015] | [-0.0105, 0.0118] | [0.0228, 0.0444] |
|  | 0.8687 | 0.7495 | 0.9424 | 0.4286 | 0.912 | 1.13x10^-9^ |
| Surface Area To Volume Ratio | -0.022* | -0.0214* | -0.0127 | -0.0115 | -0.0197 | -0.013 |
|  | [-0.0338, -0.0102] | [-0.0332, -0.0096] | [-0.0245, -0.0009] | [-0.0233, 0.0003] | [-0.0319, -0.0075] | [-0.0249, -0.0012] |
|  | 0.0003 | 0.0004 | 0.0344 | 0.0552 | 0.0016 | 0.0316 |
| Sphericity | 0.0275* | 0.0289* | 0.0181 | 0.0104 | 0.0271* | -0.0215 |
|  | [0.0132, 0.0418] | [0.0147, 0.0431] | [0.0038, 0.0323] | [-0.0038, 0.0246] | [0.0124, 0.0419] | [-0.0359, -0.0072] |
|  | 0.0002 | 6.79×10^-5^ | 0.0129 | 0.1519 | 0.0003 | 0.0033 |
| Maximum 3D Diameter | 0.0015 | 0.0018 | -0.0055 | 0.0044 | -0.004 | 0.0279* |
|  | [-0.0101, 0.0131] | [-0.0098, 0.0133] | [-0.017, 0.0061] | [-0.0071, 0.0159] | [-0.016, 0.008] | [0.0163, 0.0395] |
|  | 0.7979 | 0.7626 | 0.3541 | 0.4512 | 0.5112 | 2.59x10^-6^ |
| Maximum 2D Diameter (Slice) | -0.0246* | -0.0229* | -0.0163 | -0.0145 | -0.0202 | 0.0176 |
|  | [-0.0375, -0.0117] | [-0.0358, -0.01] | [-0.0292, -0.0034] | [-0.0273, -0.0017] | [-0.0336, -0.0069] | [0.0046, 0.0305] |
|  | 0.0002 | 0.0005 | 0.013 | 0.0269 | 0.003 | 0.008 |
| Maximum 2D Diameter (Column) | 0.0139 | 0.0125 | 0.0034 | 0.0125 | 0.0143 | 0.0379* |
|  | [0.0019, 0.026] | [0.0005, 0.0246] | [-0.0087, 0.0154] | [0.0005, 0.0245] | [0.0018, 0.0268] | [0.0258, 0.05] |
|  | 0.0238 | 0.0409 | 0.582 | 0.041 | 0.0248 | 9.36x10^-10^ |
| Maximum 2D Diameter (Row) | 0.0041 | -0.003 | 0.0093 | 0.0062 | 0.0029 | 0.0239* |
|  | [-0.0081, 0.0164] | [-0.0152, 0.0092] | [-0.0029, 0.0215] | [-0.006, 0.0183] | [-0.0097, 0.0155] | [0.0116, 0.0362] |
|  | 0.5086 | 0.6321 | 0.1355 | 0.3205 | 0.6543 | 0.0001 |
| Major Axis Length | -0.0025 | -0.0039 | -0.0075 | 0.0044 | -0.0131 | 0.0255* |
|  | [-0.0142, 0.0093] | [-0.0156, 0.0078] | [-0.0192, 0.0043] | [-0.0073, 0.0161] | [-0.0252, -0.001] | [0.0137, 0.0373] |
|  | 0.6785 | 0.5165 | 0.2117 | 0.4596 | 0.0341 | 2.29x10^-5^ |
| Minor Axis Length | -0.0085 | -0.0098 | -0.0035 | -0.0044 | -0.0066 | 0.0266* |
|  | [-0.0208, 0.0038] | [-0.0221, 0.0024] | [-0.0158, 0.0088] | [-0.0166, 0.0079] | [-0.0193, 0.0061] | [0.0143, 0.039] |
|  | 0.1741 | 0.1158 | 0.5794 | 0.484 | 0.3095 | 2.41x10^-5^ |
| Least Axis Length | 0.0175 | 0.0169 | 0.0077 | 0.0117 | 0.0232* | 0.0277* |
|  | [0.0056, 0.0295] | [0.005, 0.0288] | [-0.0043, 0.0196] | [-0.0001, 0.0236] | [0.0109, 0.0355] | [0.0157, 0.0397] |
|  | 0.004 | 0.0053 | 0.2074 | 0.0529 | 0.0002 | 6.02x10^-6^ |
| Elongation | -0.0056 | -0.0061 | 0.0038 | -0.0077 | 0.0048 | 0.0068 |
|  | [-0.0199, 0.0087] | [-0.0203, 0.0081] | [-0.0105, 0.018] | [-0.0219, 0.0065] | [-0.0099, 0.0196] | [-0.0075, 0.0212] |
|  | 0.4403 | 0.4018 | 0.6042 | 0.2861 | 0.5206 | 0.3511 |
| Flatness | 0.019 | 0.0193 | 0.0143 | 0.0076 | 0.0336* | 0.0048 |
|  | [0.0049, 0.033] | [0.0053, 0.0333] | [0.0003, 0.0283] | [-0.0064, 0.0216] | [0.0191, 0.0482] | [-0.0093, 0.0189] |
|  | 0.0082 | 0.007 | 0.046 | 0.2872 | 5.64×10^-6^ | 0.5056 |

**Supplemental Table 7 footnote:** Each cell represents a separate model, adjusted for: age, sex, social deprivation, educational level, smoking, alcohol intake, exercise level. Results are degree of change in radiomics shape feature per 100g increase in daily meat/fish consumption with corresponding 95% confidence intervals and p-values. First, second, and third row for every CMR measures corresponds to beta coefficient, 95% confidence interval and p-value, respectively. Bonferroni adjusted significance threshold p-value =0.0006 (corrected for 78 comparisons).CMR: cardiovascular magnetic resonance; RV: right ventricle.

**Supplemental Table 8. Multivariate linear regression models showing change in RV CMR shape radiomics in end-systole per 100g increase in daily meat/fish consumption**

|  | Unprocessed red meat | Beef | Lamb | Pork | Processed meat | Oily fish |
| --- | --- | --- | --- | --- | --- | --- |
| Volume | 0.0105 | 0.0057 | 0.0106 | 0.0078 | 0.0114 | 0.0255* |
|  | [-0.0003, 0.0212] | [-0.0051, 0.0164] | [-0.0001, 0.0214] | [-0.0029, 0.0185] | [0.0003, 0.0226] | [0.0147, 0.0363] |
|  | 0.0576 | 0.2989 | 0.0527 | 0.1529 | 0.0443 | 4.03x10^-6^ |
| Surface Area | 0.0071 | 0.0035 | 0.0069 | 0.0059 | 0.0063 | 0.0283* |
|  | [-0.0036, 0.0177] | [-0.0071, 0.0142] | [-0.0037, 0.0176] | [-0.0047, 0.0165] | [-0.0047, 0.0173] | [0.0176, 0.039] |
|  | 0.1938 | 0.5145 | 0.2014 | 0.2742 | 0.2597 | 2.29x10^-7^ |
| Surface Area To Volume Ratio | -0.0176 | -0.0134 | -0.015 | -0.0106 | -0.0181 | -0.0176* |
|  | [-0.0293, -0.0059] | [-0.0251, -0.0018] | [-0.0267, -0.0033] | [-0.0222, 0.001] | [-0.0302, -0.006] | [-0.0294, -0.0059] |
|  | 0.0031 | 0.024 | 0.0118 | 0.0736 | 0.0033 | 0.0033 |
| Sphericity | 0.0196 | 0.0153 | 0.0177 | 0.0108 | 0.0236 | -0.0092 |
|  | [0.0053, 0.0339] | [0.001, 0.0295] | [0.0034, 0.032] | [-0.0034, 0.025] | [0.0088, 0.0383] | [-0.0236, 0.0052] |
|  | 0.0071 | 0.0359 | 0.0151 | 0.1361 | 0.0018 | 0.2096 |
| Maximum 3D Diameter | 0.0109 | 0.0111 | 0.006 | 0.0044 | 0.0094 | 0.0294* |
|  | [-0.0009, 0.0226] | [-0.0005, 0.0228] | [-0.0057, 0.0177] | [-0.0072, 0.0161] | [-0.0027, 0.0215] | [0.0176, 0.0412] |
|  | 0.0694 | 0.0617 | 0.3159 | 0.4572 | 0.129 | 9.88x10^-7^ |
| Maximum 2D Diameter (Slice) | 0.001 | -0.0024 | 0.0041 | 0.0019 | 0.0023 | 0.0162 |
|  | [-0.0119, 0.014] | [-0.0153, 0.0105] | [-0.0089, 0.017] | [-0.0109, 0.0148] | [-0.0111, 0.0156] | [0.0032, 0.0293] |
|  | 0.8794 | 0.7178 | 0.5383 | 0.7666 | 0.7394 | 0.0144 |
| Maximum 2D Diameter (Column) | 0.0177 | 0.0158 | 0.0069 | 0.0145 | 0.0216 | 0.0285* |
|  | [0.0054, 0.0301] | [0.0036, 0.0281] | [-0.0054, 0.0192] | [0.0023, 0.0268] | [0.0089, 0.0343] | [0.0161, 0.0409] |
|  | 0.0048 | 0.0114 | 0.2696 | 0.02 | 0.0009 | 6.38x10^-6^ |
| Maximum 2D Diameter (Row) | 0.0049 | 0.0001 | 0.0097 | 0.0035 | 0.0046 | 0.016 |
|  | [-0.0079, 0.0177] | [-0.0127, 0.0129] | [-0.0031, 0.0225] | [-0.0093, 0.0162] | [-0.0086, 0.0178] | [0.0031, 0.0289] |
|  | 0.452 | 0.9873 | 0.1384 | 0.5928 | 0.4951 | 0.015 |
| Major Axis Length | 0.0061 | 0.0055 | -0.0004 | 0.0065 | 0.0001 | 0.0149 |
|  | [-0.0061, 0.0183] | [-0.0067, 0.0176] | [-0.0126, 0.0117] | [-0.0056, 0.0186] | [-0.0125, 0.0126] | [0.0026, 0.0271] |
|  | 0.3259 | 0.377 | 0.9472 | 0.2958 | 0.9926 | 0.0172 |
| Minor Axis Length | 0.0069 | 0.002 | 0.0119 | 0.0038 | 0.0028 | 0.0238* |
|  | [-0.0053, 0.019] | [-0.0101, 0.0141] | [-0.0002, 0.024] | [-0.0083, 0.0159] | [-0.0098, 0.0154] | [0.0116, 0.0361] |
|  | 0.2673 | 0.7484 | 0.0542 | 0.5358 | 0.6623 | 0.0001 |
| Least Axis Length | 0.0119 | 0.0101 | 0.0058 | 0.0092 | 0.015 | 0.0276* |
|  | [0.0005, 0.0232] | [-0.0013, 0.0214] | [-0.0055, 0.0172] | [-0.0021, 0.0205] | [0.0033, 0.0268] | [0.0161, 0.039] |
|  | 0.0411 | 0.0818 | 0.3151 | 0.1123 | 0.0123 | 2.27x10^-6^ |
| Elongation | 0.0055 | 0.0013 | 0.0145 | -0.0003 | 0.0043 | 0.0141 |
|  | [-0.0085, 0.0195] | [-0.0126, 0.0153] | [0.0005, 0.0284] | [-0.0142, 0.0136] | [-0.0102, 0.0187] | [0, 0.0282] |
|  | 0.4419 | 0.8503 | 0.0424 | 0.9643 | 0.5615 | 0.0496 |
| Flatness | 0.0086 | 0.0081 | 0.0073 | 0.0035 | 0.0163 | 0.0162 |
|  | [-0.0055, 0.0227] | [-0.006, 0.0222] | [-0.0068, 0.0214] | [-0.0105, 0.0176] | [0.0018, 0.0309] | [0.002, 0.0304] |
|  | 0.2336 | 0.2595 | 0.312 | 0.6207 | 0.0281 | 0.0252 |

**Supplemental Table 8 footnote:** Each cell represents a separate model, adjusted for: age, sex, social deprivation, educational level, smoking, alcohol intake, exercise level. Results are degree of change in radiomics shape feature per 100g increase in daily meat/fish consumption with corresponding 95% confidence intervals and p-values. First, second, and third row for every CMR measures corresponds to beta coefficient, 95% confidence interval and p-value, respectively. Bonferroni adjusted significance threshold p-value =0.0006 (corrected for 78 comparisons).CMR: cardiovascular magnetic resonance; RV: right ventricle.

**Supplemental Table 9. Multivariate linear regression models showing change in LV myocardium first-order radiomics in end-diastole per 100g increase in daily meat/fish consumption.**

|  | Unprocessed red meat | Beef | Lamb | Pork | Processed meat | Oily fish |
| --- | --- | --- | --- | --- | --- | --- |
| Energy | -0.0528* | -0.049* | -0.0354* | -0.0294* | -0.049* | 0.0307* |
|  | [-0.0656, -0.04] | [-0.0618, -0.0363] | [-0.0482, -0.0226] | [-0.0421, -0.0167] | [-0.0622, -0.0358] | [0.0178, 0.0436] |
|  | 6.86×10^-16^ | 5.03×10^-14^ | 5.86×10^-8^ | 6.17×10^-6^ | 4.02×10^-13^ | 2.95x10×10^-6^ |
| Total Energy | -0.0459* | -0.0427* | -0.0327* | -0.0238* | -0.0427* | 0.0322* |
|  | [-0.0586, -0.0333] | [-0.0553, -0.0301] | [-0.0453, -0.0201] | [-0.0364, -0.0113] | [-0.0558, -0.0296] | [0.0195, 0.045] |
|  | 1.16×10^-12^ | 3.19×10^-11^ | 3.82×10^-7^ | 0.0002 | 1.51×10^-10^ | 6.81x10×10^-7^ |
| Entropy | -0.0413* | -0.0373* | -0.024* | -0.0263* | -0.0361* | -0.0082 |
|  | [-0.0544, -0.0281] | [-0.0504, -0.0243] | [-0.0371, -0.0109] | [-0.0394, -0.0133] | [-0.0496, -0.0225] | [-0.0214, 0.005] |
|  | 7.33×10^-10^ | 2.22×10^-8^ | 0.0003 | 7.67×10^-5^ | 1.87×10^-7^ | 0.2228 |
| Minimum | -0.0483* | -0.0401* | -0.0355* | -0.0322* | -0.041* | 0.006 |
|  | [-0.0625, -0.0341] | [-0.0542, -0.0259] | [-0.0497, -0.0213] | [-0.0464, -0.0181] | [-0.0557, -0.0263] | [-0.0083, 0.0203] |
|  | 2.83×10^-11^ | 3.05×10^-8^ | 9.64×10^-7^ | 7.9×10^-6^ | 4.6×10^-8^ | 0.4109 |
| 10th Percentile | -0.0679* | -0.0615* | -0.0462* | -0.0395* | -0.062* | 0.0184 |
|  | [-0.0817, -0.0541] | [-0.0752, -0.0477] | [-0.06, -0.0324] | [-0.0533, -0.0258] | [-0.0762, -0.0477] | [0.0045, 0.0323] |
|  | 5.32×10^-22^ | 1.93×10^-18^ | 5.12×10^-11^ | 1.68×10^-8^ | 1.69×10^-17^ | 0.0096 |
| 90th Percentile | -0.0775* | -0.0705* | -0.0506* | -0.0459* | -0.0678* | 0.0099 |
|  | [-0.0916, -0.0635] | [-0.0845, -0.0565] | [-0.0646, -0.0365] | [-0.0599, -0.0319] | [-0.0823, -0.0532] | [-0.0043, 0.0241] |
|  | 4.04×10^-27^ | 7.42×10^-23^ | 1.81×10^-12^ | 1.39×10^-10^ | 7.16×10^-20^ | 0.1721 |
| Maximum | -0.0554* | -0.0549* | -0.0275* | -0.0344* | -0.0502* | 0.0156 |
|  | [-0.0696, -0.0413] | [-0.069, -0.0408] | [-0.0416, -0.0134] | [-0.0484, -0.0204] | [-0.0648, -0.0356] | [0.0014, 0.0298] |
|  | 1.46×10^-14^ | 2.07×10^-14^ | 0.0001 | 1.57×10^-6^ | 1.63×10^-11^ | 0.0317 |
| Mean | -0.0768* | -0.069* | -0.0514* | -0.0456* | -0.0676* | 0.0151 |
|  | [-0.0908, -0.0628] | [-0.0829, -0.055] | [-0.0655, -0.0374] | [-0.0596, -0.0317] | [-0.082, -0.0531] | [0.001, 0.0292] |
|  | 7.91×10^-27^ | 4.04×10^-22^ | 6.15×10^-13^ | 1.48×10^-10^ | 6.59×10^-20^ | 0.036 |
| Median | -0.0749* | -0.0667* | -0.0506* | -0.0447* | -0.0646* | 0.0149 |
|  | [-0.0889, -0.0609] | [-0.0806, -0.0528] | [-0.0646, -0.0367] | [-0.0586, -0.0309] | [-0.0791, -0.0502] | [0.0009, 0.029] |
|  | 8.6×10^-26^ | 5.83×10^-21^ | 1.14×10^-12^ | 2.82×10^-10^ | 1.8×10^-18^ | 0.0373 |
| Interquartile Range | -0.0119 | -0.0096 | -0.0079 | -0.0079 | -0.0126 | -0.0108 |
|  | [-0.0253, 0.0015] | [-0.023, 0.0037] | [-0.0213, 0.0054] | [-0.0212, 0.0054] | [-0.0264, 0.0013] | [-0.0243, 0.0026] |
|  | 0.082 | 0.156 | 0.2452 | 0.2457 | 0.0746 | 0.1147 |
| Range | -0.0518* | -0.0525* | -0.0239 | -0.0318* | -0.0473* | 0.0157 |
|  | [-0.0659, -0.0377] | [-0.0665, -0.0384] | [-0.038, -0.0098] | [-0.0458, -0.0178] | [-0.0619, -0.0327] | [0.0015, 0.0299] |
|  | 6.73×10^-13^ | 2.72×10^-13^ | 0.0009 | 9.05×10^-6^ | 2.18×10^-10^ | 0.0304 |
| Mean Absolute Deviation | -0.0349* | -0.0325* | -0.0194 | -0.0217 | -0.0277* | -0.0067 |
|  | [-0.048, -0.0219] | [-0.0455, -0.0195] | [-0.0324, -0.0064] | [-0.0347, -0.0088] | [-0.0412, -0.0142] | [-0.0198, 0.0064] |
|  | 1.52×10^-7^ | 9.37×10^-7^ | 0.0035 | 0.001 | 5.69×10^-5^ | 0.318 |
| Robust Mean Absolute Deviation | -0.0148* | -0.0129 | -0.0092 | -0.0093 | -0.0139 | -0.0115 |
|  | [-0.0281, -0.0016] | [-0.0261, 0.0003] | [-0.0224, 0.004] | [-0.0225, 0.0038] | [-0.0275, -0.0002] | [-0.0248, 0.0018] |
|  | 0.0285 | 0.0559 | 0.1731 | 0.165 | 0.0472 | 0.0913 |
| Root Mean Squared | -0.0779* | -0.0702* | -0.0517* | -0.0463* | -0.0679* | 0.0147 |
|  | [-0.0919, -0.0638] | [-0.0842, -0.0562] | [-0.0657, -0.0377] | [-0.0603, -0.0324] | [-0.0824, -0.0534] | [0.0005, 0.0288] |
|  | 2.02×10^-27^ | 9.86×10^-23^ | 5.53×10^-13^ | 8.49×10^-11^ | 5.35×10^-20^ | 0.0425 |
| Skewness | -0.0021 | -0.0097 | 0.0124 | -0.0026 | -0.0023 | 0.0078 |
|  | [-0.0162, 0.012] | [-0.0238, 0.0043] | [-0.0017, 0.0265] | [-0.0166, 0.0114] | [-0.0169, 0.0123] | [-0.0063, 0.022] |
|  | 0.7751 | 0.1746 | 0.0835 | 0.7131 | 0.7553 | 0.2789 |
| Kurtosis | -0.0183 | -0.0238 | 0.0013 | -0.0119 | -0.0195* | 0.0134 |
|  | [-0.0325, -0.0041] | [-0.038, -0.0097] | [-0.0128, 0.0155] | [-0.026, 0.0022] | [-0.0341, -0.0048] | [-0.0008, 0.0277] |
|  | 0.0115 | 0.0009 | 0.8542 | 0.0971 | 0.0092 | 0.065 |
| Variance | -0.0427* | -0.0412* | -0.0216 | -0.0268* | -0.0279* | -0.0013 |
|  | [-0.0565, -0.029] | [-0.0549, -0.0275] | [-0.0353, -0.0079] | [-0.0405, -0.0131] | [-0.0421, -0.0137] | [-0.0152, 0.0125] |
|  | 1.18×10^-9^ | 3.85×10^-9^ | 0.0021 | 0.0001 | 0.0001 | 0.8523 |
| Uniformity | 0.0204 | 0.0157 | 0.0143 | 0.0147 | 0.0226 | 0.0139 |
|  | [0.007, 0.0339] | [0.0023, 0.0291] | [0.0009, 0.0278] | [0.0014, 0.0281] | [0.0087, 0.0365] | [0.0004, 0.0274] |
|  | 0.0029 | 0.0217 | 0.0363 | 0.0308 | 0.0014 | 0.0442 |

**Supplemental Table 9 footnote:** Each cell represents a separate model, adjusted for: age, sex, social deprivation, educational level, smoking, alcohol intake, exercise level. Results are degree of change in radiomics first-order feature per 100g increase in daily meat/fish consumption with corresponding 95% confidence intervals and p-values. First, second, and third row for every CMR measures corresponds to beta coefficient, 95% confidence interval and p-value, respectively. Bonferroni adjusted significance threshold p-value =0.0004 (corrected for 108 comparisons). CMR: cardiovascular magnetic resonance; LV: left ventricle.

**Supplemental Table 10. Multivariate linear regression models showing change in LV myocardium first-order radiomics in end-systole per 100g increase in daily meat/fish consumption**

|  | Unprocessed red meat | Beef | Lamb | Pork | Processed meat | Oily fish |
| --- | --- | --- | --- | --- | --- | --- |
| Energy | -0.0577* | -0.0542* | -0.0365* | -0.0333* | -0.0567* | 0.0292* |
|  | [-0.0703, -0.0451] | [-0.0668, -0.0416] | [-0.0492, -0.0239] | [-0.0458, -0.0207] | [-0.0697, -0.0436] | [0.0164, 0.0419] |
|  | 3.9×10^-19^ | 3.27×10^-17^ | 1.41×10^-8^ | 2.19×10^-7^ | 1.9×10^-17^ | 7.03x10^-6^ |
| Total Energy | -0.0526* | -0.0496* | -0.0346* | -0.029* | -0.0521* | 0.0302* |
|  | [-0.0651, -0.0401] | [-0.062, -0.0371] | [-0.0471, -0.0221] | [-0.0415, -0.0166] | [-0.065, -0.0392] | [0.0177, 0.0428] |
|  | 1.69×10^-16^ | 6.21×10^-15^ | 5.54×10^-8^ | 4.75×10^-6^ | 2.8×10^-15^ | 2.45x10^-6^ |
| Entropy | -0.034* | -0.0296* | -0.0182* | -0.0249* | -0.0305* | 0.0063 |
|  | [-0.048, -0.0199] | [-0.0436, -0.0157] | [-0.0322, -0.0041] | [-0.0388, -0.0109] | [-0.045, -0.016] | [-0.0078, 0.0204] |
|  | 2.15×10^-6^ | 3.3×10^-5^ | 0.0111 | 0.0005 | 3.88×10^-5^ | 0.3831 |
| Minimum | -0.0433* | -0.038* | -0.0338* | -0.0241* | -0.039* | 0.007 |
|  | [-0.0575, -0.0291] | [-0.0522, -0.0239] | [-0.048, -0.0196] | [-0.0382, -0.01] | [-0.0537, -0.0243] | [-0.0073, 0.0214] |
|  | 2.44×10^-9^ | 1.46×10^-7^ | 3.14×10^-6^ | 0.0008 | 1.96×10^-7^ | 0.3344 |
| 10th Percentile | -0.0796* | -0.0725* | -0.0532* | -0.0465* | -0.0743* | 0.0155* |
|  | [-0.0934, -0.0657] | [-0.0863, -0.0587] | [-0.0671, -0.0394] | [-0.0603, -0.0327] | [-0.0887, -0.06] | [0.0016, 0.0295] |
|  | 2.82×10^-29^ | 9.37×10^-25^ | 5.4×10^-14^ | 4.12×10^-11^ | 3.05×10^-24^ | 0.0292 |
| 90th Percentile | -0.0811* | -0.0744* | -0.0518* | -0.0484* | -0.0745* | 0.0182* |
|  | [-0.0949, -0.0673] | [-0.0881, -0.0606] | [-0.0656, -0.038] | [-0.0622, -0.0347] | [-0.0887, -0.0602] | [0.0043, 0.0321] |
|  | 1.31×10^-30^ | 3.24×10^-26^ | 1.85×10^-13^ | 4.8×10^-12^ | 1.53×10^-24^ | 0.0103 |
| Maximum | -0.0701* | -0.0638* | -0.0427* | -0.0444* | -0.0619* | 0.019* |
|  | [-0.0842, -0.056] | [-0.0778, -0.0497] | [-0.0568, -0.0287] | [-0.0584, -0.0304] | [-0.0765, -0.0474] | [0.0049, 0.0332] |
|  | 1.7×10^-22^ | 5.16×10^-19^ | 2.62×10^-9^ | 5.07×10^-10^ | 7.24×10^-17^ | 0.0084 |
| Mean | -0.0834* | -0.0759* | -0.0541* | -0.05* | -0.0771* | 0.0163* |
|  | [-0.0971, -0.0696] | [-0.0896, -0.0621] | [-0.0679, -0.0403] | [-0.0638, -0.0363] | [-0.0913, -0.0629] | [0.0024, 0.0302] |
|  | 2.47×10^-32^ | 2.88×10^-27^ | 1.44×10^-14^ | 9.12×10^-13^ | 3.1×10^-26^ | 0.0213 |
| Median | -0.0824* | -0.0744* | -0.0534* | -0.0504* | -0.0763* | 0.0142* |
|  | [-0.0962, -0.0687] | [-0.0882, -0.0607] | [-0.0671, -0.0396] | [-0.0641, -0.0367] | [-0.0905, -0.0621] | [0.0003, 0.028] |
|  | 9.84×10^-32^ | 2.23×10^-26^ | 3.02×10^-14^ | 6.08×10^-13^ | 8.34×10^-26^ | 0.0455 |
| Interquartile Range | -0.0236* | -0.0201* | -0.0159* | -0.0152* | -0.0199* | 0.003 |
|  | [-0.0376, -0.0096] | [-0.0341, -0.0061] | [-0.0299, -0.0019] | [-0.0291, -0.0012] | [-0.0344, -0.0055] | [-0.0111, 0.0172] |
|  | 0.001 | 0.0049 | 0.026 | 0.033 | 0.007 | 0.6725 |
| Range | -0.0664* | -0.0607* | -0.0385* | -0.0429* | -0.0584* | 0.0193* |
|  | [-0.0804, -0.0523] | [-0.0747, -0.0467] | [-0.0525, -0.0244] | [-0.0569, -0.0289] | [-0.073, -0.0439] | [0.0051, 0.0335] |
|  | 2.63×10^-20^ | 2.26×10^-17^ | 8.29×10^-8^ | 1.9×10^-9^ | 3.62×10^-15^ | 0.0076 |
| Mean Absolute Deviation | -0.0354* | -0.0326* | -0.0204* | -0.0224* | -0.0283* | 0.0087 |
|  | [-0.0495, -0.0214] | [-0.0465, -0.0186] | [-0.0344, -0.0064] | [-0.0364, -0.0085] | [-0.0428, -0.0138] | [-0.0054, 0.0228] |
|  | 7.38×10^-7^ | 4.99×10^-6^ | 0.0043 | 0.0016 | 0.0001 | 0.2251 |
| Robust Mean Absolute Deviation | -0.0248* | -0.0213* | -0.0161* | -0.0163* | -0.0201* | 0.0063 |
|  | [-0.0388, -0.0108] | [-0.0353, -0.0073] | [-0.0301, -0.0021] | [-0.0302, -0.0023] | [-0.0346, -0.0056] | [-0.0078, 0.0205] |
|  | 0.0005 | 0.0028 | 0.0242 | 0.0221 | 0.0065 | 0.3784 |
| Root Mean Squared | -0.0838* | -0.0763* | -0.0541* | -0.0503* | -0.077* | 0.0165* |
|  | [-0.0975, -0.07] | [-0.09, -0.0626] | [-0.0679, -0.0404] | [-0.0641, -0.0366] | [-0.0912, -0.0628] | [0.0026, 0.0304] |
|  | 1.22×10^-32^ | 1.42×10^-27^ | 1.38×10^-14^ | 6.62×10^-13^ | 3.51×10^-26^ | 0.0198 |
| Skewness | 0.0193* | 0.0159* | 0.0111 | 0.0147* | 0.0245* | 0.0043 |
|  | [0.0052, 0.0334] | [0.0019, 0.03] | [-0.003, 0.0252] | [0.0007, 0.0287] | [0.01, 0.0391] | [-0.0099, 0.0185] |
|  | 0.0075 | 0.0263 | 0.1225 | 0.0402 | 0.001 | 0.5503 |
| Kurtosis | -0.0296* | -0.0275* | -0.0154* | -0.0201* | -0.0242* | 0.0063 |
|  | [-0.0439, -0.0153] | [-0.0417, -0.0133] | [-0.0296, -0.0011] | [-0.0343, -0.0059] | [-0.0389, -0.0094] | [-0.0081, 0.0206] |
|  | 4.85×10^-5^ | 0.0002 | 0.0344 | 0.0054 | 0.0013 | 0.3911 |
| Variance | -0.0406* | -0.0382* | -0.0217* | -0.0261* | -0.029* | 0.009 |
|  | [-0.0548, -0.0265] | [-0.0523, -0.0241] | [-0.0359, -0.0076] | [-0.0402, -0.012] | [-0.0437, -0.0144] | [-0.0053, 0.0233] |
|  | 1.92×10^-8^ | 1.17×10^-7^ | 0.0026 | 0.0003 | 0.0001 | 0.216 |
| Uniformity | 0.0225* | 0.0174* | 0.0123 | 0.0196* | 0.0205* | -0.0037 |
|  | [0.0084, 0.0366] | [0.0034, 0.0315] | [-0.0018, 0.0264] | [0.0055, 0.0336] | [0.0059, 0.0351] | [-0.0179, 0.0105] |
|  | 0.0018 | 0.0153 | 0.0883 | 0.0063 | 0.0059 | 0.6057 |

**Supplemental Table 13 footnote:** Each cell represents a separate model, adjusted for: age, sex, social deprivation, educational level, smoking, alcohol intake, exercise level. Results are degree of change in radiomics first-order feature per 100g increase in daily meat/fish consumption with corresponding 95% confidence intervals and p-values. First, second, and third row for every CMR measures corresponds to beta coefficient, 95% confidence interval and p-value, respectively. Bonferroni adjusted significance threshold p-value =0.0004 (corrected for 108 comparisons). CMR: cardiovascular magnetic resonance; LV: left ventricle.

**Supplemental Table 11. Multivariate linear regression models showing change in LV myocardium texture radiomics in end-diastole per 100g increase in daily meat/fish consumption**

|  | Unprocessed red meat | Beef | Lamb | Pork | Processed meat | Oily fish |
| --- | --- | --- | --- | --- | --- | --- |
| Autocorrelation (GLCM) | -0.0765 | -0.069 | -0.0518 | -0.0443 | -0.0652 | 0.0178 |
|  | [-0.0905, -0.0625] | [-0.083, -0.055] | [-0.0659, -0.0378] | [-0.0582, -0.0303] | [-0.0797, -0.0507] | [0.0037, 0.032] |
|  | 1.49×10^-26^ | 4.41×10^-22^ | 4.56×10^-13^ | 5.41×10^-10^ | 1.37×10^-18^ | 0.0135 |
| Joint Average (GLCM) | -0.0754 | -0.0677 | -0.0512 | -0.044 | -0.0675 | 0.0151 |
|  | [-0.0894, -0.0614] | [-0.0817, -0.0538] | [-0.0652, -0.0372] | [-0.0579, -0.03] | [-0.082, -0.0531] | [0.001, 0.0293] |
|  | 5.85×10^-26^ | 1.97×10^-21^ | 7.84×10^-13^ | 6.42×10^-10^ | 6.32×10^-20^ | 0.0354 |
| Cluster Prominence (GLCM) | -0.0165 | -0.0173 | -0.004 | -0.0124 | -0.0012 | -0.0032 |
|  | [-0.0309, -0.0021] | [-0.0316, -0.0029] | [-0.0184, 0.0104] | [-0.0267, 0.0019] | [-0.0161, 0.0137] | [-0.0177, 0.0113] |
|  | 0.025 | 0.0183 | 0.587 | 0.0903 | 0.8733 | 0.6676 |
| Cluster Shade (GLCM) | -0.015 | -0.0168 | -0.0013 | -0.0118 | -0.0035 | -0.001 |
|  | [-0.0293, -0.0007] | [-0.0311, -0.0026] | [-0.0156, 0.013] | [-0.026, 0.0024] | [-0.0183, 0.0113] | [-0.0154, 0.0134] |
|  | 0.0405 | 0.0205 | 0.8574 | 0.1046 | 0.6447 | 0.8933 |
| Cluster Tendency (GLCM) | -0.0348 | -0.0332 | -0.0184 | -0.0217 | -0.0191 | -0.0004 |
|  | [-0.0488, -0.0208] | [-0.0471, -0.0192] | [-0.0324, -0.0044] | [-0.0356, -0.0078] | [-0.0336, -0.0046] | [-0.0145, 0.0137] |
|  | 1.14×10^-6^ | 3.23×10^-6^ | 0.0099 | 0.0023 | 0.0097 | 0.9596 |
| Contrast (GLCM) | -0.0438 | -0.0404 | -0.024 | -0.0284 | -0.0335 | -0.0042 |
|  | [-0.057, -0.0307] | [-0.0535, -0.0273] | [-0.0371, -0.0109] | [-0.0415, -0.0154] | [-0.0471, -0.0199] | [-0.0174, 0.009] |
|  | 6.09×10^-11^ | 1.45×10^-9^ | 0.0003 | 1.99×10^-5^ | 1.3×10^-6^ | 0.5321 |
| Correlation (GLCM) | 0.0017 | 0.0028 | -0.0043 | 0.0037 | 0.0064 | 0.0176 |
|  | [-0.012, 0.0155] | [-0.0109, 0.0165] | [-0.018, 0.0095] | [-0.01, 0.0173] | [-0.0078, 0.0206] | [0.0038, 0.0315] |
|  | 0.8053 | 0.6909 | 0.5418 | 0.6001 | 0.3792 | 0.0124 |
| Difference Average (GLCM) | -0.0369 | -0.0322 | -0.0221 | -0.0245 | -0.0321 | -0.012 |
|  | [-0.0495, -0.0242] | [-0.0448, -0.0196] | [-0.0348, -0.0095] | [-0.0371, -0.0119] | [-0.0452, -0.019] | [-0.0247, 0.0007] |
|  | 1.14×10^-8^ | 5.75×10^-7^ | 0.0006 | 0.0001 | 1.52×10^-6^ | 0.0649 |
| Difference Entropy (GLCM) | -0.0441 | -0.04 | -0.0251 | -0.0285 | -0.0361 | -0.01 |
|  | [-0.0566, -0.0317] | [-0.0524, -0.0276] | [-0.0375, -0.0127] | [-0.0408, -0.0161] | [-0.049, -0.0233] | [-0.0225, 0.0025] |
|  | 3.52×10^-12^ | 2.42×10^-10^ | 7.27×10^-5^ | 6.3×10^-6^ | 3.48×10^-8^ | 0.1164 |
| Difference Variance (GLCM) | -0.0487 | -0.0463 | -0.0255 | -0.0307 | -0.0352 | 0.0012 |
|  | [-0.0621, -0.0353] | [-0.0596, -0.0329] | [-0.0389, -0.0121] | [-0.0441, -0.0174] | [-0.0491, -0.0213] | [-0.0123, 0.0148] |
|  | 1.2×10^-12^ | 1.2×10^-11^ | 0.0002 | 6.34×10^-6^ | 6.74×10^-7^ | 0.8562 |
| Joint Energy (GLCM) | 0.0183 | 0.0122 | 0.015 | 0.014 | 0.0206 | 0.0143 |
|  | [0.0046, 0.032] | [-0.0014, 0.0259] | [0.0014, 0.0287] | [0.0004, 0.0277] | [0.0064, 0.0347] | [0.0005, 0.0281] |
|  | 0.0087 | 0.0789 | 0.0309 | 0.0432 | 0.0043 | 0.0416 |
| Joint Entropy (GLCM) | -0.0377 | -0.0325 | -0.024 | -0.0243 | -0.0318 | -0.0103 |
|  | [-0.0509, -0.0245] | [-0.0457, -0.0194] | [-0.0371, -0.0108] | [-0.0375, -0.0112] | [-0.0455, -0.0182] | [-0.0236, 0.003] |
|  | 2.25×10^-8^ | 1.25×10^-6^ | 0.0004 | 0.0003 | 4.75×10^-6^ | 0.1276 |
| Informal Measure Of Correlation 1 (GLCM) | -0.0051 | -0.0024 | -0.0003 | -0.0082 | -0.0075 | -0.0251 |
|  | [-0.0186, 0.0084] | [-0.0159, 0.011] | [-0.0138, 0.0132] | [-0.0216, 0.0052] | [-0.0215, 0.0065] | [-0.0387, -0.0115] |
|  | 0.4611 | 0.7234 | 0.9651 | 0.2317 | 0.2924 | 0.0003 |
| Informal Measure Of Correlation 2 (GLCM) | -0.0199 | -0.0196 | -0.016 | -0.0072 | -0.0103 | 0.0193 |
|  | [-0.0341, -0.0058] | [-0.0337, -0.0055] | [-0.0301, -0.0018] | [-0.0212, 0.0069] | [-0.0249, 0.0044] | [0.0051, 0.0335] |
|  | 0.0058 | 0.0063 | 0.0267 | 0.3178 | 0.1692 | 0.0079 |
| Inverse Difference Moment (GLCM) | 0.0303 | 0.0253 | 0.0192 | 0.0207 | 0.0285 | 0.0155 |
|  | [0.0177, 0.043] | [0.0127, 0.0379] | [0.0066, 0.0319] | [0.0081, 0.0332] | [0.0154, 0.0416] | [0.0028, 0.0283] |
|  | 2.63×10^-6^ | 8.17×10^-5^ | 0.0029 | 0.0013 | 1.94×10^-5^ | 0.0167 |
| Inverse Difference Moment Normalized (GLCM) | -0.0359 | -0.0344 | -0.0214 | -0.0221 | -0.0405 | 0.0226 |
|  | [-0.0499, -0.022] | [-0.0483, -0.0205] | [-0.0354, -0.0074] | [-0.036, -0.0082] | [-0.0549, -0.026] | [0.0085, 0.0366] |
|  | 4.83×10-7 | 1.34×10^-6^ | 0.0027 | 0.0019 | 4.16×10^-8^ | 0.0017 |
| Inverse Difference (GLCM) | 0.029 | 0.0237 | 0.0187 | 0.0201 | 0.0277 | 0.0162 |
|  | [0.0162, 0.0417] | [0.011, 0.0364] | [0.0059, 0.0314] | [0.0074, 0.0328] | [0.0145, 0.0409] | [0.0033, 0.029] |
|  | 8.53×10^-6^ | 0.0002 | 0.0041 | 0.0019 | 3.74×10^-5^ | 0.0135 |
| Inverse Difference Normalized (GLCM) | -0.0268 | -0.0286 | -0.0129 | -0.0152 | -0.0284 | 0.0266 |
|  | [-0.0406, -0.0131] | [-0.0423, -0.0149] | [-0.0266, 0.0008] | [-0.0289, -0.0016] | [-0.0427, -0.0142] | [0.0127, 0.0404] |
|  | 0.0001 | 4.4×10^-5^ | 0.0657 | 0.0291 | 8.8×10^-5^ | 0.0002 |
| Inverse Variance (GLCM) | -0.0099 | -0.0039 | -0.0093 | -0.0101 | -0.0147 | -0.0194 |
|  | [-0.0237, 0.0038] | [-0.0176, 0.0098] | [-0.0231, 0.0044] | [-0.0238, 0.0035] | [-0.0289, -0.0005] | [-0.0332, -0.0056] |
|  | 0.1577 | 0.5777 | 0.1831 | 0.146 | 0.0432 | 0.006 |
| Maximum Probability (GLCM) | 0.0162 | 0.0107 | 0.014 | 0.012 | 0.0201 | 0.0116 |
|  | [0.0023, 0.0301] | [-0.0031, 0.0246] | [0.0001, 0.0279] | [-0.0018, 0.0258] | [0.0057, 0.0345] | [-0.0024, 0.0256] |
|  | 0.0225 | 0.1294 | 0.0479 | 0.0886 | 0.0061 | 0.1044 |
| Sum Average (GLCM) | -0.0754 | -0.0677 | -0.0512 | -0.044 | -0.0675 | 0.0151 |
|  | [-0.0894, -0.0614] | [-0.0817, -0.0538] | [-0.0652, -0.0372] | [-0.0579, -0.03] | [-0.082, -0.0531] | [0.001, 0.0293] |
|  | 5.85×10^-26^ | 1.97×10^-21^ | 7.84×10^-13^ | 6.42×10^-10^ | 6.32×10^-20^ | 0.0354 |
| Sum Entropy (GLCM) | -0.0374 | -0.0321 | -0.0249 | -0.0237 | -0.0313 | -0.0047 |
|  | [-0.051, -0.0239] | [-0.0456, -0.0186] | [-0.0384, -0.0114] | [-0.0372, -0.0102] | [-0.0453, -0.0174] | [-0.0183, 0.0089] |
|  | 6.12×10^-8^ | 3.09×10^-6^ | 0.0003 | 0.0006 | 1.14×10^-5^ | 0.4998 |
| Sum Of Squares (GLCM) | -0.039 | -0.0367 | -0.0209 | -0.0246 | -0.0244 | -0.0016 |
|  | [-0.0528, -0.0252] | [-0.0504, -0.023] | [-0.0346, -0.0071] | [-0.0383, -0.011] | [-0.0386, -0.0102] | [-0.0154, 0.0122] |
|  | 2.8×10^-8^ | 1.52×10^-7^ | 0.0029 | 0.0004 | 0.0008 | 0.8212 |
| Small Area Emphasis (GLSZM) | -0.0343 | -0.0309 | -0.0196 | -0.0237 | -0.0337 | 0.0149 |
|  | [-0.0486, -0.02] | [-0.0451, -0.0167] | [-0.0338, -0.0053] | [-0.0379, -0.0095] | [-0.0484, -0.0189] | [0.0006, 0.0293] |
|  | 2.53×10^-6^ | 2.05×10^-5^ | 0.0071 | 0.0011 | 7.62×10^-6^ | 0.0417 |
| Large Area Emphasis (GLSZM) | 0.0412 | 0.0378 | 0.0248 | 0.0242 | 0.0332 | 0.0181 |
|  | [0.0299, 0.0524] | [0.0266, 0.049] | [0.0135, 0.036] | [0.0131, 0.0354] | [0.0216, 0.0449] | [0.0068, 0.0294] |
|  | 7.63×10^-13^ | 3.77×10^-11^ | 1.56×10^-5^ | 2.17×10^-5^ | 2.13×10^-8^ | 0.0018 |
| Grey Level Non Uniformity (GLSZM) | 0.0164 | 0.0078 | 0.0132 | 0.0175 | 0.0188 | 0.0125 |
|  | [0.0024, 0.0303] | [-0.0061, 0.0217] | [-0.0008, 0.0271] | [0.0037, 0.0314] | [0.0043, 0.0332] | [-0.0015, 0.0266] |
|  | 0.0216 | 0.2712 | 0.064 | 0.0132 | 0.0108 | 0.0802 |
| Size Zone Non Uniformity (GLSZM) | 0.0164 | 0.0078 | 0.0132 | 0.0175 | 0.0188 | 0.0125 |
|  | [0.0024, 0.0303] | [-0.0061, 0.0217] | [-0.0008, 0.0271] | [0.0037, 0.0314] | [0.0043, 0.0332] | [-0.0015, 0.0266] |
|  | 0.0216 | 0.2712 | 0.064 | 0.0132 | 0.0108 | 0.0802 |
| Size Zone Non Uniformity Normalized (GLSZM) | -0.036 | -0.0316 | -0.0216 | -0.0252 | -0.0348 | 0.0143 |
|  | [-0.0502, -0.0217] | [-0.0458, -0.0174] | [-0.0359, -0.0074] | [-0.0394, -0.011] | [-0.0495, -0.02] | [0, 0.0287] |
|  | 8.09×10^-7^ | 1.34×10^-5^ | 0.0029 | 0.0005 | 3.83×10^-6^ | 0.0507 |
| Zone Percentage (GLSZM) | -0.0574 | -0.0537 | -0.0347 | -0.0333 | -0.0417 | 0.0009 |
|  | [-0.0702, -0.0447] | [-0.0665, -0.041] | [-0.0474, -0.022] | [-0.046, -0.0206] | [-0.0549, -0.0285] | [-0.0119, 0.0138] |
|  | 1.23×10^-18^ | 1.23×10^-16^ | 9.69×10^-8^ | 2.82×10^-7^ | 6.22×10^-10^ | 0.8865 |
| Grey Level Variance (GLSZM) | -0.0627 | -0.0613 | -0.0333 | -0.0373 | -0.0544 | 0.0074 |
|  | [-0.0768, -0.0485] | [-0.0754, -0.0472] | [-0.0475, -0.0192] | [-0.0514, -0.0232] | [-0.0691, -0.0398] | [-0.0069, 0.0217] |
|  | 4.56×10^-18^ | 1.75×10^-17^ | 3.96×10^-6^ | 2.16×10^-7^ | 3.19×10^-13^ | 0.3085 |
| Zone Variance (GLSZM) | 0.041 | 0.0375 | 0.0248 | 0.0242 | 0.033 | 0.0183 |
|  | [0.0298, 0.0522] | [0.0264, 0.0487] | [0.0135, 0.036] | [0.0131, 0.0354] | [0.0214, 0.0446] | [0.007, 0.0296] |
|  | 8.87×10^-13^ | 4.97×10^-11^ | 1.54×10^-5^ | 2.13×10^-5^ | 2.57×10^-8^ | 0.0015 |
| Zone Entropy (GLSZM) | -0.037 | -0.0414 | -0.0174 | -0.0149 | -0.0388 | 0.0073 |
|  | [-0.0512, -0.0229] | [-0.0555, -0.0273] | [-0.0315, -0.0033] | [-0.0289, -0.0008] | [-0.0534, -0.0242] | [-0.007, 0.0215] |
|  | 3×10^-7^ | 8.83×10^-9^ | 0.0158 | 0.0385 | 1.99×10^-7^ | 0.3162 |
| Low Grey Level Zone Emphasis (GLSZM) | 0.0645 | 0.0559 | 0.0458 | 0.0396 | 0.0575 | -0.0245 |
|  | [0.0504, 0.0786] | [0.0418, 0.0699] | [0.0317, 0.0599] | [0.0255, 0.0536] | [0.0429, 0.0721] | [-0.0388, -0.0103] |
|  | 4.04×10^-19^ | 7.9×10^-15^ | 2.14×10^-10^ | 3.53×10^-8^ | 1.26×10^-14^ | 0.0007 |
| High Grey Level Zone Emphasis (GLSZM) | -0.0733 | -0.0684 | -0.0447 | -0.044 | -0.0652 | 0.0148 |
|  | [-0.0873, -0.0592] | [-0.0824, -0.0543] | [-0.0588, -0.0306] | [-0.058, -0.03] | [-0.0798, -0.0507] | [0.0006, 0.029] |
|  | 2.14×10^-24^ | 1.33×10^-21^ | 4.8×10^-10^ | 7.59×10^-10^ | 1.64×10^-18^ | 0.0405 |
| Small Area Low Grey Level Emphasis (GLSZM) | 0.0581 | 0.05 | 0.0423 | 0.0352 | 0.0518 | -0.0224 |
|  | [0.044, 0.0723] | [0.0359, 0.064] | [0.0282, 0.0565] | [0.0212, 0.0493] | [0.0372, 0.0664] | [-0.0366, -0.0082] |
|  | 8.08×10^-16^ | 3.64×10^-12^ | 4.13×10^-9^ | 9.11×10-7 | 3.82×10^-12^ | 0.002 |
| Small Area High Grey Level Emphasis (GLSZM) | -0.0738 | -0.0689 | -0.0449 | -0.0443 | -0.0673 | 0.0152 |
|  | [-0.0879, -0.0597] | [-0.083, -0.0549] | [-0.0589, -0.0308] | [-0.0583, -0.0303] | [-0.0818, -0.0528] | [0.001, 0.0294] |
|  | 9.86×10^-25^ | 6.07×10^-22^ | 4.11×10^-10^ | 5.75×10^-10^ | 1.28×10^-19^ | 0.0358 |
| Large Area Low Grey Level Emphasis (GLSZM) | 0.0455 | 0.0407 | 0.0303 | 0.0266 | 0.0549 | 0.0182 |
|  | [0.0318, 0.0592] | [0.027, 0.0544] | [0.0166, 0.044] | [0.013, 0.0403] | [0.0407, 0.069] | [0.0044, 0.0321] |
|  | 8.54×10^-11^ | 5.52×10^-9^ | 1.5×10^-5^ | 0.0001 | 3.35×10^-14^ | 0.0096 |
| Large Area High Grey Level Emphasis (GLSZM) | 0.0013 | 0.0034 | -0.0051 | 0.0014 | -0.0046 | 0.024 |
|  | [-0.0104, 0.0129] | [-0.0082, 0.015] | [-0.0167, 0.0065] | [-0.0101, 0.013] | [-0.0166, 0.0075] | [0.0123, 0.0357] |
|  | 0.8328 | 0.5628 | 0.3892 | 0.8068 | 0.4584 | 5.98×10^-5^ |
| Short Run Emphasis (GLRLM) | -0.0299 | -0.0236 | -0.0226 | -0.0189 | -0.0266 | -0.0152 |
|  | [-0.0419, -0.0178] | [-0.0356, -0.0116] | [-0.0347, -0.0106] | [-0.0309, -0.007] | [-0.0391, -0.0142] | [-0.0273, -0.0031] |
|  | 1.2×10^-6^ | 0.0001 | 0.0002 | 0.0019 | 2.76×10^-5^ | 0.0141 |
| Long Run Emphasis (GLRLM) | 0.0311 | 0.0238 | 0.0222 | 0.0221 | 0.0278 | 0.0185 |
|  | [0.0188, 0.0434] | [0.0116, 0.036] | [0.0099, 0.0345] | [0.0099, 0.0343] | [0.0151, 0.0405] | [0.0061, 0.0308] |
|  | 7.18×10^-7^ | 0.0001 | 0.0004 | 0.0004 | 1.83×10^-5^ | 0.0034 |
| Grey Level Non Uniformity (GLRLM) | 0.035 | 0.0278 | 0.0213 | 0.0273 | 0.0249 | 0.0279 |
|  | [0.0248, 0.0453] | [0.0176, 0.038] | [0.011, 0.0315] | [0.0171, 0.0375] | [0.0143, 0.0355] | [0.0176, 0.0383] |
|  | 2.23×10^-11^ | 1.03×10^-7^ | 4.8×10^-5^ | 1.58×10^-7^ | 4.28×10^-6^ | 1.12×10^-7^ |
| Run Length Non Uniformity (GLRLM) | 0.0173 | 0.0106 | 0.0112 | 0.0182 | 0.0067 | 0.0201 |
|  | [0.0046, 0.0301] | [-0.0021, 0.0234] | [-0.0016, 0.0239] | [0.0055, 0.0309] | [-0.0065, 0.0199] | [0.0073, 0.033] |
|  | 0.0077 | 0.1008 | 0.086 | 0.0049 | 0.3219 | 0.0021 |
| Run Length Non Uniformity Normalized (GLRLM) | -0.0279 | -0.0222 | -0.0216 | -0.0168 | -0.0246 | -0.0138 |
|  | [-0.04, -0.0157] | [-0.0343, -0.0101] | [-0.0338, -0.0095] | [-0.0289, -0.0048] | [-0.0372, -0.0121] | [-0.0261, -0.0016] |
|  | 7.13×10^-6^ | 0.0003 | 0.0005 | 0.0063 | 0.0001 | 0.0268 |
| Run Percentage (GLRLM) | -0.0267 | -0.0205 | -0.0207 | -0.0172 | -0.0236 | -0.0143 |
|  | [-0.0389, -0.0144] | [-0.0326, -0.0083] | [-0.0329, -0.0085] | [-0.0293, -0.005] | [-0.0362, -0.011] | [-0.0266, -0.002] |
|  | 1.91×10^-5^ | 0.001 | 0.0009 | 0.0056 | 0.0002 | 0.0223 |
| Grey Level Variance (GLRLM) | -0.0453 | -0.0442 | -0.0221 | -0.0285 | -0.0301 | 0.0005 |
|  | [-0.0591, -0.0315] | [-0.0579, -0.0304] | [-0.0359, -0.0083] | [-0.0423, -0.0148] | [-0.0444, -0.0158] | [-0.0135, 0.0144] |
|  | 1.43×10^-10^ | 3.5×10^-10^ | 0.0017 | 4.89×10^-5^ | 3.8×10^-5^ | 0.948 |
| Run Variance (GLRLM) | 0.0298 | 0.0227 | 0.0211 | 0.0214 | 0.0261 | 0.0191 |
|  | [0.0173, 0.0422] | [0.0103, 0.0351] | [0.0087, 0.0335] | [0.0091, 0.0338] | [0.0133, 0.039] | [0.0066, 0.0317] |
|  | 2.75×10^-6^ | 0.0003 | 0.0009 | 0.0007 | 6.71×10^-5^ | 0.0027 |
| Run Entropy (GLRLM) | -0.025 | -0.0286 | -0.0065 | -0.0154 | -0.0206 | 0.014 |
|  | [-0.0388, -0.0111] | [-0.0424, -0.0148] | [-0.0204, 0.0073] | [-0.0292, -0.0017] | [-0.0349, -0.0063] | [0.0001, 0.028] |
|  | 0.0004 | 4.93×10^-5^ | 0.3557 | 0.0282 | 0.0048 | 0.0485 |
| Low Grey Level Run Emphasis (GLRLM) | 0.0573 | 0.0523 | 0.0387 | 0.0327 | 0.0628 | -0.0016 |
|  | [0.0434, 0.0713] | [0.0385, 0.0662] | [0.0248, 0.0526] | [0.0188, 0.0465] | [0.0484, 0.0772] | [-0.0157, 0.0124] |
|  | 7.92×10^-16^ | 1.5×10^-13^ | 5.01×10^-8^ | 3.85×10^-6^ | 1.26×10^-17^ | 0.8202 |
| High Grey Level Run Emphasis (GLRLM) | -0.0768 | -0.0702 | -0.0506 | -0.0444 | -0.066 | 0.0164 |
|  | [-0.0909, -0.0627] | [-0.0842, -0.0562] | [-0.0647, -0.0366] | [-0.0585, -0.0304] | [-0.0805, -0.0514] | [0.0022, 0.0306] |
|  | 1.38×10^-26^ | 1.22×10^-22^ | 1.85×10^-12^ | 5.28×10^-10^ | 7.22×10^-19^ | 0.0236 |
| Short Run Low Grey Level Emphasis (GLRLM) | 0.0558 | 0.0515 | 0.037 | 0.0317 | 0.0604 | -0.0047 |
|  | [0.0419, 0.0696] | [0.0377, 0.0653] | [0.0232, 0.0509] | [0.0179, 0.0454] | [0.0461, 0.0747] | [-0.0186, 0.0093] |
|  | 2.95×10^-15^ | 2.6×10^-13^ | 1.51×10-7 | 6.54×10^-6^ | 1.29×10^-16^ | 0.511 |
| Short Run High Grey Level Emphasis (GLRLM) | -0.0779 | -0.071 | -0.0509 | -0.0457 | -0.0666 | 0.013 |
|  | [-0.092, -0.0638] | [-0.085, -0.057] | [-0.065, -0.0368] | [-0.0597, -0.0317] | [-0.0811, -0.052] | [-0.0011, 0.0272] |
|  | 2.39×10^-27^ | 3.81×10^-23^ | 1.37×10^-12^ | 1.68×10^-10^ | 3.22×10^-19^ | 0.0714 |
| Long Run Low Grey Level Emphasis (GLRLM) | 0.0542 | 0.0473 | 0.0379 | 0.0328 | 0.0629 | 0.0088 |
|  | [0.0401, 0.0683] | [0.0332, 0.0613] | [0.0238, 0.0519] | [0.0188, 0.0468] | [0.0483, 0.0775] | [-0.0054, 0.023] |
|  | 5.1×10^-14^ | 4.27×10^-11^ | 1.37×10^-7^ | 4.49×10^-6^ | 2.64×10^-17^ | 0.2234 |
| Long Run High Grey Level Emphasis (GLRLM) | -0.0497 | -0.047 | -0.0341 | -0.0257 | -0.0437 | 0.027 |
|  | [-0.0629, -0.0365] | [-0.0601, -0.0339] | [-0.0473, -0.021] | [-0.0388, -0.0126] | [-0.0573, -0.0301] | [0.0137, 0.0402] |
|  | 1.62×10^-13^ | 2.36×10^-12^ | 3.8×10^-7^ | 0.0001 | 3.3×10^-10^ | 6.8×10^-5^ |
| Coarseness (NGTDM) | -0.0288 | -0.0259 | -0.0181 | -0.0166 | -0.0096 | -0.0183 |
|  | [-0.0401, -0.0174] | [-0.0373, -0.0146] | [-0.0295, -0.0068] | [-0.0279, -0.0053] | [-0.0214, 0.0021] | [-0.0298, -0.0069] |
|  | 7.04×10^-7^ | 7.02×10^-6^ | 0.0017 | 0.004 | 0.108 | 0.0017 |
| Contrast (NGTDM) | 0.0242 | 0.0216 | 0.0167 | 0.0157 | 0.0303 | -0.0216 |
|  | [0.0104, 0.038] | [0.0079, 0.0353] | [0.0029, 0.0304] | [0.002, 0.0294] | [0.0161, 0.0446] | [-0.0354, -0.0077] |
|  | 0.0006 | 0.002 | 0.0174 | 0.0247 | 2.97×10^-5^ | 0.0022 |
| Busyness (NGTDM) | 0.0573 | 0.0495 | 0.0386 | 0.0369 | 0.064 | 0.001 |
|  | [0.0433, 0.0713] | [0.0356, 0.0635] | [0.0246, 0.0526] | [0.023, 0.0508] | [0.0496, 0.0785] | [-0.0131, 0.0152] |
|  | 1.16×10^-15^ | 3.69×10^-12^ | 6.47×10^-8^ | 2.09×10^-7^ | 4.46×10^-18^ | 0.884 |
| Complexity (NGTDM) | -0.0468 | -0.0458 | -0.0229 | -0.0297 | -0.0431 | 0.0109 |
|  | [-0.0608, -0.0328] | [-0.0597, -0.0319] | [-0.0369, -0.009] | [-0.0436, -0.0159] | [-0.0576, -0.0287] | [-0.0031, 0.025] |
|  | 5.32×10^-11^ | 1.1×10^-10^ | 0.0013 | 2.7×10^-5^ | 4.71×10^-9^ | 0.1279 |
| Strength (NGTDM) | -0.0373 | -0.0387 | -0.0148 | -0.0239 | -0.0267 | 0.0089 |
|  | [-0.0512, -0.0235] | [-0.0525, -0.0249] | [-0.0287, -0.0009] | [-0.0377, -0.0101] | [-0.041, -0.0123] | [-0.0051, 0.0228] |
|  | 1.38×10^-7^ | 4.3×10^-8^ | 0.0364 | 0.0007 | 0.0003 | 0.2136 |
| Small Dependence Emphasis (GLDM) | -0.0554 | -0.0509 | -0.0344 | -0.0325 | -0.0432 | -0.0025 |
|  | [-0.0678, -0.0431] | [-0.0632, -0.0386] | [-0.0468, -0.0221] | [-0.0448, -0.0203] | [-0.056, -0.0305] | [-0.0149, 0.01] |
|  | 1.42×10^-18^ | 4.87×10^-16^ | 4.36×10^-8^ | 2.06×10^-7^ | 3.19×10^-11^ | 0.699 |
| Large Dependence Emphasis (GLDM) | 0.0241 | 0.0175 | 0.0194 | 0.0164 | 0.0216 | 0.0146 |
|  | [0.0117, 0.0366] | [0.0051, 0.0299] | [0.007, 0.0318] | [0.004, 0.0287] | [0.0087, 0.0344] | [0.0021, 0.0271] |
|  | 0.0001 | 0.0057 | 0.0022 | 0.0094 | 0.001 | 0.0222 |
| Grey Level Non Uniformity (GLDM) | 0.0335 | 0.0253 | 0.023 | 0.0252 | 0.0251 | 0.028 |
|  | [0.0229, 0.0441] | [0.0148, 0.0359] | [0.0124, 0.0336] | [0.0147, 0.0358] | [0.0142, 0.0361] | [0.0173, 0.0387] |
|  | 6.42×10^-10^ | 2.7×10^-6^ | 2.05×10^-5^ | 2.83×10^-6^ | 6.99×10^-6^ | 2.69×10^-7^ |
| Dependence Non Uniformity (GLDM) | 0.0215 | 0.0158 | 0.013 | 0.0195 | 0.0114 | 0.0197 |
|  | [0.0088, 0.0342] | [0.0032, 0.0285] | [0.0003, 0.0257] | [0.0068, 0.0321] | [-0.0017, 0.0245] | [0.007, 0.0325] |
|  | 0.0009 | 0.0142 | 0.0445 | 0.0025 | 0.089 | 0.0024 |
| Dependence Non Uniformity Normalized (GLDM) | -0.019 | -0.013 | -0.0165 | -0.0126 | -0.016 | -0.0127 |
|  | [-0.0318, -0.0062] | [-0.0258, -0.0003] | [-0.0292, -0.0037] | [-0.0253, 0.0001] | [-0.0292, -0.0028] | [-0.0256, 0.0002] |
|  | 0.0036 | 0.0446 | 0.0114 | 0.0524 | 0.0176 | 0.0529 |
| Grey Level Variance (GLDM) | -0.0397 | -0.0383 | -0.02 | -0.025 | -0.0258 | -0.0028 |
|  | [-0.0535, -0.026] | [-0.052, -0.0246] | [-0.0337, -0.0063] | [-0.0387, -0.0114] | [-0.04, -0.0116] | [-0.0166, 0.0111] |
|  | 1.44×10^-8^ | 4.06×10^-8^ | 0.0043 | 0.0003 | 0.0004 | 0.6961 |
| Dependence Variance (GLDM) | 0.0166 | 0.0092 | 0.0153 | 0.0136 | 0.0138 | 0.0117 |
|  | [0.0035, 0.0297] | [-0.0039, 0.0222] | [0.0022, 0.0284] | [0.0006, 0.0266] | [0.0003, 0.0273] | [-0.0014, 0.0249] |
|  | 0.0128 | 0.1684 | 0.0216 | 0.0401 | 0.0458 | 0.0804 |
| Dependence Entropy (GLDM) | 2.99×10^-6^ | -0.004 | 0.0023 | 0.003 | -0.006 | 0.0077 |
|  | [-0.0136, 0.0136] | [-0.0175, 0.0095] | [-0.0113, 0.0158] | [-0.0105, 0.0165] | [-0.0201, 0.008] | [-0.006, 0.0213] |
|  | 0.9997 | 0.5607 | 0.7444 | 0.6672 | 0.3994 | 0.2693 |
| Low Grey Level Emphasis (GLDM) | 0.0558 | 0.0509 | 0.0377 | 0.032 | 0.0621 | -0.0001 |
|  | [0.0419, 0.0697] | [0.037, 0.0647] | [0.0238, 0.0515] | [0.0182, 0.0458] | [0.0477, 0.0764] | [-0.0141, 0.0139] |
|  | 3.77×10^-15^ | 6.06×10^-13^ | 1.06×10^-7^ | 5.72×10^-6^ | 2.49×10^-17^ | 0.9882 |
| High Grey Level Emphasis (GLDM) | -0.0766 | -0.0696 | -0.0512 | -0.0443 | -0.0656 | 0.0163 |
|  | [-0.0907, -0.0625] | [-0.0836, -0.0555] | [-0.0653, -0.0371] | [-0.0583, -0.0303] | [-0.0801, -0.051] | [0.0021, 0.0304] |
|  | 1.82×10^-26^ | 2.73×10^-22^ | 1.04×10^-12^ | 6.11×10^-10^ | 1.13×10^-18^ | 0.0247 |
| Small Dependence Low Grey Level Emphasis (GLDM) | 0.0332 | 0.0288 | 0.0244 | 0.0203 | 0.0349 | -0.0187 |
|  | [0.0199, 0.0465] | [0.0155, 0.0421] | [0.0111, 0.0377] | [0.007, 0.0335] | [0.0212, 0.0487] | [-0.0321, -0.0053] |
|  | 1.07×10^-6^ | 2.18×10^-5^ | 0.0003 | 0.0027 | 6.65×10^-7^ | 0.0064 |
| Small Dependence High Grey Level Emphasis (GLDM) | -0.0725 | -0.0669 | -0.0446 | -0.0437 | -0.0586 | 0.0091 |
|  | [-0.0863, -0.0588] | [-0.0806, -0.0532] | [-0.0584, -0.0309] | [-0.0574, -0.03] | [-0.0728, -0.0444] | [-0.0047, 0.023] |
|  | 5.18×10^-25^ | 1.18×10^-21^ | 1.96×10^-10^ | 3.98×10^-10^ | 6.79×10^-16^ | 0.1958 |
| Large Dependence Low Grey Level Emphasis (GLDM) | 0.0523 | 0.0452 | 0.0379 | 0.031 | 0.0591 | 0.009 |
|  | [0.0382, 0.0664] | [0.0312, 0.0592] | [0.0239, 0.052] | [0.017, 0.045] | [0.0446, 0.0737] | [-0.0052, 0.0232] |
|  | 3.54×10^-13^ | 2.84×10^-10^ | 1.24×10^-7^ | 1.44×10^-5^ | 1.73×10^-15^ | 0.2127 |
| Large Dependence High Grey Level Emphasis (GLDM) | -0.0439 | -0.0421 | -0.03 | -0.0223 | -0.0379 | 0.0253 |
|  | [-0.0569, -0.031] | [-0.055, -0.0292] | [-0.0429, -0.0171] | [-0.0351, -0.0094] | [-0.0512, -0.0245] | [0.0123, 0.0383] |
|  | 2.78×10^-11^ | 1.46×10^-10^ | 5.25×10^-6^ | 0.0007 | 2.76×10^-8^ | 0.0001 |

**Supplemental Table 11 footnote:** Each cell represents a separate model, adjusted for: age, sex, social deprivation, educational level, smoking, alcohol intake, exercise level. Results are degree of change in radiomics shape feature per 100g increase in daily meat/fish consumption with corresponding 95% confidence intervals and p-values. First, second, and third row for every CMR measures corresponds to beta coefficient, 95% confidence interval and p-value, respectively. Bonferroni adjusted significance threshold p-value =0.0001 (corrected for 432 comparisons). GLCM: grey level co-occurrence matrix; GLDM: grey level dependence matrix; GLRLM: grey level run length matrix; GLSZM: grey level size zone matrix; NGTDM: neighboring grey tone difference matrix; LV: left ventricle.

**Supplemental Table 12. Multivariate linear regression models showing change in LV myocardium texture radiomics in end-systole per 100g increase in daily meat/fish consumption**

|  | Unprocessed red meat | Beef | Lamb | Pork | Processed meat | Oily fish |
| --- | --- | --- | --- | --- | --- | --- |
| Autocorrelation (GLCM) | -0.0763 | -0.0729 | -0.045 | -0.0442 | -0.0703 | 0.0103 |
|  | [-0.0901, -0.0624] | [-0.0867, -0.0591] | [-0.0588, -0.0311] | [-0.058, -0.0305] | [-0.0846, -0.0559] | [-0.0037, 0.0242] |
|  | 4.23×10^-27^ | 4.67×10^-25^ | 1.95×10^-10^ | 3.22×10^-10^ | 7.23×10^-22^ | 0.1487 |
| Joint Average (GLCM) | -0.0755 | -0.0721 | -0.0445 | -0.0438 | -0.0709 | 0.0074 |
|  | [-0.0894, -0.0617] | [-0.0859, -0.0583] | [-0.0584, -0.0307] | [-0.0576, -0.03] | [-0.0852, -0.0566] | [-0.0065, 0.0214] |
|  | 1.41×10^-26^ | 1.37×10^-24^ | 2.99×10^-10^ | 4.81×10^-10^ | 2.93×10^-22^ | 0.2973 |
| Cluster Prominence (GLCM) | -0.0112 | -0.0123 | -0.0013 | -0.009 | 0.0012 | -0.001 |
|  | [-0.0257, 0.0032] | [-0.0267, 0.0021] | [-0.0157, 0.0131] | [-0.0233, 0.0054] | [-0.0137, 0.0161] | [-0.0155, 0.0135] |
|  | 0.1272 | 0.0934 | 0.8603 | 0.2198 | 0.876 | 0.8934 |
| Cluster Shade (GLCM) | -0.0066 | -0.0089 | 0.0008 | -0.0044 | 0.0055 | 0.0013 |
|  | [-0.021, 0.0078] | [-0.0233, 0.0054] | [-0.0135, 0.0152] | [-0.0187, 0.0099] | [-0.0094, 0.0203] | [-0.0132, 0.0158] |
|  | 0.3696 | 0.223 | 0.9084 | 0.5456 | 0.4725 | 0.8612 |
| Cluster Tendency (GLCM) | -0.0274 | -0.026 | -0.0142 | -0.0176 | -0.0175 | 0.0117 |
|  | [-0.0416, -0.0132] | [-0.0402, -0.0119] | [-0.0283, 0] | [-0.0317, -0.0035] | [-0.0322, -0.0029] | [-0.0026, 0.026] |
|  | 0.0002 | 0.0003 | 0.0503 | 0.0147 | 0.0192 | 0.109 |
| Contrast (GLCM) | -0.031 | -0.0285 | -0.0156 | -0.021 | -0.0213 | -0.0032 |
|  | [-0.045, -0.017] | [-0.0424, -0.0145] | [-0.0296, -0.0017] | [-0.0349, -0.0071] | [-0.0358, -0.0069] | [-0.0172, 0.0109] |
|  | 1.38×10^-5^ | 6.16×10^-5^ | 0.028 | 0.003 | 0.0038 | 0.6572 |
| Correlation (GLCM) | -0.0052 | -0.0024 | -0.0079 | -0.0035 | -0.0043 | 0.0232 |
|  | [-0.0185, 0.0081] | [-0.0156, 0.0109] | [-0.0212, 0.0054] | [-0.0167, 0.0097] | [-0.0181, 0.0094] | [0.0099, 0.0366] |
|  | 0.444 | 0.7267 | 0.2427 | 0.6022 | 0.5356 | 0.0007 |
| Difference Average (GLCM) | -0.0213 | -0.0199 | -0.0098 | -0.0143 | -0.0164 | -0.0036 |
|  | [-0.0351, -0.0075] | [-0.0336, -0.0062] | [-0.0235, 0.004] | [-0.028, -0.0006] | [-0.0306, -0.0021] | [-0.0174, 0.0103] |
|  | 0.0025 | 0.0045 | 0.164 | 0.041 | 0.0244 | 0.6155 |
| Difference Entropy (GLCM) | -0.0297 | -0.0279 | -0.0143 | -0.0195 | -0.0247 | -0.0033 |
|  | [-0.0434, -0.016] | [-0.0415, -0.0142] | [-0.028, -0.0006] | [-0.0331, -0.0059] | [-0.0388, -0.0105] | [-0.017, 0.0105] |
|  | 2.23×10^-5^ | 6.3×10^-5^ | 0.0406 | 0.0051 | 0.0006 | 0.6437 |
| Difference Variance (GLCM) | -0.0376 | -0.0346 | -0.0192 | -0.0254 | -0.0255 | -0.002 |
|  | [-0.0516, -0.0235] | [-0.0486, -0.0207] | [-0.0333, -0.0052] | [-0.0393, -0.0114] | [-0.04, -0.011] | [-0.0161, 0.0121] |
|  | 1.6×10^-7^ | 1.22×10^-6^ | 0.0072 | 0.0004 | 0.0006 | 0.7819 |
| Joint Energy (GLCM) | 0.0136 | 0.0098 | 0.0073 | 0.0129 | 0.0126 | -0.0011 |
|  | [-0.0006, 0.0278] | [-0.0044, 0.0239] | [-0.0068, 0.0215] | [-0.0012, 0.0269] | [-0.0021, 0.0272] | [-0.0154, 0.0131] |
|  | 0.0598 | 0.1752 | 0.3097 | 0.0732 | 0.0923 | 0.8748 |
| Joint Entropy (GLCM) | -0.0247 | -0.0218 | -0.0131 | -0.0176 | -0.0217 | 0.0032 |
|  | [-0.0388, -0.0107] | [-0.0358, -0.0078] | [-0.0271, 0.001] | [-0.0316, -0.0036] | [-0.0363, -0.0072] | [-0.0109, 0.0174] |
|  | 0.0006 | 0.0023 | 0.0681 | 0.0136 | 0.0035 | 0.656 |
| Informal Measure Of Correlation 1 (GLCM) | 0.0142 | 0.0117 | 0.011 | 0.0098 | 0.0108 | -0.0216 |
|  | [0.0011, 0.0273] | [-0.0014, 0.0248] | [-0.0021, 0.0241] | [-0.0032, 0.0229] | [-0.0027, 0.0244] | [-0.0348, -0.0085] |
|  | 0.0338 | 0.0794 | 0.1005 | 0.1385 | 0.1172 | 0.0013 |
| Informal Measure Of Correlation 2 (GLCM) | -0.0196 | -0.0149 | -0.016 | -0.0138 | -0.0151 | 0.0195 |
|  | [-0.033, -0.0061] | [-0.0283, -0.0016] | [-0.0294, -0.0026] | [-0.0272, -0.0005] | [-0.0289, -0.0012] | [0.006, 0.033] |
|  | 0.0043 | 0.0285 | 0.0191 | 0.0421 | 0.0333 | 0.0047 |
| Inverse Difference Moment (GLCM) | 0.0164 | 0.0154 | 0.0069 | 0.011 | 0.0133 | 0.0037 |
|  | [0.0026, 0.0301] | [0.0017, 0.0292] | [-0.0069, 0.0206] | [-0.0027, 0.0247] | [-0.0009, 0.0275] | [-0.0101, 0.0176] |
|  | 0.0198 | 0.0272 | 0.3254 | 0.1141 | 0.0669 | 0.5976 |
| Inverse Difference Moment Normalized (GLCM) | -0.0414 | -0.0371 | -0.0266 | -0.0268 | -0.0497 | 0.0174 |
|  | [-0.0553, -0.0275] | [-0.051, -0.0233] | [-0.0404, -0.0127] | [-0.0406, -0.013] | [-0.0641, -0.0354] | [0.0034, 0.0314] |
|  | 5.17×10^-9^ | 1.44×10^-7^ | 0.0002 | 0.0001 | 1.11×10^-11^ | 0.0146 |
| Inverse Difference (GLCM) | 0.0144 | 0.0135 | 0.0058 | 0.0098 | 0.0119 | 0.0038 |
|  | [0.0006, 0.0282] | [-0.0002, 0.0273] | [-0.008, 0.0195] | [-0.0039, 0.0235] | [-0.0024, 0.0261] | [-0.01, 0.0177] |
|  | 0.0408 | 0.053 | 0.4092 | 0.159 | 0.1017 | 0.5876 |
| Inverse Difference Normalized (GLCM) | -0.0313 | -0.0285 | -0.0208 | -0.0194 | -0.0358 | 0.0151 |
|  | [-0.0451, -0.0176] | [-0.0423, -0.0148] | [-0.0345, -0.007] | [-0.0332, -0.0057] | [-0.0501, -0.0216] | [0.0012, 0.0289] |
|  | 8.48×10^-6^ | 4.68×10^-5^ | 0.0031 | 0.0054 | 8.25×10^-7^ | 0.033 |
| Inverse Variance (GLCM) | -0.0013 | -0.0011 | 0.0014 | -0.0018 | -0.0026 | -0.0042 |
|  | [-0.0152, 0.0126] | [-0.0149, 0.0127] | [-0.0124, 0.0152] | [-0.0156, 0.012] | [-0.017, 0.0117] | [-0.0182, 0.0097] |
|  | 0.8549 | 0.8724 | 0.8435 | 0.7951 | 0.7193 | 0.5522 |
| Maximum Probability (GLCM) | 0.0125 | 0.0069 | 0.008 | 0.0137 | 0.0089 | 0.0003 |
|  | [-0.0017, 0.0267] | [-0.0072, 0.0211] | [-0.0061, 0.0222] | [-0.0004, 0.0278] | [-0.0058, 0.0236] | [-0.0139, 0.0146] |
|  | 0.0842 | 0.3386 | 0.2663 | 0.0567 | 0.2341 | 0.9634 |
| Sum Average (GLCM) | -0.0755 | -0.0721 | -0.0445 | -0.0438 | -0.0709 | 0.0074 |
|  | [-0.0894, -0.0617] | [-0.0859, -0.0583] | [-0.0584, -0.0307] | [-0.0576, -0.03] | [-0.0852, -0.0566] | [-0.0065, 0.0214] |
|  | 1.41×10^-26^ | 1.37×10^-24^ | 2.99×10^-10^ | 4.81×10^-10^ | 2.93×10^-22^ | 0.2973 |
| Sum Entropy (GLCM) | -0.0237 | -0.0202 | -0.0134 | -0.0174 | -0.0211 | 0.008 |
|  | [-0.0378, -0.0096] | [-0.0343, -0.0062] | [-0.0275, 0.0007] | [-0.0314, -0.0033] | [-0.0357, -0.0065] | [-0.0062, 0.0223] |
|  | 0.001 | 0.0048 | 0.062 | 0.0153 | 0.0046 | 0.2666 |
| Sum Of Squares (GLCM) | -0.0298 | -0.028 | -0.0153 | -0.0194 | -0.0194 | 0.0087 |
|  | [-0.044, -0.0155] | [-0.0422, -0.0139] | [-0.0295, -0.0011] | [-0.0335, -0.0052] | [-0.0341, -0.0047] | [-0.0056, 0.023] |
|  | 4.15×10^-5^ | 0.0001 | 0.0349 | 0.0073 | 0.0097 | 0.2333 |
| Small Area Emphasis (GLSZM) | -0.0212 | -0.022 | -0.0151 | -0.0075 | -0.0016 | 0.0076 |
|  | [-0.0354, -0.0069] | [-0.0362, -0.0078] | [-0.0293, -0.0008] | [-0.0216, 0.0067] | [-0.0164, 0.0131] | [-0.0068, 0.0219] |
|  | 0.0037 | 0.0024 | 0.038 | 0.3022 | 0.8284 | 0.2999 |
| Large Area Emphasis (GLSZM) | 0.0412 | 0.0331 | 0.0265 | 0.0295 | 0.0387 | 0.0201 |
|  | [0.0289, 0.0534] | [0.0209, 0.0453] | [0.0143, 0.0388] | [0.0173, 0.0416] | [0.026, 0.0514] | [0.0078, 0.0324] |
|  | 4.5×10^-11^ | 1.06×10^-7^ | 2.15×10^-5^ | 2.12×10^-6^ | 2.09×10^-9^ | 0.0014 |
| Grey Level Non Uniformity (GLSZM) | 0.0356 | 0.0263 | 0.0255 | 0.0291 | 0.019 | -0.0009 |
|  | [0.0217, 0.0495] | [0.0124, 0.0401] | [0.0117, 0.0394] | [0.0152, 0.0429] | [0.0047, 0.0334] | [-0.0149, 0.013] |
|  | 5.38×10^-7^ | 0.0002 | 0.0003 | 3.74×10^-5^ | 0.0094 | 0.8948 |
| Size Zone Non Uniformity (GLSZM) | 0.0356 | 0.0263 | 0.0255 | 0.0291 | 0.019 | -0.0009 |
|  | [0.0217, 0.0495] | [0.0124, 0.0401] | [0.0117, 0.0394] | [0.0152, 0.0429] | [0.0047, 0.0334] | [-0.0149, 0.013] |
|  | 5.38×10^-7^ | 0.0002 | 0.0003 | 3.74×10^-5^ | 0.0094 | 0.8948 |
| Size Zone Non Uniformity Normalized (GLSZM) | -0.0199 | -0.0209 | -0.0146 | -0.0066 | 0.0001 | 0.008 |
|  | [-0.0342, -0.0057] | [-0.0351, -0.0067] | [-0.0289, -0.0004] | [-0.0208, 0.0076] | [-0.0147, 0.0148] | [-0.0064, 0.0223] |
|  | 0.0062 | 0.004 | 0.0443 | 0.3611 | 0.9933 | 0.2776 |
| Zone Percentage (GLSZM) | -0.0443 | -0.0364 | -0.0311 | -0.0285 | -0.0398 | -0.01 |
|  | [-0.0579, -0.0306] | [-0.05, -0.0228] | [-0.0448, -0.0175] | [-0.0421, -0.0149] | [-0.0539, -0.0257] | [-0.0237, 0.0038] |
|  | 2.26×10-10 | 1.6×10^-7^ | 7.76×10^-6^ | 4.02×10^-5^ | 3.36×10^-8^ | 0.1557 |
| Grey Level Variance (GLSZM) | -0.0671 | -0.0599 | -0.0408 | -0.0439 | -0.0529 | 0.0094 |
|  | [-0.0812, -0.053] | [-0.074, -0.0459] | [-0.0549, -0.0267] | [-0.058, -0.0299] | [-0.0674, -0.0383] | [-0.0048, 0.0236] |
|  | 1.06×10^-20^ | 5.84×10^-17^ | 1.35×10^-8^ | 7.85×10^-10^ | 1.1×10^-12^ | 0.1926 |
| Zone Variance (GLSZM) | 0.0414 | 0.0332 | 0.0266 | 0.0297 | 0.0383 | 0.0203 |
|  | [0.0292, 0.0536] | [0.0211, 0.0454] | [0.0145, 0.0388] | [0.0176, 0.0418] | [0.0257, 0.0509] | [0.0081, 0.0326] |
|  | 3.09×10^-11^ | 8.66×10^-8^ | 1.83×10^-5^ | 1.61×10^-6^ | 2.68×10^-9^ | 0.0012 |
| Zone Entropy (GLSZM) | -0.0421 | -0.0375 | -0.0232 | -0.0294 | -0.0517 | 0.0092 |
|  | [-0.0562, -0.028] | [-0.0516, -0.0234] | [-0.0373, -0.0091] | [-0.0434, -0.0153] | [-0.0663, -0.0372] | [-0.0051, 0.0234] |
|  | 5.22×10^-9^ | 1.78×10^-7^ | 0.0013 | 4.17×10^-5^ | 3.72×10^-12^ | 0.2067 |
| Low Grey Level Zone Emphasis (GLSZM) | 0.059 | 0.0558 | 0.0361 | 0.0341 | 0.0615 | -0.0129 |
|  | [0.0448, 0.0731] | [0.0418, 0.0699] | [0.022, 0.0502] | [0.02, 0.0481] | [0.0469, 0.0761] | [-0.0272, 0.0013] |
|  | 2.85×10^-16^ | 7.46×10^-15^ | 5.3×10^-7^ | 1.98×10^-6^ | 1.48×10^-16^ | 0.0741 |
| High Grey Level Zone Emphasis (GLSZM) | -0.0713 | -0.0673 | -0.0412 | -0.0436 | -0.0633 | 0.0168 |
|  | [-0.0854, -0.0573] | [-0.0813, -0.0533] | [-0.0553, -0.0272] | [-0.0576, -0.0296] | [-0.0778, -0.0488] | [0.0026, 0.0309] |
|  | 3.13×10^-23^ | 5.2×10^-21^ | 9.05×10^-9^ | 1.05×10^-9^ | 1.48×10^-17^ | 0.0204 |
| Small Area Low Grey Level Emphasis (GLSZM) | 0.0516 | 0.0477 | 0.0304 | 0.0327 | 0.0573 | -0.0135 |
|  | [0.0374, 0.0658] | [0.0335, 0.0618] | [0.0162, 0.0445] | [0.0187, 0.0469] | [0.0426, 0.0719] | [-0.0278, 0.0007] |
|  | 1.03×10^-12^ | 3.93×10^-11^ | 2.66×10^-5^ | 5.36×10^-6^ | 1.91×10-14 | 0.0629 |
| Small Area High Grey Level Emphasis (GLSZM) | -0.0702 | -0.0653 | -0.0414 | -0.0434 | -0.0598 | 0.0164 |
|  | [-0.0842, -0.0561] | [-0.0793, -0.0513] | [-0.0554, -0.0273] | [-0.0574, -0.0294] | [-0.0743, -0.0453] | [0.0022, 0.0305] |
|  | 1.51×10^-22^ | 7.09×10^-20^ | 7.9×10^-9^ | 1.2×10^-9^ | 7.61×10^-16^ | 0.0234 |
| Large Area Low Grey Level Emphasis (GLSZM) | 0.0462 | 0.0399 | 0.0264 | 0.0331 | 0.0522 | 0.0217 |
|  | [0.0322, 0.0601] | [0.026, 0.0538] | [0.0125, 0.0403] | [0.0192, 0.047] | [0.0378, 0.0666] | [0.0077, 0.0358] |
|  | 8.64×10^-11^ | 1.83×10^-8^ | 0.0002 | 2.85×10^-6^ | 1.23×10^-12^ | 0.0024 |
| Large Area High Grey Level Emphasis (GLSZM) | -0.0118 | -0.0158 | -0.0048 | -0.0035 | -0.0143 | 0.0171 |
|  | [-0.0243, 0.0006] | [-0.0282, -0.0034] | [-0.0173, 0.0077] | [-0.0159, 0.0089] | [-0.0272, -0.0015] | [0.0046, 0.0296] |
|  | 0.0626 | 0.0126 | 0.4495 | 0.5841 | 0.0291 | 0.0076 |
| Short Run Emphasis (GLRLM) | -0.0139 | -0.0117 | -0.0086 | -0.0084 | -0.0136 | -0.0036 |
|  | [-0.0274, -0.0005] | [-0.0251, 0.0017] | [-0.022, 0.0048] | [-0.0218, 0.005] | [-0.0275, 0.0004] | [-0.0171, 0.0099] |
|  | 0.0428 | 0.0875 | 0.2097 | 0.2178 | 0.056 | 0.603 |
| Long Run Emphasis (GLRLM) | 0.0177 | 0.0134 | 0.0113 | 0.0133 | 0.0142 | 0.0086 |
|  | [0.0045, 0.0309] | [0.0002, 0.0265] | [-0.0019, 0.0245] | [0.0002, 0.0264] | [0.0006, 0.0279] | [-0.0047, 0.0219] |
|  | 0.0086 | 0.0462 | 0.0931 | 0.047 | 0.0408 | 0.2041 |
| Grey Level Non Uniformity (GLRLM) | 0.0485 | 0.0373 | 0.0317 | 0.0382 | 0.0346 | 0.0265 |
|  | [0.0374, 0.0596] | [0.0262, 0.0484] | [0.0205, 0.0428] | [0.0271, 0.0492] | [0.0231, 0.0461] | [0.0153, 0.0377] |
|  | 1.38×10^-17^ | 4.23×10^-11^ | 2.39×10^-8^ | 1.39×10^-11^ | 3.7×10^-9^ | 3.67×10^-6^ |
| Run Length Non Uniformity (GLRLM) | 0.017 | 0.0096 | 0.0153 | 0.0159 | 0.0093 | 0.0229 |
|  | [0.0036, 0.0304] | [-0.0038, 0.0229] | [0.0019, 0.0286] | [0.0026, 0.0292] | [-0.0045, 0.0231] | [0.0095, 0.0364] |
|  | 0.0128 | 0.1594 | 0.0249 | 0.0191 | 0.1874 | 0.0008 |
| Run Length Non Uniformity Normalized (GLRLM) | -0.0151 | -0.0129 | -0.0092 | -0.0089 | -0.0134 | -0.0029 |
|  | [-0.0286, -0.0015] | [-0.0264, 0.0006] | [-0.0227, 0.0043] | [-0.0223, 0.0046] | [-0.0274, 0.0005] | [-0.0165, 0.0107] |
|  | 0.0291 | 0.0602 | 0.1802 | 0.1953 | 0.0594 | 0.6732 |
| Run Percentage (GLRLM) | -0.0172 | -0.0141 | -0.0108 | -0.0112 | -0.0148 | -0.0044 |
|  | [-0.0306, -0.0038] | [-0.0275, -0.0008] | [-0.0241, 0.0026] | [-0.0244, 0.0021] | [-0.0286, -0.001] | [-0.0178, 0.0091] |
|  | 0.0116 | 0.0371 | 0.1133 | 0.0996 | 0.036 | 0.5238 |
| Grey Level Variance (GLRLM) | -0.0416 | -0.0388 | -0.021 | -0.0281 | -0.0309 | 0.0089 |
|  | [-0.0558, -0.0274] | [-0.053, -0.0247] | [-0.0352, -0.0069] | [-0.0422, -0.014] | [-0.0456, -0.0163] | [-0.0054, 0.0231] |
|  | 9.29×10^-9^ | 7.09×10^-8^ | 0.0036 | 9.53×10^-5^ | 3.49×10^-5^ | 0.2232 |
| Run Variance (GLRLM) | 0.0195 | 0.0149 | 0.0127 | 0.0142 | 0.0133 | 0.009 |
|  | [0.0064, 0.0326] | [0.0019, 0.028] | [-0.0004, 0.0258] | [0.0012, 0.0272] | [-0.0003, 0.0268] | [-0.0042, 0.0222] |
|  | 0.0036 | 0.025 | 0.0565 | 0.0323 | 0.0546 | 0.1793 |
| Run Entropy (GLRLM) | -0.0195 | -0.0187 | -0.0085 | -0.0152 | -0.0191 | 0.0154 |
|  | [-0.0326, -0.0065] | [-0.0317, -0.0057] | [-0.0215, 0.0045] | [-0.0281, -0.0022] | [-0.0325, -0.0056] | [0.0023, 0.0285] |
|  | 0.0034 | 0.0048 | 0.2012 | 0.0217 | 0.0055 | 0.0211 |
| Low Grey Level Run Emphasis (GLRLM) | 0.0567 | 0.0553 | 0.0339 | 0.0311 | 0.0601 | 0.0042 |
|  | [0.0427, 0.0707] | [0.0414, 0.0693] | [0.02, 0.0479] | [0.0172, 0.0451] | [0.0457, 0.0746] | [-0.0099, 0.0183] |
|  | 1.98×10^-15^ | 7.16×10^-15^ | 1.92×10^-6^ | 1.16×10^-5^ | 3.66×10^-16^ | 0.5603 |
| High Grey Level Run Emphasis (GLRLM) | -0.0756 | -0.0729 | -0.0444 | -0.043 | -0.0692 | 0.0113 |
|  | [-0.0895, -0.0618] | [-0.0868, -0.0591] | [-0.0583, -0.0305] | [-0.0569, -0.0292] | [-0.0836, -0.0549] | [-0.0027, 0.0253] |
|  | 1.48×10^-26^ | 5.35×10^-25^ | 3.67×10^-10^ | 1.05×10^-9^ | 3.51×10^-21^ | 0.1134 |
| Short Run Low Grey Level Emphasis (GLRLM) | 0.0543 | 0.0536 | 0.033 | 0.0285 | 0.0583 | 0.0019 |
|  | [0.0404, 0.0682] | [0.0397, 0.0675] | [0.0191, 0.047] | [0.0147, 0.0424] | [0.0439, 0.0727] | [-0.0121, 0.0159] |
|  | 2.34×10^-14^ | 3.86×10^-14^ | 3.32×10^-6^ | 5.51×10^-5^ | 2.11×10^-15^ | 0.7914 |
| Short Run High Grey Level Emphasis (GLRLM) | -0.0733 | -0.0703 | -0.0433 | -0.0418 | -0.0666 | 0.0104 |
|  | [-0.0873, -0.0594] | [-0.0842, -0.0564] | [-0.0573, -0.0293] | [-0.0558, -0.0279] | [-0.0811, -0.0522] | [-0.0037, 0.0245] |
|  | 9.99×10^-25^ | 5.18×10^-23^ | 1.28×10^-9^ | 3.9×10^-9^ | 1.73×10^-19^ | 0.1492 |
| Long Run Low Grey Level Emphasis (GLRLM) | 0.0523 | 0.0474 | 0.0307 | 0.034 | 0.053 | 0.0099 |
|  | [0.0383, 0.0664] | [0.0335, 0.0614] | [0.0167, 0.0447] | [0.0201, 0.048] | [0.0385, 0.0675] | [-0.0042, 0.024] |
|  | 2.78×10^-13^ | 2.97×10^-11^ | 1.74×10^-5^ | 1.77×10^-6^ | 8.25×10^-13^ | 0.1695 |
| Long Run High Grey Level Emphasis (GLRLM) | -0.0526 | -0.0521 | -0.0298 | -0.0295 | -0.0503 | 0.0129 |
|  | [-0.066, -0.0391] | [-0.0655, -0.0388] | [-0.0432, -0.0164] | [-0.0429, -0.0162] | [-0.0642, -0.0364] | [-0.0006, 0.0264] |
|  | 1.7×10^-14^ | 2.11×10^-14^ | 1.35×10^-5^ | 1.44×10^-5^ | 1.18×10^-12^ | 0.0613 |
| Coarseness (NGTDM) | -0.031 | -0.0261 | -0.0241 | -0.0172 | -0.0168 | -0.0175 |
|  | [-0.0432, -0.0188] | [-0.0383, -0.014] | [-0.0362, -0.0119] | [-0.0293, -0.0051] | [-0.0294, -0.0042] | [-0.0298, -0.0053] |
|  | 6.34×10^-7^ | 2.48×10^-5^ | 0.0001 | 0.0054 | 0.0089 | 0.0051 |
| Contrast (NGTDM) | 0.0256 | 0.0231 | 0.0181 | 0.0153 | 0.0372 | -0.0124 |
|  | [0.0115, 0.0398] | [0.009, 0.0372] | [0.004, 0.0322] | [0.0013, 0.0294] | [0.0226, 0.0518] | [-0.0266, 0.0018] |
|  | 0.0004 | 0.0013 | 0.0119 | 0.0324 | 5.9×10^-7^ | 0.0868 |
| Busyness (NGTDM) | 0.0629 | 0.0537 | 0.0422 | 0.0414 | 0.069 | 0.0064 |
|  | [0.0489, 0.0768] | [0.0399, 0.0676] | [0.0283, 0.0561] | [0.0275, 0.0552] | [0.0547, 0.0834] | [-0.0076, 0.0204] |
|  | 9.47×10^-19^ | 3.25×10^-14^ | 2.73×10^-9^ | 4.88×10^-9^ | 5.45×10^-21^ | 0.3726 |
| Complexity (NGTDM) | -0.054 | -0.0472 | -0.0317 | -0.0381 | -0.0465 | 0.0093 |
|  | [-0.0683, -0.0398] | [-0.0613, -0.033] | [-0.0459, -0.0175] | [-0.0522, -0.0239] | [-0.0612, -0.0318] | [-0.005, 0.0236] |
|  | 9.63×10^-14^ | 6.84×10^-11^ | 1.23×10^-5^ | 1.31×10^-7^ | 5.72×10^-10^ | 0.2029 |
| Strength (NGTDM) | -0.0549 | -0.0482 | -0.0311 | -0.0394 | -0.045 | 0.0074 |
|  | [-0.0691, -0.0407] | [-0.0623, -0.0341] | [-0.0453, -0.017] | [-0.0535, -0.0253] | [-0.0596, -0.0303] | [-0.0069, 0.0217] |
|  | 3.26×10^-14^ | 2.28×10^-11^ | 1.63×10^-5^ | 4.39×10^-8^ | 1.81×10^-9^ | 0.3089 |
| Small Dependence Emphasis (GLDM) | -0.0422 | -0.0342 | -0.0296 | -0.0276 | -0.0353 | -0.0082 |
|  | [-0.0557, -0.0287] | [-0.0477, -0.0208] | [-0.043, -0.0161] | [-0.041, -0.0142] | [-0.0492, -0.0214] | [-0.0218, 0.0053] |
|  | 9.06×10^-10^ | 5.92×10^-7^ | 1.68×10^-5^ | 5.46×10^-5^ | 7×10^-7^ | 0.2349 |
| Large Dependence Emphasis (GLDM) | 0.0152 | 0.0127 | 0.0093 | 0.0097 | 0.013 | 0.0043 |
|  | [0.0019, 0.0286] | [-0.0006, 0.0261] | [-0.004, 0.0227] | [-0.0036, 0.0229] | [-0.0008, 0.0268] | [-0.0092, 0.0177] |
|  | 0.0255 | 0.0606 | 0.1711 | 0.1545 | 0.0645 | 0.532 |
| Grey Level Non Uniformity (GLDM) | 0.0423 | 0.0324 | 0.0269 | 0.0337 | 0.0284 | 0.0214 |
|  | [0.0308, 0.0538] | [0.0209, 0.0439] | [0.0155, 0.0384] | [0.0222, 0.0451] | [0.0165, 0.0403] | [0.0098, 0.033] |
|  | 6.03×10^-13^ | 3.11×10^-8^ | 4.34×10^-6^ | 8.11×10^-9^ | 2.85×10^-6^ | 0.0003 |
| Dependence Non Uniformity (GLDM) | 0.0313 | 0.0213 | 0.0248 | 0.0251 | 0.0221 | 0.0267 |
|  | [0.02, 0.0427] | [0.01, 0.0326] | [0.0135, 0.0361] | [0.0138, 0.0363] | [0.0104, 0.0338] | [0.0153, 0.0381] |
|  | 5.65×10^-8^ | 0.0002 | 1.63×10^-5^ | 1.24×10^-5^ | 0.0002 | 4.32×10^-6^ |
| Dependence Non Uniformity Normalized (GLDM) | -0.0098 | -0.0082 | -0.0053 | -0.0067 | -0.0036 | -0.0048 |
|  | [-0.0237, 0.004] | [-0.022, 0.0056] | [-0.0191, 0.0085] | [-0.0205, 0.007] | [-0.0179, 0.0108] | [-0.0187, 0.0091] |
|  | 0.165 | 0.2424 | 0.4514 | 0.3387 | 0.6262 | 0.4968 |
| Grey Level Variance (GLDM) | -0.0346 | -0.0321 | -0.0178 | -0.0233 | -0.0244 | 0.0079 |
|  | [-0.0488, -0.0204] | [-0.0463, -0.018] | [-0.032, -0.0037] | [-0.0374, -0.0092] | [-0.0391, -0.0097] | [-0.0064, 0.0222] |
|  | 1.75×10^-6^ | 8.45×10^-6^ | 0.0137 | 0.0012 | 0.0011 | 0.2776 |
| Dependence Variance (GLDM) | 0.0074 | 0.0064 | 0.0047 | 0.0041 | 0.0025 | 0.0039 |
|  | [-0.0064, 0.0212] | [-0.0074, 0.0201] | [-0.0091, 0.0184] | [-0.0096, 0.0178] | [-0.0118, 0.0167] | [-0.01, 0.0177] |
|  | 0.2908 | 0.3647 | 0.5062 | 0.5573 | 0.7314 | 0.5831 |
| Dependence Entropy (GLDM) | -0.0143 | -0.0102 | -0.008 | -0.0142 | -0.0151 | 0.0115 |
|  | [-0.0281, -0.0005] | [-0.024, 0.0036] | [-0.0218, 0.0057] | [-0.028, -0.0005] | [-0.0293, -0.0008] | [-0.0024, 0.0254] |
|  | 0.0427 | 0.1459 | 0.2526 | 0.0419 | 0.0385 | 0.1037 |
| Low Grey Level Emphasis (GLDM) | 0.0565 | 0.0546 | 0.0335 | 0.0323 | 0.0591 | 0.0059 |
|  | [0.0426, 0.0705] | [0.0407, 0.0685] | [0.0195, 0.0474] | [0.0184, 0.0462] | [0.0447, 0.0735] | [-0.0082, 0.0199] |
|  | 2.27×10^-15^ | 1.49×10^-14^ | 2.61×10^-6^ | 5.31×10^-6^ | 1.02×10^-15^ | 0.4136 |
| High Grey Level Emphasis (GLDM) | -0.0763 | -0.0731 | -0.045 | -0.0439 | -0.0701 | 0.0104 |
|  | [-0.0901, -0.0624] | [-0.0869, -0.0593] | [-0.0588, -0.0311] | [-0.0577, -0.0301] | [-0.0844, -0.0558] | [-0.0035, 0.0244] |
|  | 4.86×10^-27^ | 3.84×10^-25^ | 2.08×10^-10^ | 4.54×10^-10^ | 9.82×10^-22^ | 0.1429 |
| Small Dependence Low Grey Level Emphasis (GLDM) | 0.0412 | 0.0417 | 0.0234 | 0.0223 | 0.0432 | -0.013 |
|  | [0.0274, 0.0549] | [0.0281, 0.0554] | [0.0097, 0.0371] | [0.0087, 0.036] | [0.029, 0.0574] | [-0.0268, 0.0008] |
|  | 4.27×10^-9^ | 2.2×10^-9^ | 0.0008 | 0.0014 | 2.44×10^-9^ | 0.0652 |
| Small Dependence High Grey Level Emphasis (GLDM) | -0.0673 | -0.0604 | -0.041 | -0.043 | -0.0572 | 0.0058 |
|  | [-0.0814, -0.0532] | [-0.0745, -0.0464] | [-0.0551, -0.027] | [-0.057, -0.029] | [-0.0717, -0.0427] | [-0.0084, 0.02] |
|  | 7.74×10^-21^ | 3.08×10^-17^ | 1.1×10^-8^ | 1.69×10^-9^ | 1.31×10^-14^ | 0.4222 |
| Large Dependence Low Grey Level Emphasis (GLDM) | 0.0555 | 0.0514 | 0.0319 | 0.0355 | 0.0547 | 0.008 |
|  | [0.0415, 0.0695] | [0.0374, 0.0653] | [0.0179, 0.0459] | [0.0216, 0.0494] | [0.0402, 0.0691] | [-0.0061, 0.0221] |
|  | 8.06×10^-15^ | 5.36×10^-13^ | 7.73×10-6 | 5.93×10^-7^ | 1.31×10^-13^ | 0.2651 |
| Large Dependence High Grey Level Emphasis (GLDM) | -0.0587 | -0.0569 | -0.034 | -0.0343 | -0.0545 | 0.0102 |
|  | [-0.0724, -0.0451] | [-0.0705, -0.0434] | [-0.0476, -0.0204] | [-0.0478, -0.0207] | [-0.0686, -0.0405] | [-0.0035, 0.0239] |
|  | 2.92×10^-17^ | 1.99×10^-16^ | 9.87×10^-7^ | 7.1×10^-7^ | 3.14×10^-14^ | 0.1433 |

**Supplementary Table 12 footnote:** Each cell represents a separate model, adjusted for: age, sex, social deprivation, educational level, smoking, alcohol intake, exercise level. Results are degree of change in radiomics shape feature per 100g increase in daily meat/fish consumption with corresponding 95% confidence intervals and p-values. First, second, and third row for every CMR measures corresponds to beta coefficient, 95% confidence interval and p-value, respectively. Bonferroni adjusted significance threshold p-value =0.0001 (corrected for 432 comparisons). GLCM: grey level co-occurrence matrix; GLDM: grey level dependence matrix; GLRLM: grey level run length matrix; GLSZM: grey level size zone matrix; NGTDM: neighboring grey tone difference matrix; LV: left ventricle.

**Supplementary Table 13. Baseline population characteristics (ASI at baseline)**

| Population characteristic | Frequency/mean (standard deviation) |
| --- | --- |
| Male  Female | 76,989 (45.9%)  90,525 (54.1%) |
| Age (years) | 56.7 (±8.2) |
| Townsend deprivation index** | -1.10 (±3.0) |
| Body mass index (kg/m^2^) | 27.5 (±4.8) |
| Smoking (current smoker) | 16,682 (10.0%) |
| Diabetes | 10,795 (6.4%) |
| Hypertension | 35,514 (21.2%) |
| Hypercholesterolaemia | 49,476 (29.6%) |
| IPAQ score (METS/week) | 1593.00 [2644.50] |
| Educational level^*^:  Left school age 14 or younger without qualifications  Left school age 15 or older without qualifications  High school diploma  Sixth form qualification  Professional qualification  Higher education university degree | 1,323 (0.8%)  24,545 (14.7%)  28,249 (16.9%)  9,506 (5.7%)  45,817 (27.4%)  56,158 (33.5%) |
| Alcohol intake frequency:  Never  Special occasions only  1-3 times a month  1-2 times a week  3-4 times a week  Daily or almost daily | 14,745 (8.8%)  20,461 (12.2%)  18,933 (11.3%)  41,901 (25.0%)  37,095 (22.2%)  33,743 (20.2%) |
| Unprocessed red meat intake (grams/day):  Beef  Lamb  Pork | 22.4 (±16.32)  9.1 (±9.0)  6.6 (±6.3)  6.8 (±6.8) |
| Processed meat intake (grams/day) | 16.4 (±15.9) |
| Oily fish intake (grams/day) | 12.1 (±11.6) |

**Supplementary Table 13 footnote:** Results are frequencies and percentages for categorical variables and mean standard deviation for continuous variable. *High school diploma includes: O levels, GCSE, CSE, or equivalent; Sixth form qualification includes: A levels/AS levels, or equivalent; professional qualifications refer to nursing, teaching, or equivalent. I IPAQ: international physical activity questionnaire; METS: metabolic equivalents.** **Townsend index: zero, positive, and negative scores indicate average, higher, and lower levels of material deprivation respectively relative to UK national averages.

**Supplementary Table 14. Multivariable linear regression models showing change of arterial compliance measures per 100g increase in daily meat/fish consumption.**

|  | AD  (mm $\times$10^-3^) | ASI  (baseline, m/s) | ASI  (imaging, m/s) | Interval change in ASI (baseline-imaging, m/s) |
| --- | --- | --- | --- | --- |
| Unprocessed red meat | -0.057 | 0.487* | 0.349* | 0.150* |
|  | [-0.130, 0.015] | [0.405, 0.569] | [0.151, 0.546] | [0.026, 0.274] |
|  | 0.1207 | 2.26$\times$10^-31^ | 5.46$\times$10^-4^ | 0.0181 |
| Beef | -0.123* | 0.681* | 0.526* | 0.240* |
|  | [-0.245, -0.001] | [0.534, 0.828] | [0.193, 0.859] | [0.024, 0.457] |
|  | 0.0476 | 1.19$\times$10^-19^ | 0.002 | 0.0295 |
| Lamb | -0.019 | 0.891* | 0.415 | 0.223 |
|  | [-0.225, 0.186] | [0.679, 1.103] | [-0.140, 0.970] | [-0.132, 0.579] |
|  | 0.8538 | 1.86$\times$10^-16^ | 0.1431 | 0.2183 |
| Pork | -0.083 | 0.834* | 0.722* | 0.260 |
|  | [-0.268, 0.102] | [0.637, 1.032] | [0.222, 1.222] | [-0.047, 0.567] |
|  | 0.3805 | 1.32$\times$10^-16^ | 0.0046 | 0.0968 |
| Processed meat | -0.000 | 0.445* | 0.224* | 0.049 |
|  | [-0.076, 0.076] | [0.359, 0.531] | [0.021, 0.427] | [-0.071, 0.168] |
|  | 0.9971 | 4.47$\times$10^-24^ | 0.0309 | 0.4275 |
| Oily fish | 0.013 | -0.220* | -0.432* | -0.167 |
|  | [-0.090, 0.115] | [-0.335, -0.105] | [-0.708, -0.155] | [-0.341, 0.007] |
|  | 0.8093 | 1.70$\times$10^-4^ | 0.0022 | 0.0602 |

**Supplementary Table 14 footnote:** Each cell represents a separate model, adjusted for: age, sex, social deprivation, educational level, smoking, alcohol intake, and exercise level (confounder adjusted model). For ‘interval change in ASI’, results are average standard deviation change from that expected from baseline. First, second, and third row for every CMR measures corresponds to beta coefficient, 95% confidence interval and p-value, respectively. AD: aortic distensibility; ASI: arterial stiffness index. *indicates p-value <0.05.

**Supplementary Table 15. Multivariable linear regression models showing change of arterial compliance measures per 100g increase in daily meat/fish consumption (confounders + covariates potentially on causal pathway)**

|  | AD  (mm $\times$10^-3^) | ASI  (baseline, m/s) | ASI  (imaging, m/s) | Interval change ASI (baseline-imaging, m/s) |
| --- | --- | --- | --- | --- |
| Unprocessed red meat | -0.048 | 0.274* | 0.203* | 0.102 |
|  | [-0.121, 0.024] | [0.191, 0.356] | [0.004, 0.402] | [-0.022, 0.226] |
|  | 0.1919 | 7.15$\times$10^-11^ | 0.0457 | 0.1075 |
| Beef | -0.108 | 0.374* | 0.311 | 0.173 |
|  | [-0.230, 0.014] | [0.227, 0.521] | [-0.023, 0.646] | [-0.044, 0.389] |
|  | 0.0834 | 6.43$\times$10^-7^ | 0.0683 | 0.1173 |
| Lamb | -0.018 | 0.551* | 0.159 | 0.123 |
|  | [-0.224, 0.188] | [0.339, 0.763] | [-0.399, 0.716] | [-0.232, 0.479] |
|  | 0.8658 | 3.53$\times$10^-7^ | 0.5774 | 0.4966 |
| Pork | -0.060 | 0.429* | 0.472 | 0.180 |
|  | [-0.245, 0.125] | [0.231, 0.626] | [-0.029, 0.972] | [-0.127, 0.487] |
|  | 0.5258 | 2.11$\times$10^-5^ | 0.0649 | 0.2516 |
| Processed meat | 0.011 | 0.249* | 0.108 | 0.020 |
|  | [-0.065, 0.087] | [0.163, 0.335] | [-0.096, 0.312] | [-0.100, 0.140] |
|  | 0.7685 | 1.56$\times$10^-8^ | 0.3005 | 0.7449 |
| Oily fish | 0.014 | -0.254* | -0.434* | -0.181* |
|  | [-0.088, 0.116] | [-0.368, -0.140] | [-0.710, -0.158] | [-0.355, -0.007] |
|  | 0.7850 | 1.31$\times$10^-5^ | 0.0021 | 0.0418 |

**Supplementary Table 15:** Each cell represents a separate model, adjusted for: age, sex, social deprivation, educational level, smoking, alcohol intake, exercise level, body mass index, hypertension, hypercholesterolaemia, diabetes (mediator adjusted models). For ‘interval change in ASI’, results are average standard deviation change from that expected from baseline. First, second, and third row for every CMR measures corresponds to beta coefficient, 95% confidence interval and p-value, respectively. AD: aortic distensibility; ASI: arterial stiffness index. *indicates p-value <0.05
