# Supplementary material for: Associations of Meat and Fish Consumption With Conventional and Radiomics Cardiovascular Magnetic Resonance Phenotypes in the UK Biobank
Source: Front Cardiovasc Med. 2021 May 5;8:667849. doi: 10.3389/fcvm.2021.667849 (PMC8133433; doi:10.3389/fcvm.2021.667849)
Supplement: Supplementary file 2 [file Data_Sheet_2.docx]

**
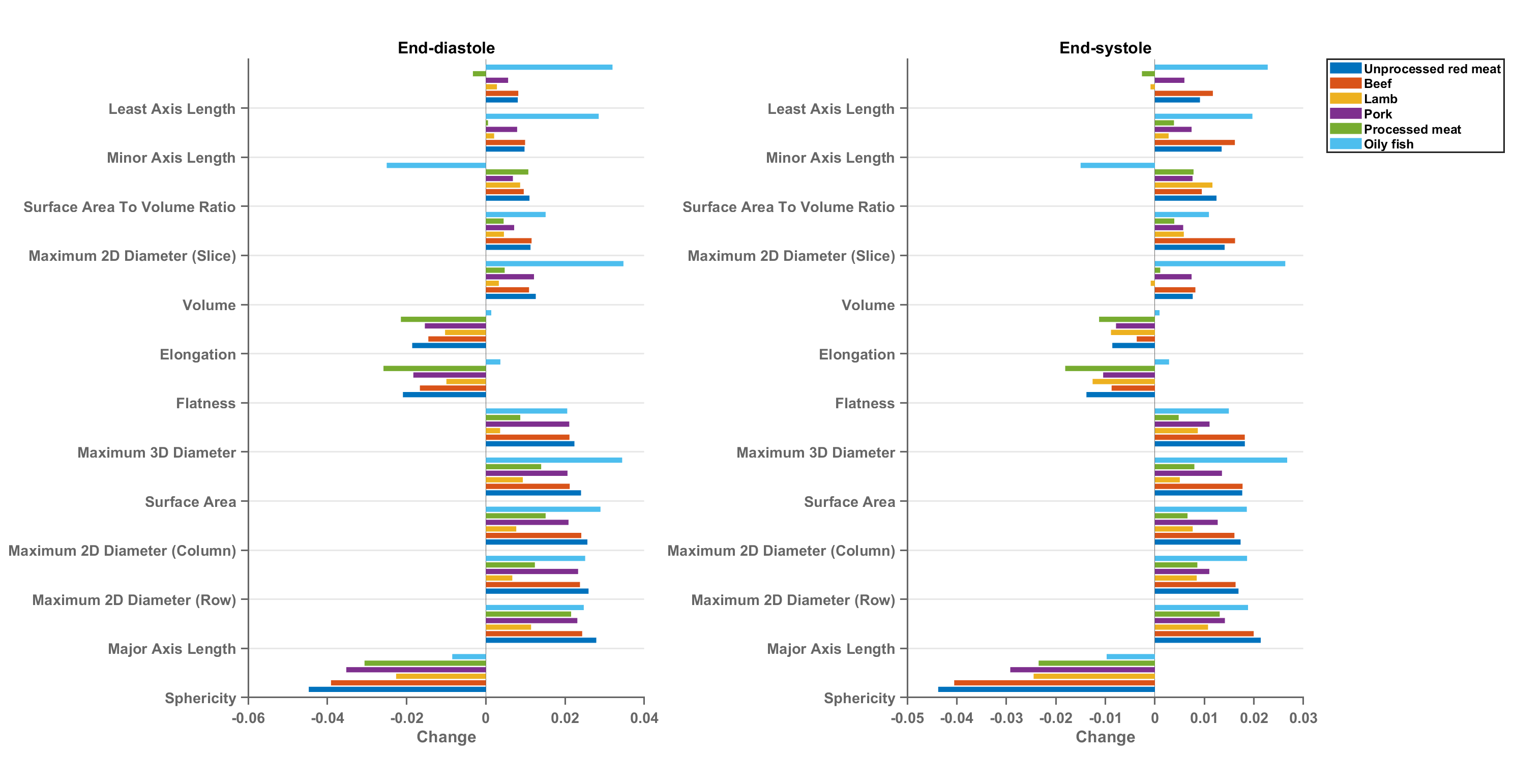
Supplementary Figure 1. Multivariable linear regression models showing change in LV CMR shape radiomics per 100g increase in daily meat consumption**

**Supplementary Figure 1 footnote:** Each bar represents standardised beta coefficients corresponding to the indicated radiomics shape feature. Each bar is from a separate model adjusted for age, sex, social deprivation, educational level, smoking, alcohol intake, exercise level (confounder adjusted model). CMR: cardiovascular magnetic resonance; LV: left ventricle.

**Supplementary figure 2. Multivariable linear regression models showing change in RV CMR shape radiomics per 100g increase in daily meat consumption**


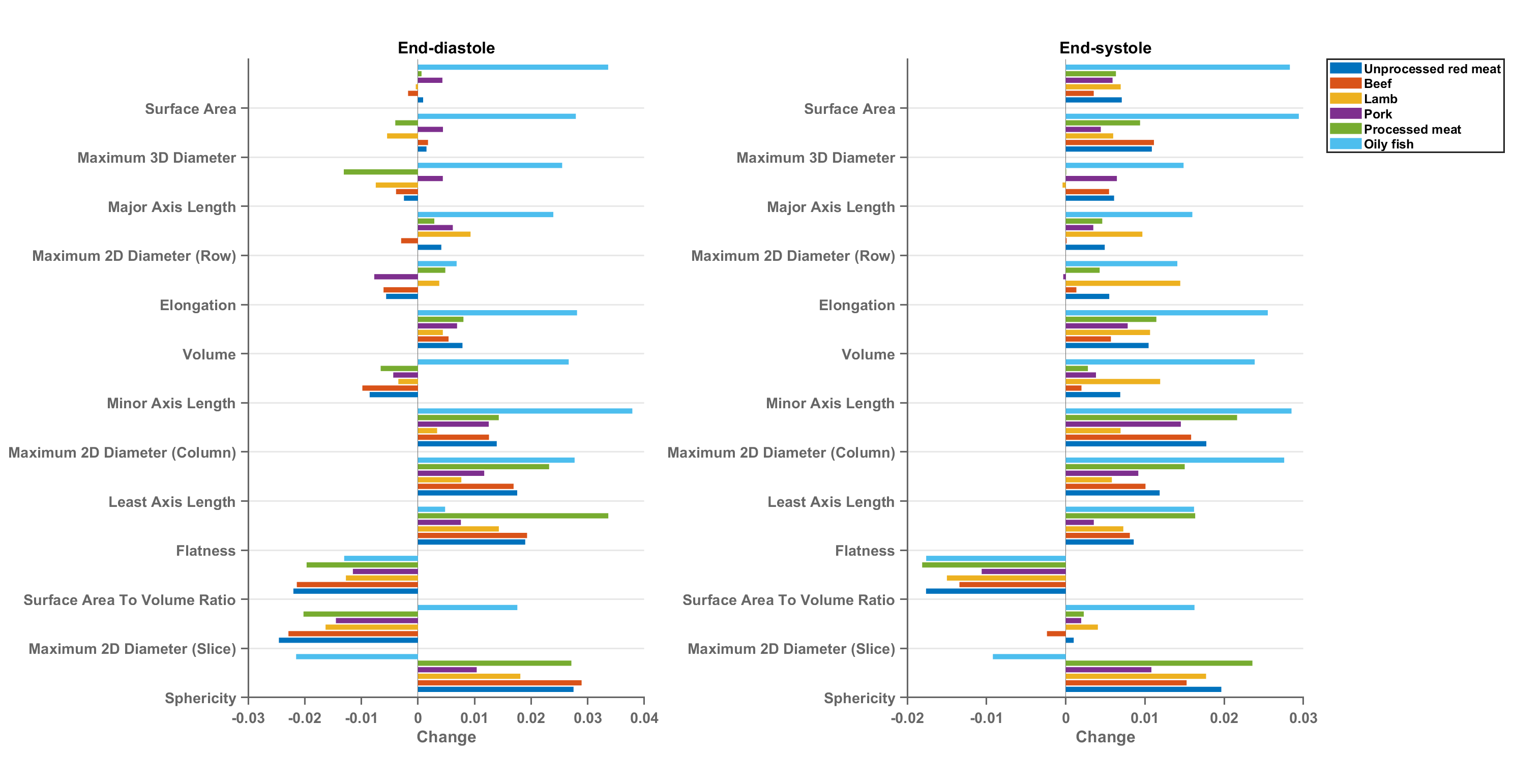
**Supplementary Figure 2 footnote:** Each bar represents standardised beta coefficients corresponding to the indicated radiomics shape feature. Each bar is from a separate model adjusted for age, sex, social deprivation, educational level, smoking, alcohol intake, exercise level (confounder adjusted model). Black lines represent half-length of confidence interval for the corresponding bar. CMR: cardiovascular magnetic resonance; RV: right ventricle.

**Supplementary figure 3. Multivariable linear regression models showing change in myocardium CMR first-order radiomics per 100g increase in daily meat consumption**

**
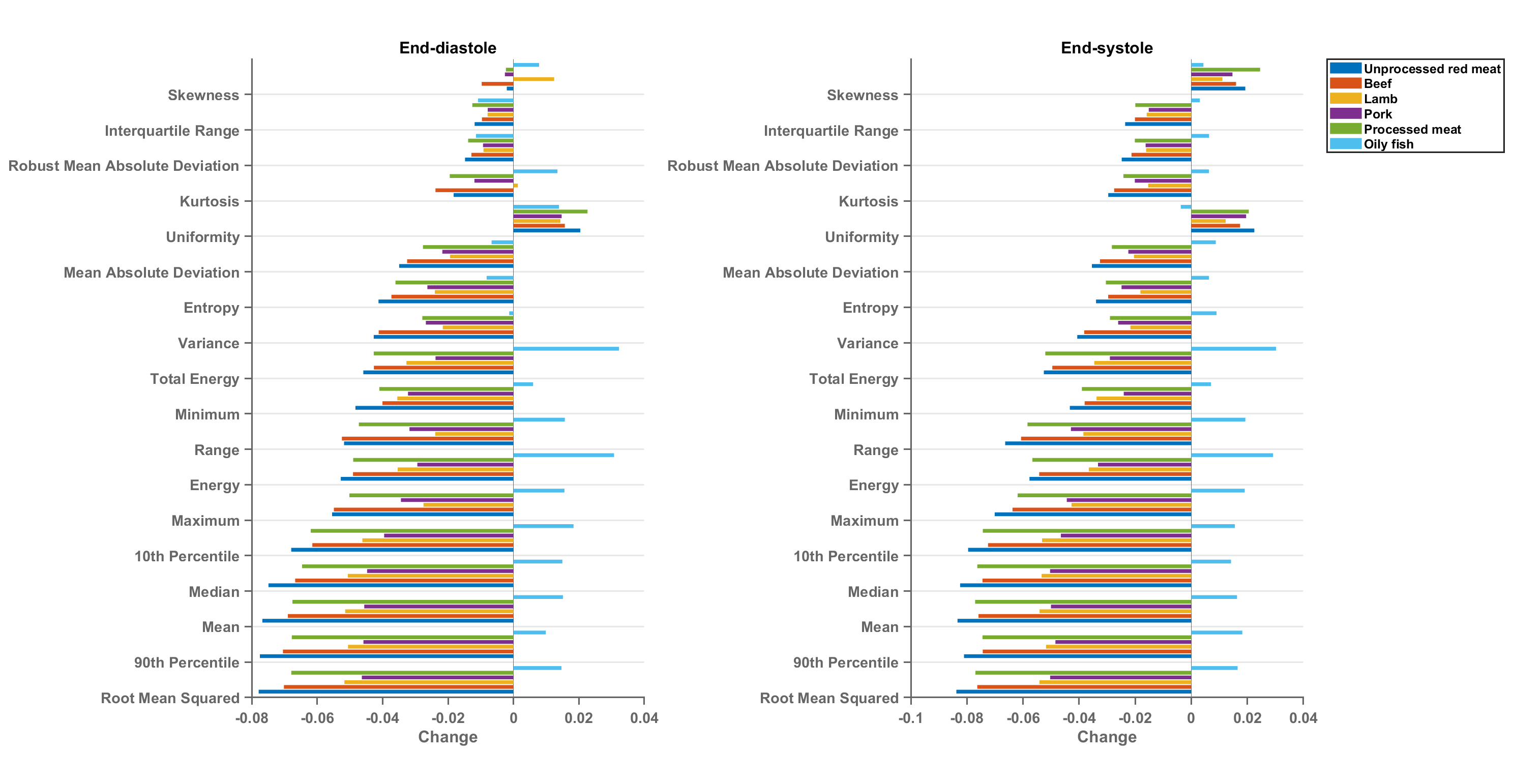
**

**Supplementary figure 3 footnote:** Features are in descending order of change in unprocessed red meat in end-diastole. Each bar is from a separate model adjusted for age, sex, social deprivation, educational level, smoking, alcohol intake, exercise level (confounder adjusted model).
