# Supplementary material for: Associations of Meat and Fish Consumption With Conventional and Radiomics Cardiovascular Magnetic Resonance Phenotypes in the UK Biobank
Source: Front Cardiovasc Med. 2021 May 5;8:667849. doi: 10.3389/fcvm.2021.667849 (PMC8133433; doi:10.3389/fcvm.2021.667849)
Supplement: Supplementary file 3 [file Data_Sheet_3.docx]

**Brief guide to cardiac magnetic resonance radiomics**

Cardiac magnetic resonance (CMR) radiomics is a novel image analysis technique permitting computation of multiple indices of shape and texture within defined regions of interest (ROI). The full list of radiomics features considered in this study can be found in Supplementary table 2, all features have standardised mathematical definitions and have been calculated using the latest version of the open access Pyradiomics platform.

**Radiomics shape features**

Shape features were extracted from three ROIs (right ventricular cavity, left ventricular cavity, left ventricular myocardium), defined by segmentation of standard short axis cine images in end-systole and end-diastole from base to apex.The contours are used to build 3D masks of the ROIs, from which radiomics shape features may be extracted. Radiomics shape features include standard measures such as volume and more advanced shape measures such as surface area and sphericity. Thus, CMR radiomics shape features allow more detailed geometric characterisation of the LV and RV cavities and the LV myocardium than conventional image analysis metrics.

**Figure 1. Regions of interest are defined from standard short axis cardiac magnetic resonance images and used to build a 3D image mask for radiomics analysis**


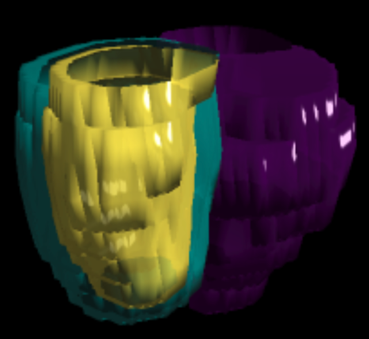

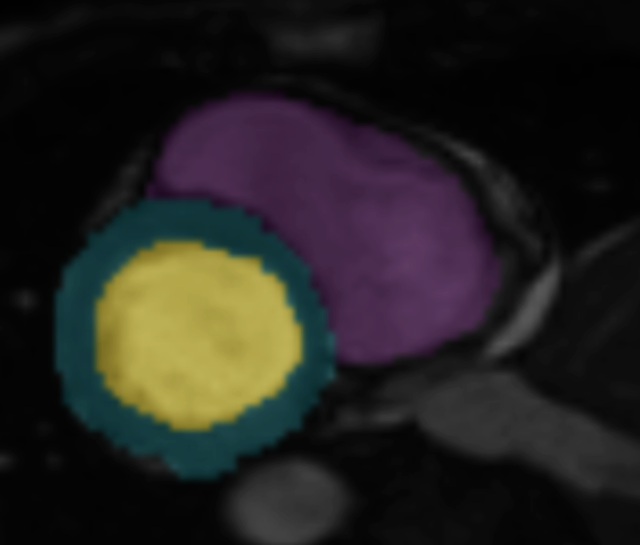


Figure 1 caption: image adapted from Raisi-Estabragh et al.[1]

**Radiomics signal intensity based features**

Radiomics signal intensity based features permit quantification of the pixel signal intensity patterns within a selected ROI. In CMR radiomics, texture analysis is typically applied to the left ventricular myocardium. It is hypothesised that analysing the visual appearance of the myocardium in this way can help us identify subtle differences between healthy and diseased individuals. Radiomics signal intensity based features include first order and texture features. First order features summarise the global distribution of voxel signal intensities within the defined ROI, they rely on histogram based statistics such as mean, median, standard deviation and skewness (full list, supplementary Table 2). Texture features are more complicated calculations which quantify the relationship between neighbouring voxel signal intensities within the ROI and so numerically describe the pattern of signal intensities within the ROI.

**How do I interpret the radiomics results in this study?**

In this study the association of radiomics features with meat/fish exposures was calculated using separate multivariate linear regression models for each feature. The results are presented as the standardised beta coefficient associated with each radiomics feature, permitting comparison across different features. The detailed numerical results for association of the different meat exposures and all individual radiomics features are presented in supplementary tables 5 to 12. For ease of interpretation, the results are also summarised in Figure format in the main manuscript. The results for shape and first-order features are presented in Figure 3, Figure 4, and Supplementary Figures 1-2. The coloured bars in these figures represent standardised beta coefficients associated with each radiomics feature corresponding to different dietary exposures (please refer to figure legends). The direction and magnitude of change can be visually appreciated by looking at the size and direction of the individual bars and comparing these across the different exposure categories for the different exposures.

As there were a large number of texture features (144 extracted per ROI), the patterns in these features cannot be summarised in a single figure. We thus summarise these results in two ways. First, we summarise results from texture features that had opposite direction of effect in the different exposure groups (Figure 5, Supplementary Figure 4). Second, we performed cluster analysis to reduce the 144 individual features into seven clusters incorporating the most defining features (Table 3, Figure 1). In this way, we were able to look at the overall picture of texture features through these seven clusters (Figure 5). Interpretation of the figure is as previously described, with bars representing standardised beta-coefficients for each cluster.

[1]. Raisi-Estabragh Z, Izquierdo C, Campello VM, Martin-isla C, Jaggi A, Harvey NC, Lekadir K, Petersen SE. Cardiac magnetic resonance radiomics: basic principles and clinical perspectives. Eur. Hear. J. - Cardiovasc. Imaging. 2020;1–8.
